# A method of predicting changes in human gene splicing induced by genetic variants in context of cis-acting elements

**DOI:** 10.1186/1471-2105-11-22

**Published:** 2010-01-12

**Authors:** Alexander Churbanov, Igor Vořechovský, Chindo Hicks

**Affiliations:** 1 New Mexico State University, Biology Dept., MSC 3AF, PO Box 30001, Las Cruces, NM 88003, USA; 2 University of Southampton, Southampton University Hospital, MP808, Tremona Road, Southampton SO16 6YD, UK; 3 Loyola University Medical Center, 2160 S. First Ave., Maywood, IL 60153, USA

## Abstract

**Background:**

Polymorphic variants and mutations disrupting canonical splicing isoforms are among the leading causes of human hereditary disorders. While there is a substantial evidence of aberrant splicing causing Mendelian diseases, the implication of such events in multi-genic disorders is yet to be well understood. We have developed a new tool (SpliceScan II) for predicting the effects of genetic variants on splicing and cis-regulatory elements. The novel Bayesian non-canonical 5'GC splice site (SS) sensor used in our tool allows inference on non-canonical exons.

**Results:**

Our tool performed favorably when compared with the existing methods in the context of genes linked to the Autism Spectrum Disorder (ASD). SpliceScan II was able to predict more aberrant splicing isoforms triggered by the mutations, as documented in DBASS5 and DBASS3 aberrant splicing databases, than other existing methods. Detrimental effects behind some of the polymorphic variations previously associated with Alzheimer's and breast cancer could be explained by changes in predicted splicing patterns.

**Conclusions:**

We have developed SpliceScan II, an effective and sensitive tool for predicting the detrimental effects of genomic variants on splicing leading to Mendelian and complex hereditary disorders. The method could potentially be used to screen resequenced patient DNA to identify *de novo *mutations and polymorphic variants that could contribute to a genetic disorder.

## Background

Human pre-mRNA sequences are subjected to complex multi-stage modifications by splicing, where frequent variations in this process contribute to the proteome diversity. During splicing the intronic sequences are recognized and excised by the spliceosome, where the relatively short exonic sequences are joined together to form mature mRNA. The Splice Site (SS) signals at the intronic 5' end (donor) and 3' end (acceptor, polypyrimidine tract and the branch point) are necessary, but not sufficient for accurate and efficient exon recognition by the spliceosome [1,2]. Additional exon-proximal elements are required for proper recognition of weakly defined or alternatively committed exons [3]. These cis-acting elements include a repertoire of Exonic Splicing Enhancers (ESEs) and Intronic Splicing Enhancers (ISEs) along with a number of Exonic Splicing Silencers (ESSs) and Intronic Splicing Silencers (ISSs). The evolutionary fine-tuned antagonism between enhancing and silencing elements leads to the proper splicing of human pre-mRNAs. Mutations disrupting cis-acting elements and SSs themselves, as well as mutations creating cryptic SSs and cis-acting factor binding sites can lead to severe diseases [4].

Mutations affecting alternative and constitutive splicing play a major role in human hereditary disorders [5]. More than 5,477 splicing mutations (as of July 2008) have been documented in the HGMD database [6], which makes this group of mutations one of the most frequent disease-causing alterations. Databases DBASS5 [7] and DBASS3 [8] contain 431 and 283 well annotated disease-causing aberrant splicing events, respectively. A clear understanding of elements affecting splicing could potentially aid diagnosis and development of novel therapeutic strategies [9,10].

Since alternations in splicing are ubiquitous among human multi-exonic genes [11], it is important to understand the key regulators of this process. The 5'GC SSs, flanking <1% of human exons [12], were shown to play an important role in the genesis of alternative splicing in human genes [13] and were found to accumulate in mammalian lineage [14]. The majority of 5'GC SS sensors, i.e. computational procedures reporting how well an oligonucleotide would play a role of a SS, built up to date is based on weight matrices [12,15]. Being an elusive signal, it is difficult to collect a representative learning set that would facilitate building a stronger model. The importance of proper modeling the 5'GC SS comes from the observation that some mutations documented in DBASS5 [7], such as IVS27+3_6dup(GGGT)(-96), IVS7+1G>T(-40), IVS9+1G>A(-45) and others trigger use of cryptic non-canonical 5'GC SS. Despite of the importance of this splicing signal only few splicing prediction methods, such as GeneSequer [16] and NetGene2 [17], are able to score non-canonical exons.

Human introns contain many decoy exons that are similar to authentic exons, but are never committed by the spliceosome and outnumber the real exons by an order of magnitude [18]. The mechanisms that allow accurate discrimination between decoy exons and their authentic counterparts are poorly understood. Codon sequence contained in coding exons have particular 3-periodic compositional biases [19] that allow gene finders, such as GenScan[20] and HMMgene[21], stitching putative coding exons in a frame-consistent fashion with high accuracy [22]. However, methods that rely on protein coding potential features experience severe performance loss when confronted with non-coding exons [23,24]. On the other hand, human mutations frequently create *de novo *cryptic exons with no apparent coding potential leading to severe disorders caused by aberrant splicing [7,8]. Therefore, tools are needed to explain the effects of mutations in terms of signals associated with splicing free of protein coding context [3].

Investigation whether prediction of SSs could be accomplished without relying on protein coding potential started with simple tools such as SpliceView[25] and GENIO[26]. The NetUTR[24] tool has been specifically constructed to predict SSs in 5' untranslated regions (UTRs), therefore addressing the problem of splicing prediction without relying on protein coding features. Maximum Entropy Sensor [27] has been found to be one of the most sensitive diagnostic methods predicting the effects of mutations in human genes [7,8,28]. ExonScan[29], a tool built around the exon definition model, combines the power of the Maximum Entropy Sensor with the Logarithm of Odds (LOD) biases associated with the previously reported ESEs [30], ESSs [29] and poly-G runs (known ISEs [31]). Recent CRYP-SKIP[32,33] tool is based on multivariate logistic discrimination procedure that distinguishes the two aberrant splicing outcomes from DNA sequences. Bayesian SS sensor [23], shown to outperform the Maximum Entropy Sensor [34], is an integral part of the SpliceScan tool [23], built around the SS definition model supported by the enhancers predicted with the MHMMotif tool [23] and various other previously reported silencing and enhancing signals. The SpliceScan has been found to be especially efficient on the test set of short 5' UTR fragments.

We introduce a new tool SpliceScan II built around the exon definition model [1]. Unlike in previous SpliceScan[23] method, the new tool has option of displaying factors contributing to a score assigned to a specific exon isoform thus informing medical practitioners of possible changes in splicing commitment caused by polymorphic variants and mutations. We have used a much larger set of orthologous exons originating from 23 *Tetrapoda *organisms to train the new splicing model following an observation that the spliceosomal and cis-acting factors stay mostly intact across vertebrates [4,35-38], where the genes encoding well-known RNA binding proteins involved in splicing regulation are enriched with ultraconserved elements [39]. The SpliceScan II tool is based on the Bayesian SS sensors, and uses the novel set of enhancer and silencer elements computationally predicted in *Tetrapoda *organisms [40]. Having a large collection of *Tetrapoda *orthologous exons we were able to collect learning set of 5'GC SSs, representative enough to train a new Bayesian 5'GC SS sensor, used in our tool. We compared the performance of our tool with other methods on gene fragments annotated in DBASS5 [7] and DBASS3 [8] and gene structures linked to Autism Spectrum Disorder (ASD). We further evaluated the method by predicting the effects on splicing for some of the previously reported polymorphisms associated with Alzheimer's and the Breast cancer, suggesting possible mechanism causing the disease predisposition associated with such variants.

## Results and Discussion

### Predicting aberrant splicing isoforms

**Figure 1:**
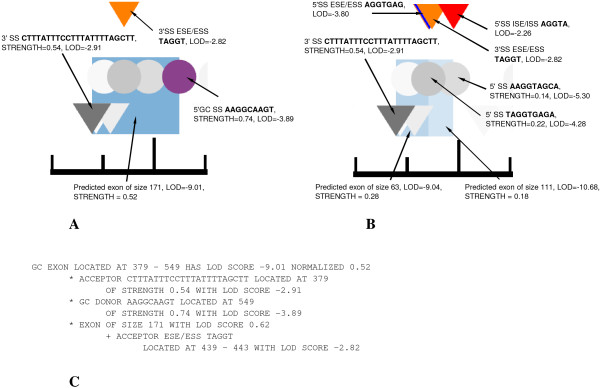
**An example **SpliceScan II** output predicting an effect of mutation**. IVS2+2delC annotated in DBASS5 [7] as causing familial pulmonary arterial hypertension [56], a single nucleotide deletion which disrupts a strong non-canonical 5'GC SS (shown as purple circle) and causes activation of two cryptic alternatively committed canonical 5'SSs located -60 and -108 nucleotides upstream of the original SS. Here we successfully predict an effect of mutation on the original allele shown in (A), where two alternatively used aberrant exonic isoforms activated as shown in (B). SpliceScan II predicted 3'SSs are represented as black-and-white triangles, 5'SSs are black-and-white circles, predicted exons are shown as blue rectangles. The more intense the color a displayed signal, the higher its predicted strength. In (C) we show an example of SpliceScan II textual output listing factors contributing to non-canonical GC exon score assignment shown in (A).

As a first step, we predicted the effects of mutations on splicing. Figure 1 shows an example of SpliceScan II predicted aberrant splicing events induced by the IVS2+2delC mutation causing familial arterial hypertension as annotated in DBASS5 [7]. Table 1 shows prediction accuracies achieved by ExonScan[29], GenScan[20] and SpliceScan II in context of the gene fragments annotated in aberrant splicing databases [see Additional File 1]. For a prediction to be scored as correct a tool should predict the exonic boundary change the way it is annotated in the databases, i.e. the original exonic boundary and an aberrant boundary resulting from a mutation. In case of mutation creating a cryptic exon, appearance of both 3' and 5' boundaries of a cryptic exon have to be predicted correct. We compared only the methods that predict a gene structure in terms of exons, i.e. predicting which particular exon isoform is preferentially used in as result of mutation.

**Table 1:** Tools accuracy predicting the aberrant splicing events.

Prediction method	Databases					
	
	**DBASS5 **[7]			**DBASS3 **[8]		
	**Correct**	**Incorrect**	**Accuracy**	**Correct**	**Incorrect**	**Accuracy**

ExonScan[29]	42	320	11.6%	8	117	6.4%

GenScan[20]	52	310	14.36%	21	104	16.8%

SpliceScan II	100	262	27.62%	40	85	32%

Interpretation of counts reported in the table could be found in [see Additional File 1].

Our tool was twice as accurate compared to other top performing methods for gene splicing prediction, such as GenScan[20]. This result clearly demonstrates the performance improvement on gene fragments containing aberrant splicing isoforms when a method relies on splicing factors and signals rather than protein coding potential. The other ExonScan[29] method was not able to predict many aberrant splicing isoforms mainly because of the limited sensitivity, as discussed in the following subsection.

### SpliceScan II splicing prediction accuracy

**Figure 2:**
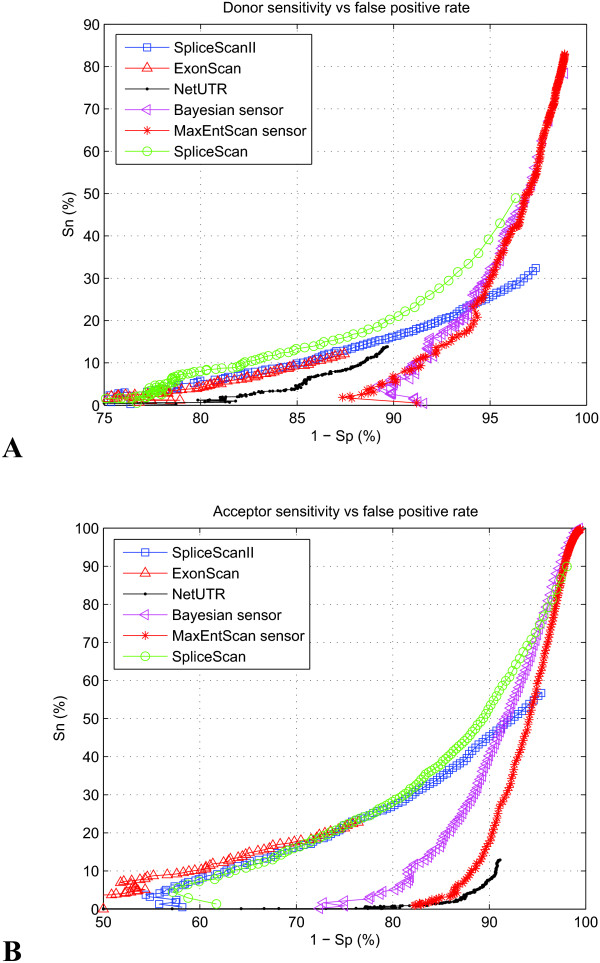
**Sensitivity vs. False positive rate trajectories for various tools**. The performance of Bayesian and Maximum entropy SS sensors is compared with the performance of tools specifically built to predict the splicing pattern independent of protein coding context features. (A) Trajectories for 5'SS (B) Trajectories for 3'SS.

**Figure 3:**
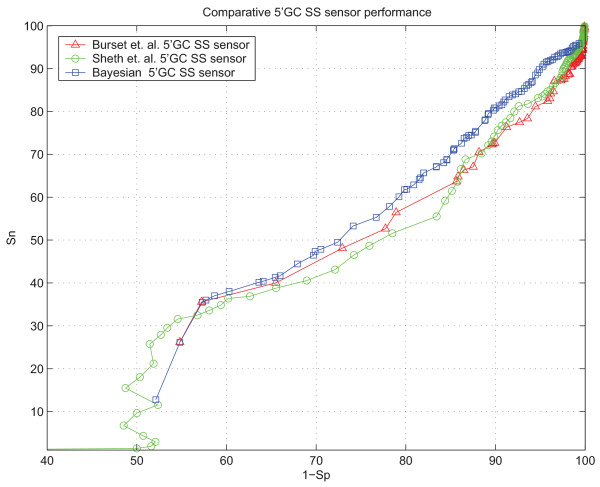
**5'GC SS Bayesian sensor performance compared with two existing 5'GC SS sensors **[12,15]**based on weight matrices**.

We estimated the performance of various *ab initio *splicing prediction methods with our web-based testing framework [34] using the test set [see Subsection *Constructing the test set*] as a benchmark. The comparative performance of the SpliceScan II is shown in Figure 2. The comparative performance of the 5'GC SS sensor on the set of gene structures containing 1,320 5'GC SSs [see Subsection *Learning the model*] is shown in Figure 3. In these experiments Sensitivity (*Sn*) and Specificity (*Sp*) were calculated according to the formulas

where *TE *is the number of accurately predicted exon boundaries, *AE *is the number of annotated exon boundaries in the test set and *PE *is the number of predicted exon boundaries.

The winning tool should be both sensitive and specific when predicting exonic boundaries for various thresholds. Our tool appeared to be twice as sensitive compared to other similar NetUTR[24] and ExonScan[29] methods (although at expense of much lower specificity), which would allow scoring roughly twice as many exonic isoforms. It has lesser sensitivity than the previously constructed SpliceScan[23] method, but the objective of two methods is different. Our new tool has the main focus to predict how certain internal exonic isoforms get activated, rather than assigning probabilistic scores to all putative SSs the way SpliceScan[23] and NetUTR[24] do.

Characteristics of tools shown in Figure 2 emphasize on comparative aspect of their performance, and do not necessarily reflect the prediction quality in practical cases. Intronic regions in our test set are long, which negatively affects sensitivity vs. false positive rates (the majority of false positive exons is predicted within introns). It has also been a split-sample test design for our tool, where we specifically removed the extended exons associated with the test set from the learning set, which has slightly detrimental effect on the SpliceScan II performance. The mutations causing aberrant splicing events, as annotated in DBASS databases [7,8], are normally located close to annotated exons, therefore in real experiments area of focus would normally be shifted to an annotated exon and surrounding context, where performance of our and other methods would certainly be higher than shown in Figure 2.

The 5'GC SS sensor outperforms the existing sensor designs based on weight matrices [12,15] for sensitivity values higher than 35%. The initial artifact in the trajectory below 35% sensitivity could be explained by the fact that 34.15% of 5'GC SSs are CAG**GC**AAGT and AAG**GC**AAGT, on which our sensor returns two predefined normalized scores of 0.914 and 0.744, correspondingly.

### Predicting variations in splicing induced by disease associated SNPs

We have predicted number of changes in gene splicing patterns induced by the polymorphic variations previously associated with predisposition to the breast cancer and Alzheimer's as shown in [see Additional File 2 Tables S1 and S2]. Some of the predicted changes are quite dramatic, but in general SNPs cause milder effect compared to the effect of mutations [see Section *Predicting aberrant splicing isoforms*] where annotated SSs routinely disappear or mutations create new cryptic exons. Number of polymorphic variants, potentially alternating composition of gene isoforms, was predicted for the disease associated and randomly selected groups of control SNPs are shown in Table 2.

**Table 2:** Number of predicted splicing events induced by the same number of disease associated and control SNPs randomly selected from the loci of 238 genes linked to ASD.

Type of event	997 SNPs			539 SNPs		
	
	Alzheimer's associated	Control	Ratio	Breast cancer associated	Control	Ratio
Predicted exon corresponding to an annotated exon disappears	0	2	0	0	0	-

Predicted exon corresponding to an annotated exon changes a score	43	12	3.58	11	2	5.5

Predicted exon sharing a SS with an annotated exon changes a score	242	78	3.10	59	29	2.03

Predicted exon sharing a SS with an annotated exon disappears	23	4	5.75	6	1	6.00

New predicted cryptic exon is created sharing a SS with with an annotated exon	26	9	2.89	5	1	5.00

Predicted exon disappears	50	49	1.02	30	17	1.76

New predicted cryptic exon is created	50	46	1.08	24	25	0.96

Comparison is made in context of known annotated reference exons. Not all the originally available SNPs associated with a disorder mapped to loci of protein coding genes, therefore number of SNPs reported here is lower than originally obtained [see Subsection *Constructing the test set*].

Here we tried to rank the polymorphic variations according to their possible destabilizing effect on splicing. We reported [see Additional File 2 Tables S1 and S2] polymorphic variations that change annotated exon score more than 2%, which according to [41] could cause increased exon skipping or retention compared to the reference exon. According to Table 2 number of such events induced by the disease associated SNPs is at least 3.5 times higher compared to control SNP groups, which suggests active role of the disease associated SNPs in modulation of predicted exonic strengths. These variations could indicate consequently different splicing commitment patterns for the affected exonic isoforms. Another class of events is the score change for the exons overlapping with the annotated exon, which according to [7,8,41] could disrupt mRNA inclusion patterns for alternatively used exon isoforms sharing an annotated boundary. According to Table 2 number of such events induced by the disease associated SNPs is also significantly increased compared to control SNP groups, which suggests destabilizing role for many of such changes listed in [see Additional File 2 Tables S1 and S2]. Similar splicing destabilizing effect could be achieved by simply removing or creating additional exon isoforms sharing a SS with an annotated exon and the number of such predicted events induced by the disease associated SNPs is also substantially increased compared to controls. On the contrary, the number of polymorphic variations associated with creation of new cryptic SSs or pseudo exon deletion is approximately the same for the disease associated and control group of SNPs, which suggests insignificant effect on splicing for these classes of events.

## Conclusions

Using the set of previously predicted cis-acting elements we were able to construct a splicing simulator capable of predicting exon score changes induced by mutations and polymorphic variants thus elucidating possible mechanism behind such variants leading to disorders caused by aberrant splicing.

Our tool performs favorably, compared to other splicing prediction methods, in context of genes linked to ASD. SpliceScan II provided more accurate prediction of aberrant splicing events, as documented in DBASS5 [7] and DBASS3 [8], compared to existing methods. Although the performance of our tool predicting the effect of mutations triggering an aberrant splicing is high compared to other methods, it could not be used as a general *ab initio *gene structural annotation method since the number of false positive predicted exons is high, as could be seen in Figure 2, though the fraction of reported false positives is comparable to what reported by other similar methods. Therefore, the most informative use of our method would evolve screening of polymorphic variants for possible splicing alternations in the context of known reference human gene structures. To accomplish this task we have created companion Autism Candidate Gene Map (ACGMAP) database http://www.meddean.luc.edu/node/375 that contains such structures and known alternative splicing variants for candidate ASD genes.

**Figure 4:**
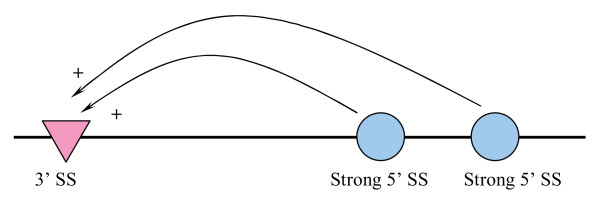
**Hypothetical situation of scoring putative 3'SS with **SpliceScan**method, where both strong 5'SSs located downstream positively affect the confidence of 3'SS**.

The reason the SpliceScan II is less specific (especially for higher sensitivity values) than previous SpliceScan tool [23] is in the nature of classification problem we address with new method. As could be seen in Figure 4, the SpliceScan uses simple probabilistic model of scoring putative 3' SS, where confidence of the putative 3' SS raises since two strong complement 5'SSs are located downstream. However, according to [7] 3'SS would unlikely to form exon with any of the 5' SSs located downstream in such a way since physiologically feasible 5'SS normally avoid strong competitors nearby. Indeed, SpliceScan scoring for both putative 5'SSs would be mediocre due to a conflict associated with their closeness. However, this observation does not help to predict which exonic isoform would be activated. To resolve this logical difficulty SpliceScan II makes all possible pairs of putative 3'SS and 5'SS located no further than 400 nt apart to predict possible SSs utilization. The number of pseudo exons formed this way outnumber the real exons by at least on order of magnitude [18], which turns in a harder classification task than simple SSs classification. For the weak SSs the number of putative exons to be classified is in excess of the number of weak SSs flanking them, which translates to a lower specificity compared to simpler SSs scoring methods.

Among the SNPs listed in dbSNP http://www.ncbi.nlm.nih.gov/projects/SNP/ as located within a gene locus, extended with 2 kb upstream and downstream flanks, only 1% are non synonymous variants [42]. The rarity of such SNPs suggested search for other causative variants affecting protein function through alternations in gene regulation, where disruption in splicing regulation seems a natural choice. Here we conducted search for causative alternations under realistic assumption that not all the SNPs associated with a disease are causative; chances are high that these SNPs were linked with causative variants located at the same haplotype. Moreover, associated variants may have different mechanism of compromising genome integrity such as creating missense/nonsense variants or affecting gene transcription regulatory elements. Nevertheless, we have been able to establish a number of potentially disease-causing splicing alternations.

Detected potentially detrimental exon score changes for disease associated SNPs are generally milder compared to the predicted changes associated with mutations annotated in DBASS databases [7,8]. We did not predict any disease associated SNPs causing an annotated exon to disappear, an event that would most likely have highly detrimental consequences. Comparison to the predicted changes associated with the same sized control groups of randomly selected SNPs indicated that disease-causing SNPs have pronounced excess in the number of detected potentially splice-disrupting variants. Careful examination of factors contributing to an exon score variation could lead to a plausible explanation of causative mechanisms behind the disease associated SNPs.

The SpliceScan II is available online at http://splicescan2.lumc.edu/. The 5'GC SS sensor and the standalone SpliceScan II program could be found at http://www.wyomingbioinformatics.org/~achurban/.

## Methods

### Sequence data collection and processing

A set of 2,333,379 *Tetrapoda *exons extended with 205 nt flanks from adjacent introns has been obtained as previously described in [40]. Pseudoexons, which were defined here as regions located between decoy 3' and 5' SSs, were extracted from intronic sequences flanked by two homology-based predicted exons in data set of human and mouse gene structures as described in [23]. The decoy 3' and 5' SSs were predicted by the Bayesian SS sensor [23]. The first and last 150 nt in every intronic sequence were excluded to avoid statistical biases associated with exon proximal ISEs/ISSs [43]. The sum of decoy 3' and 5' Bayesian SS sensor scores had to exceed 0.05, where the score for each signal was on a continuous 0 to 1 scale. The pseudoexon lengths were chosen to be longer than 5 nt and less than 400 nt, where 99% of authentic internal exons reside in this length range [41,44]. Flanking intronic regions of 205 nt were required on both sides of pseudo exons to estimate if any elements are associated with pseudo SSs. Pseudo exons were also checked for uniqueness and were discarded if either flanking regions of a pseudo exon or surrounding intronic fragments were identical to those previously processed.

Through the literature search we have collected the test set of 238 human genes previously linked to ASD [see Additional File 3] as a sample representative collection of important human genomic regions with potential implication in medical practice. We excluded all the extended exons corresponding to ASD genes from the learning set of SpliceScan II tool for the purposes of split-sample performance testing. We constructed a test set of pre-mRNA sequences for ASD genes along with the corresponding gene structural annotation. The set contains 4,650 known canonical 5' and 3' SS pairs flanking the internal exons that need to be predicted by various methods.

### Learning the model

**Figure 5:**
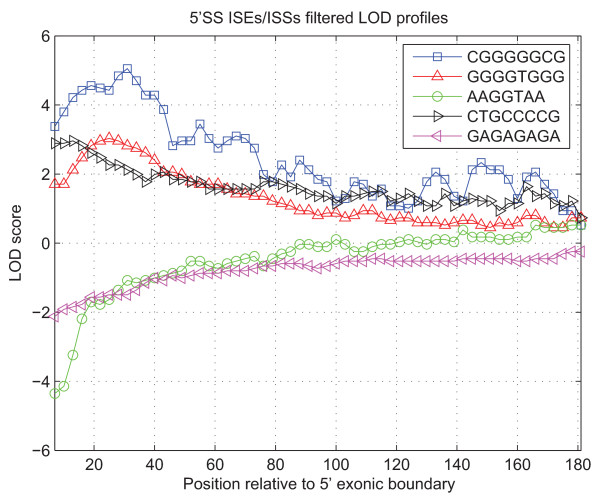
**An example LOD profiles for various 5'SS ISEs/ISSs signals in vicinity of a weak 5'SS (with discrete score 1 out of 3 possible)**. Signal AAGGTAA is a core part of a strong canonical 5'SS and therefore is substantially depleted in vicinity of true exonic boundaries as potential competitor. The distinctive bell-shaped LOD profiles for GGGGTGGG and CGGGGGCG are from the well studied poly-G family of ISEs [31], known to form quadruplex structures [57].

The LOD curves were constructed for the enhancers/silencers, previously reported in [40] [see Additional File 4], associated with the splicing signals of various strengths, an example of such dependencies could be seen in Figure 5. We followed the assumption that the weak splicing signals are more likely to be supported by the enhancing elements [30] and avoid silencers. In order to find LOD characteristic we calculate , where the quantity *Prob*(*D*|*H*) is called the likelihood of the data D (in our case ISEs, ISSs, ESEs, ESSs and competing SSs) under hypothesis *H*, *Prob*(*D*^*(i)*^|*H*_*SS*_) is a signal likelihood at location *i *next to a SS and *Prob*(*D*^*(i)*^|*H*_-*SS*_) is a signal likelihood at location *i *next to a splice-like signal.

**Figure 6:**
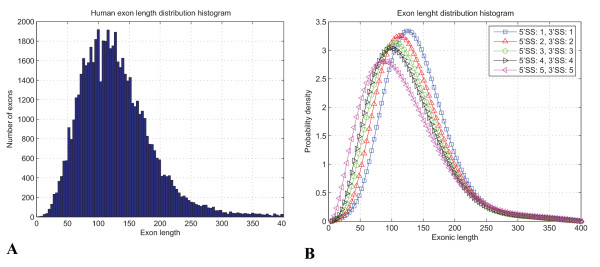
**Exonic length distribution depends on strength of flanking SSs**. We used substantial correlation of exonic sizes and the strength of SSs to explain how certain events change (compromise) the pattern of splicing, where 3' and 5' SSs strengths are in the discrete range from 1 to 5 as measured by the Bayesian SS sensor [23]. (A) Histogram of the internal exon length distribution. (B) The exonic length distribution histograms were interpolated with the mixture of Beta distributions fit with the Expectation Maximization (EM) as discussed in [23].

Exon definition score is found through combining of the 5' and 3' SSs strengths predicted by the Bayesian SS sensor [23] converted to LOD score, the LOD score associated with the exonic length for given SSs strength, the LOD scores associated with the presence of the strong splicing competitor signals in vicinity or inside of an exon defined and LOD scores associated with the enhancers/silencers. Steric constrains and geometry of the molecular interactions dictate the optimal exonic length distribution [1,45] where stronger SSs could sustain tighter packing of the splicing factors and therefore such exons are shorter as could be seen in Figure 6. The LOD score for an exon of length *Size*_*exon *_flanked by SSs of certain strength *Strength*_5'*SS *_and *Strength*_3'*SS *_are measured on a discrete scale from 1 to 5 by Bayesian SS sensor [23]) is calculated as , where *PDF*_*exon*_, (*Strength*_5'*SS*_, *Strength*_3'*SS *_,*Size*_*exon*_) is the Probability Density Function (PDF) of mixture of beta distributions interpolating the exon histogram as shown in Figure 6 and *PDF*_*uniform *_= 1 is the PDF of uniform distribution associated with the length of a pseudo exon. SS classification in our system follows Bayesian rule in terms LOD [46](1)

The middle two terms in (1) are the LOD ratio associated with the posterior probability score returned by the Bayesian sensor [23] for the 3' and 5' SSs. The first term in the sum (1) takes into account the evidence provided by the enhancers/silencers and comes up with a valid posterior LOD ratio.

To resolve LOD score contribution from overlapping enhancing/silencing elements we have allocated two sorted lists containing elements; one with positive LOD scores and another with negative. We keep only the elements with the highest negative LOD scores if any overlaps exist among silencers and with the highest positive LOD score if any overlaps exist among the enhancers. Such strategy allows scoring the overlapping elements that are antipodes in their enhancing profiles [47]. This way we can also choose between the shorter and longer version of the predicted cis-acting elements sharing the common prefix, relying only on elements contributing the maximum absolute LOD score.

Our Bayesian 5'GC SS sensor has been constructed in a manner similar to the canonical Bayesian 5'SS sensor [23], which demonstrated the predictive performance superior to other SS sensor designs. To construct the sensor, first we have collected gene structures containing 1,320 5'GC SS from homology based annotations of human and mouse genomes (described in [23]) along with pre-mRNA frequencies of decoy 5'GC SSs. The entire learning set of 23 *Tetrapoda *organisms confirmed 19,059 non-canonical 5'GC SSs. Since other organisms presented in UCSC multiple genome alignments, beyond human and mouse, had poor genome annotations we amplified found decoy 5'GC SS scores by the factor of  to approximate decoy counts for the 23 tetrapods. Table 3 shows first 40 top-scoring 5'GC SS posterior probabilities calculated according to the formula

**Table 3:** Frequencies of oligonucleotides playing role of 5'GC SSs versus frequency of decoy 5'GC SS-like oligonucleotides in pre-mRNA sequences recordered for tetrapoda organisms.

Signal	Counted as true SS in Vertebrates	Counted as decoy SS in Vertebrates	Bayesian posterior	Normalized
CAGGCAAGT	3263	36400	0.082	0.914

AAGGCAAGT	3246	41193	0.073	0.744

GAGGCAAGT	1898	35375	0.051	0.609

ACGGCAAGT	143	4519	0.031	0.555

AAGGCGAGT	199	6570	0.029	0.546

CAGGCGAGT	231	8750	0.026	0.535

ATGGCAAGT	580	30928	0.018	0.514

TCGGCAAGT	64	3971	0.016	0.497

GAGGCGAGT	141	9746	0.014	0.491

CCGGCAAGT	62	4534	0.013	0.486

AAGGCAAGC	415	36154	0.011	0.474

TAGGCAAGT	398	34927	0.011	0.452

CGGGCAAGT	64	5963	0.011	0.440

AAGGCACGT	92	10006	0.009	0.436

CTGGCAAGT	304	34884	0.009	0.426

AAGGCAAGG	475	54694	0.009	0.405

AAGGCAAGA	517	61104	0.008	0.379

CAGGCAAGA	365	55964	0.006	0.356

CAGGCAAGG	351	55387	0.006	0.337

CAGGCAAGC	275	44919	0.006	0.321

GAGGCACGT	51	9371	0.005	0.312

AGGGCAAGT	175	32213	0.005	0.306

TTGGCAAGT	188	36674	0.005	0.297

AACGCAAGT	19	3870	0.005	0.291

GCGGCAAGT	20	4303	0.005	0.290

GAGGCAAGC	166	37252	0.004	0.285

CGCGCAAGC	5	1155	0.004	0.281

AAGGCAGGT	292	75658	0.004	0.273

CCGGCACGT	9	2455	0.004	0.265

CAGGCACGT	122	35158	0.003	0.262

CCGGCGAGT	7	2036	0.003	0.258

GAGGCATGT	114	33194	0.003	0.255

TCGGCGAGT	4	1184	0.003	0.252

CAGGCAGGT	271	81896	0.003	0.245

TAGGCGAGT	12	3855	0.003	0.238

ATGGCGAGT	18	5790	0.003	0.237

AAAGCAAGT	209	67530	0.003	0.231

ACGGCACGT	5	1617	0.003	0.225

AAGGCATGT	174	60195	0.003	0.220

AAGGCGCGT	5	1733	0.003	0.216

Here prior probability for 5'GC SS is *P *(*SS*) = 5.16 × 10^-5^.

where *P*(SS) - prior probability of an oligonucleotide to be 5'GC SS, *P*(-*SS*) - prior probability of an oligonucleotide to be donor-like signal, *P*(*oligo*|*SS*) - likelihood of oligonucleotide in case of 5'GC SS, *P*(*oligo*|-*SS*) - likelihood of oligonucleotide in case of 5'GC SS-like signal.

Since the 5'GC SSs are recognized by the standard U2 spliceosome [1] and are commonly interchangeable with the canonical 5' SSs [36], it is reasonable to assume they share the common context. For that reason the splicing signals predicted by the newly constructed Bayesian 5'GC SS sensor were placed in the same probabilistic context of the normal 5'SSs, except for the different initial LOD characteristic of the 5'GC SS sensor and additional normalization histogram to specifically normalized score for 5'GC SSs flanked exons.

### Constructing the test set

We wanted to estimate a potential implication of disease associated Single Nucleotide Polymorphisms (SNPs) on splicing, since many such variants emerge from recently conducted association studies. A mechanism by which these variations influence a disorder predisposition remains elusive in many cases. We have identified 1,481 SNPs that have been previously associated with Alzheimer's available through AlzGene database http://www.alzforum.org/res/com/gen/alzgene/default.asp and the literature sources cited at Alzheimer research forum http://www.alzforum.org/ and the 716 SNPs that have been previously associated with the breast cancer [48-53] [see Additional File 3]. We batch downloaded the sequences for the SNPs from the dbSNP http://www.ncbi.nlm.nih.gov/projects/SNP/, BLASTN [54] aligned these sequences against Ensembl genomic contig sequences obtained from EBI Alternative Splicing Database project http://www.ebi.ac.uk/asd/altsplice/humrel3.html, processed the results and mapped the location of SNPs to the genomic contig sequences. For the same genomic sequences we predicted the gene structures with BLAT [55] using the RefSeq mRNA sequences ftp://ftp.ncbi.nih.gov/refseq/H_sapiens/mRNA_Prot. We synchronized SpliceScan II*ab initio *splicing predictions with the homology-based annotated exons and reported changes induced by the polymorphic variations. Sets of control SNPs were randomly selected from loci of 238 genes linked to ASD [see Additional File 3].

## Authors' contributions

AC designed and implemented the SpliceScan II program and tested the application. CH collected the set of the disease associated SNPs, provided many valuable suggestions steering the project and was the head of the lab where the work was done. IV provided necessary expertise to formulate the study objectives, designed the DBASS5 and DBASS3 databases used in our experiments and extensively edited the manuscript. All authors read and approved the final manuscript.

## Supplementary Material

Additional file 1 **Report and analysis of mutations causing aberrant splicing events reported in DBASS5 **[7]**and DBASS3 **[8]**databases**. Prediction accuracy for aberrant splicing events triggered by mutations is reported for SpliceScan II, ExonScan[29] and GenScan[20] tools. Click here for file

Additional file 2 **Predicted splicing variations caused by SNPs previously associated with Alzheimer's and the breast cancer**. SNPs previously associated with Alzheimer's and breast cancer predicted to change the pattern of splicing. Click here for file

Additional file 3 **Genes linked to ASD, SNPs previously associated with Alzheimer's and breast cancer and control SNPs randomly picked from loci of genes associated with ASD**. SNPs previously associated with Alzheimer's and breast cancer and genes linked to ASD were collected through literature search. Click here for file

Additional file 4 **Splicing regulatory elements reported in **[40]**and their statistical significance**. Repertoire of exonic and intronic splicing enhancer/silencer elements used in building of SpliceScan II tool. Click here for file
