# Supplementary material for: A method of predicting changes in human gene splicing induced by genetic variants in context of cis-acting elements
Source: BMC Bioinformatics. 2010 Jan 12;11:22. doi: 10.1186/1471-2105-11-22 (PMC3098058; doi:10.1186/1471-2105-11-22)
Supplement: Additional file 2 — Predicted splicing variations caused by SNPs previously associated with Alzheimer's and the breast cancer. SNPs previously associated with Alzheimer's and breast cancer predicted to change the pattern of splicing. [file 1471-2105-11-22-S2.DOC]

# Supporting materials

**Table 1**. SNPs previously associated with Alzheimer’s predicted to change the pattern of splicing.

| **SNP ID** | **Gene** | **Sequence ID** | **Affected exon coordinates** | **Associated score change** |
| --- | --- | --- | --- | --- |
| **Score of an annotated exon changes** | | | | |
| rs7724759 | CAST | NM_001750.5 | 1066-1104 | 0.65→0.22 |
| rs1800454 | TAP2 | NM_000544.3 | 1068-1265 | 0.54→0.7 |
| rs5406 | SLC2A2 | NM_000340.1 | 1085-1272 | 0.53→0.51 |
| rs561945 | ACAD8 | NM_014384.2 | 1154-1256 | 0.87→0.77 |
| rs490460 | BACE1 | NM_012104.3 | 1167-1301 | 0.84→0.66 |
| rs2252576 | BACE2 | NM_012105.3 | 1211-1345 | 0.38→0.4 |
| rs1800774 | CETP | NM_000078.2 | 1272-1305 | 0.25→0.27 |
| rs505045 | RNF214 | NM_001077239.1 | 1283-1381 | 0.67→0.7 |
| rs7528638 | NCSTN | NM_015331.2 | 1304-1476 | 0.59→0.45 |
| rs283696 | RXRG | NM_006917.3 | 1439-1544 | 0.68→0.72 |
| rs5498 | ICAM1 | NM_000201.2 | 1500-1745 | 0.9→0.95 |
| rs2290841 | ADAM12 | NM_003474.3 | 1506-1683 | 0.85→0.88 |
| rs2250889 | MMP9 | NM_004994.2 | 1630-1769 | 0.89→0.94 |
| rs328 | LPL | NM_000237.2 | 1693-1797 | 0.77→0.71 |
| rs2279090 | ADAM12 | NM_003474.3 | 1774-1969 | 0.73→0.8 |
| rs688 | LDLR | NM_000527.3 | 1874-2013 | 0.46→0.27 |
| rs2290677 | CAST | NM_001750.5 | 1897-1947 | 0.42→0.44 |
| rs17577 | MMP9 | NM_004994.2 | 1921-2024 | 0.8→0.96 |
| rs2290677 | CAST | NM_001750.5 | 1948-2019 | 0.38→0.4 |
| rs745975 | HNF4A | NM_000457.3 | 205-379 | 0.68→0.76 |
| rs4746147 | SEC24C | NM_004922.2 | 2190-2360 | 0.33→0.29 |
| rs3745833 | GALP | NM_033106.2 | 219-299 | 0.95→0.81 |
| rs758995 | ADCYAP1R1 | NM_001118.3 | 275-380 | 0.96→0.88 |
| rs3742376 | CYP46A1 | NM_006668.1 | 283-356 | 0.07→0.03 |
| rs2229765 | IGF1R | NM_000875.3 | 3007-3236 | 0.61→0.64 |
| rs2072374 | NCAPD2 | NM_014865.3 | 3346-3523 | 0.37→0.39 |
| rs1800194 | LRP1 | NM_002332.2 | 3813-4013 | 0.54→0.28 |
| rs2228591 | NCOA2 | NM_006540.2 | 3844-4078 | 0.18→0.1 |
| rs700518 | CYP19A1 | NM_031226.2 | 397-547 | 0.46→0.49 |
| rs3826803 | DNM2 | NM_001005360.1 | 400-549 | 0.69→0.74 |
| rs3743074 | CHRNA3 | NM_000743.2 | 454-563 | 0.61→0.2 |
| rs1801046 | C1R | NM_001733.4 | 484-630 | 0.79→0.6 |
| rs4680 | COMT | NM_000754.2 | 492-685 | 0.75→0.73 |
| rs1138272 | GSTP1 | NM_000852.3 | 586-693 | 0.25→0.21 |
| rs1799782 | XRCC1 | NM_006297.2 | 610-721 | 0.54→0.35 |
| rs2456778 | CDC2 | NM_001786.2 | 619-782 | 0.09→0.12 |
| rs3212319 | CDC2 | NM_001786.2 | 619-782 | 0.09→0.33 |
| rs1800206 | PPARA | NM_005036.4 | 636-774 | 0.42→0.38 |
| rs2290604 | PPARGC1A | NM_013261.3 | 673-877 | 0.2→0.16 |
| rs3736235 | OLR1 | NM_002543.3 | 679-794 | 0.5→0.06 |
| rs607755 | RELN | NM_005045.2 | 737-815 | 0.85→0.89 |
| rs3212319 | CDC2 | NM_001786.2 | 783-924 | 0.8→0.87 |
| rs2285789 | SORCS2 | NM_020777.2 | 814-887 | 0.72→0.75 |
| **Score of an exon sharing a splice site with an annotated exon changes** | | | | |
| rs1003857 | RPS6KA2 | NM_021135.4 | 319-435 | 0.77→0.75 |
| rs1003857 | RPS6KA2 | NM_021135.4 | 319-435 | 0.08→0.06 |
| rs1012672 | LRP6 | NM_002336.2 | 3876-4112 | 0.21→0.24 |
| rs1042522 | TP53 | NM_001126114.1 | 272-293 | 0.35→0.59 |
| rs1042522 | TP53 | NM_001126114.1 | 294-572 | 0.36→0.6 |
| rs1042523 | PCK1 | NM_002591.3 | 570-773 | 0.63→0.68 |
| rs1045642 | ABCB1 | NM_000927.3 | 3701-3907 | 0.19→0.33 |
| rs1045642 | ABCB1 | NM_000927.3 | 3701-3907 | 0.08→0.16 |
| rs1045642 | ABCB1 | NM_000927.3 | 3701-3907 | 0.15→0.28 |
| rs1046117 | FOS | NM_005252.2 | 297-548 | 0.81→0.78 |
| rs1048099 | ABCC8 | NM_000352.3 | 275-416 | 0.02→0.06 |
| rs1048099 | ABCC8 | NM_000352.3 | 275-416 | 0.16→0.35 |
| rs1048099 | ABCC8 | NM_000352.3 | 275-416 | 0.28→0.52 |
| rs1048099 | ABCC8 | NM_000352.3 | 275-416 | 0.44→0.64 |
| rs1049125 | PNLIPRP1 | NM_006229.2 | 1359-1480 | 0.15→0.29 |
| rs1049125 | PNLIPRP1 | NM_006229.2 | 1359-1480 | 0.18→0.34 |
| rs1049125 | PNLIPRP1 | NM_006229.2 | 1359-1480 | 0.29→0.48 |
| rs1049125 | PNLIPRP1 | NM_006229.2 | 1359-1480 | 0.43→0.65 |
| rs11280 | C6orf130 | NM_145063.2 | 529-1174 | 0.38→0.2 |
| rs1142530 | NDUFS7 | NM_024407.4 | 166-271 | 0.03→0.15 |
| rs12239747 | NCSTN | NM_015331.2 | 707-857 | 0.61→0.68 |
| rs1278278 | ADAM12 | NM_003474.3 | 1774-1969 | 0.08→0.03 |
| rs1278281 | ADAM12 | NM_003474.3 | 1684-1773 | 0.03→0.06 |
| rs1278281 | ADAM12 | NM_003474.3 | 1684-1773 | 0.01→0.04 |
| rs1332018 | GSTM3 | NM_000849.3 | 1-179 | 0.53→0.33 |
| rs1348316 | PRKAB2 | NM_005399.3 | 123-301 | 0.29→0.36 |
| rs1348316 | PRKAB2 | NM_005399.3 | 123-301 | 0.46→0.54 |
| rs1348316 | PRKAB2 | NM_005399.3 | 123-301 | 0.12→0.16 |
| rs1348316 | PRKAB2 | NM_005399.3 | 123-301 | 0.48→0.65 |
| rs1348316 | PRKAB2 | NM_005399.3 | 123-301 | 0.3→0.38 |
| rs1348316 | PRKAB2 | NM_005399.3 | 123-301 | 0.4→0.48 |
| rs1554948 | TNK1 | NM_003985.3 | 70-323 | 0.23→0.2 |
| rs1594 | CFLAR | NM_003879.4 | 1246-1756 | 0.14→0.11 |
| rs1594 | CFLAR | NM_003879.4 | 1246-1756 | 0.07→0.05 |
| rs1594 | CFLAR | NM_003879.4 | 1246-1756 | 0.08→0.06 |
| rs1594 | CFLAR | NM_003879.4 | 1246-1756 | 0.19→0.15 |
| rs1594 | CFLAR | NM_003879.4 | 1246-1756 | 0.29→0.23 |
| rs1594 | CFLAR | NM_003879.4 | 1246-1756 | 0.22→0.31 |
| rs16139 | NPY | NM_000905.2 | 87-274 | 0.03→0.09 |
| rs17108177 | LRP8 | NM_004631.3 | 1395-1569 | 0.18→0.09 |
| rs171649 | PIK3R1 | NM_181523.1 | 470-544 | 0.26→0.24 |
| rs171649 | PIK3R1 | NM_181523.1 | 470-544 | 0.55→0.58 |
| rs17577 | MMP9 | NM_004994.2 | 1921-2024 | 0.29→0.73 |
| rs17577 | MMP9 | NM_004994.2 | 1921-2024 | 0.29→0.73 |
| rs17577 | MMP9 | NM_004994.2 | 1921-2024 | 0.13→0.44 |
| rs17577 | MMP9 | NM_004994.2 | 1921-2024 | 0.01→0.08 |
| rs17577 | MMP9 | NM_004994.2 | 1921-2024 | 0.21→0.18 |
| rs1799782 | XRCC1 | NM_006297.2 | 610-721 | 0.28→0.15 |
| rs1799782 | XRCC1 | NM_006297.2 | 610-721 | 0.88→0.78 |
| rs1799782 | XRCC1 | NM_006297.2 | 610-721 | 0.88→0.78 |
| rs1799782 | XRCC1 | NM_006297.2 | 610-721 | 0.13→0.06 |
| rs1799782 | XRCC1 | NM_006297.2 | 610-721 | 0.26→0.13 |
| rs1799782 | XRCC1 | NM_006297.2 | 610-721 | 0.94→0.89 |
| rs1799782 | XRCC1 | NM_006297.2 | 610-721 | 0.83→0.69 |
| rs1799854 | ABCC8 | NM_000352.3 | 2243-2348 | 0.1→0.06 |
| rs1799854 | ABCC8 | NM_000352.3 | 2243-2348 | 0.14→0.09 |
| rs1799854 | ABCC8 | NM_000352.3 | 2243-2348 | 0.15→0.1 |
| rs1799854 | ABCC8 | NM_000352.3 | 2243-2348 | 0.68→0.58 |
| rs1799854 | ABCC8 | NM_000352.3 | 2243-2348 | 0.92→0.85 |
| rs1800194 | LRP1 | NM_002332.2 | 3813-4013 | 0.15→0.05 |
| rs1800194 | LRP1 | NM_002332.2 | 3813-4013 | 0.11→0.04 |
| rs1800194 | LRP1 | NM_002332.2 | 3813-4013 | 0.05→0.02 |
| rs1800194 | LRP1 | NM_002332.2 | 3813-4013 | 0.45→0.23 |
| rs1800206 | PPARA | NM_005036.4 | 636-774 | 0.27→0.24 |
| rs1800454 | TAP2 | NM_000544.3 | 1068-1265 | 0.07→0.14 |
| rs1800454 | TAP2 | NM_000544.3 | 1068-1265 | 0.04→0.08 |
| rs1800454 | TAP2 | NM_000544.3 | 1068-1265 | 0.05→0.09 |
| rs1800454 | TAP2 | NM_000544.3 | 1068-1265 | 0.03→0.06 |
| rs1800684 | AGER | NM_001136.3 | 1-76 | 0.08→0.16 |
| rs1800684 | AGER | NM_001136.3 | 1-76 | 0.22→0.36 |
| rs1800684 | AGER | NM_001136.3 | 1-76 | 0.03→0.07 |
| rs1801018 | BCL2 | NM_000633.2 | 208-1078 | 0.21→0.25 |
| rs1801046 | C1R | NM_001733.4 | 484-630 | 0.19→0.11 |
| rs1801311 | NDUFA6 | NM_002490.3 | 1-279 | 0.56→0.28 |
| rs196295 | BAG3 | NM_004281.3 | 1216-2569 | 0.24→0.04 |
| rs2010457 | PIN1 | NM_006221.2 | 294-404 | 0.09→0.19 |
| rs2010457 | PIN1 | NM_006221.2 | 294-404 | 0.29→0.51 |
| rs2010457 | PIN1 | NM_006221.2 | 294-404 | 0.29→0.16 |
| rs2016520 | PPARD | NM_006238.3 | 209-439 | 0.25→0.22 |
| rs2018621 | POMT1 | NM_007171.3 | 808-967 | 0.13→0.09 |
| rs2018621 | POMT1 | NM_007171.3 | 808-967 | 0.16→0.05 |
| rs2018621 | POMT1 | NM_007171.3 | 808-967 | 0.36→0.29 |
| rs2018621 | POMT1 | NM_007171.3 | 808-967 | 0.39→0.36 |
| rs2018621 | POMT1 | NM_007171.3 | 968-1123 | 0.59→0.07 |
| rs2059806 | INSR | NM_000208.2 | 1720-1970 | 0.61→0.47 |
| rs2074308 | ABCC8 | NM_000352.3 | 1798-1943 | 0.01→0.04 |
| rs2228591 | NCOA2 | NM_006540.2 | 3844-4078 | 0.47→0.42 |
| rs2229765 | IGF1R | NM_000875.3 | 3007-3236 | 0.21→0.23 |
| rs2229765 | IGF1R | NM_000875.3 | 3007-3236 | 0.21→0.23 |
| rs2234252 | TREM2 | NM_018965.2 | 143-493 | 0.06→0.03 |
| rs2250889 | MMP9 | NM_004994.2 | 1630-1769 | 0.09→0.19 |
| rs2252576 | BACE2 | NM_012105.3 | 1211-1345 | 0.17→0.2 |
| rs2252576 | BACE2 | NM_012105.3 | 1211-1345 | 0.21→0.23 |
| rs226380 | A2M | NM_000014.4 | 1-199 | 0.2→0.24 |
| rs226380 | A2M | NM_000014.4 | 1-199 | 0.04→0.07 |
| rs226380 | A2M | NM_000014.4 | 1-199 | 0.53→0.35 |
| rs2273502 | CHRNA4 | NM_000744.5 | 407-451 | 0.19→0.33 |
| rs2279090 | ADAM12 | NM_003474.3 | 1774-1969 | 0.26→0.35 |
| rs2279090 | ADAM12 | NM_003474.3 | 1774-1969 | 0.42→0.52 |
| rs2279090 | ADAM12 | NM_003474.3 | 1774-1969 | 0.79→0.85 |
| rs2279090 | ADAM12 | NM_003474.3 | 1774-1969 | 0.14→0.2 |
| rs2279090 | ADAM12 | NM_003474.3 | 1774-1969 | 0.37→0.46 |
| rs2279090 | ADAM12 | NM_003474.3 | 1774-1969 | 0.11→0.17 |
| rs2279090 | ADAM12 | NM_003474.3 | 1774-1969 | 0.22→0.3 |
| rs2279090 | ADAM12 | NM_003474.3 | 1774-1969 | 0.1→0.14 |
| rs2285781 | SORCS2 | NM_020777.2 | 2124-2252 | 0.44→0.41 |
| rs2285781 | SORCS2 | NM_020777.2 | 2124-2252 | 0.73→0.71 |
| rs2285781 | SORCS2 | NM_020777.2 | 2124-2252 | 0.26→0.24 |
| rs2285781 | SORCS2 | NM_020777.2 | 2124-2252 | 0.41→0.04 |
| rs2285789 | SORCS2 | NM_020777.2 | 814-887 | 0.79→0.81 |
| rs2290677 | CAST | NM_001750.5 | 1948-2019 | 0.21→0.23 |
| rs2290677 | CAST | NM_001750.5 | 1948-2019 | 0.21→0.23 |
| rs2290841 | ADAM12 | NM_003474.3 | 1506-1683 | 0.03→0.05 |
| rs2290841 | ADAM12 | NM_003474.3 | 1506-1683 | 0.21→0.24 |
| rs2292692 | ADAM12 | NM_003474.3 | 2079-2277 | 0.13→0.11 |
| rs2297235 | GSTO2 | NM_183239.1 | 398-662 | 0.84→0.87 |
| rs2304893 | PRKAB2 | NM_005399.3 | 887-5423 | 0.29→0.06 |
| rs2306325 | SEC24C | NM_004922.2 | 152-351 | 0.62→0.82 |
| rs2456777 | CDC2 | NM_001786.2 | 783-924 | 0.02→0.05 |
| rs2456778 | CDC2 | NM_001786.2 | 619-782 | 0.03→0.05 |
| rs283696 | RXRG | NM_006917.3 | 1439-1544 | 0.47→0.52 |
| rs2839158 | LSS | NM_002340.3 | 461-582 | 0.49→0.29 |
| rs289741 | CETP | NM_000078.2 | 1465-1717 | 0.11→0.21 |
| rs289741 | CETP | NM_000078.2 | 1465-1717 | 0.24→0.4 |
| rs289741 | CETP | NM_000078.2 | 1465-1717 | 0.55→0.71 |
| rs3118570 | RXRA | NM_002957.3 | 979-1111 | 0.17→0.07 |
| rs3118570 | RXRA | NM_002957.3 | 979-1111 | 0.2→0.1 |
| rs3212319 | CDC2 | NM_001786.2 | 619-782 | 0.11→0.06 |
| rs3212319 | CDC2 | NM_001786.2 | 619-782 | 0.03→0.16 |
| rs3212319 | CDC2 | NM_001786.2 | 783-924 | 0.05→0.01 |
| rs3212319 | CDC2 | NM_001786.2 | 783-924 | 0.03→0.06 |
| rs328 | LPL | NM_000237.2 | 1693-1797 | 0.1→0.07 |
| rs328 | LPL | NM_000237.2 | 1693-1797 | 0.17→0.13 |
| rs328 | LPL | NM_000237.2 | 1693-1797 | 0.26→0.2 |
| rs3730089 | PIK3R1 | NM_181523.1 | 959-1061 | 0.07→0.04 |
| rs3731865 | SLC11A1 | NM_000578.3 | 614-733 | 0.22→0.26 |
| rs3731865 | SLC11A1 | NM_000578.3 | 614-733 | 0.39→0.36 |
| rs3731865 | SLC11A1 | NM_000578.3 | 614-733 | 0.68→0.72 |
| rs3733359 | GC | NM_000583.2 | 1-211 | 0.24→0.11 |
| rs3733359 | GC | NM_000583.2 | 1-211 | 0.13→0.06 |
| rs3733359 | GC | NM_000583.2 | 1-211 | 0.38→0.3 |
| rs3736187 | MME | NM_007288.2 | 2057-2122 | 0.14→0.06 |
| rs3736235 | OLR1 | NM_002543.3 | 679-794 | 0.22→0.29 |
| rs3736235 | OLR1 | NM_002543.3 | 679-794 | 0.16→0.23 |
| rs3736235 | OLR1 | NM_002543.3 | 679-794 | 0.05→0.07 |
| rs3742376 | CYP46A1 | NM_006668.1 | 283-356 | 0.15→0.04 |
| rs3742376 | CYP46A1 | NM_006668.1 | 283-356 | 0.06→0.02 |
| rs3745833 | GALP | NM_033106.2 | 219-299 | 0.06→0.01 |
| rs3745833 | GALP | NM_033106.2 | 219-299 | 0.38→0.11 |
| rs3745833 | GALP | NM_033106.2 | 219-299 | 0.12→0.02 |
| rs3745833 | GALP | NM_033106.2 | 219-299 | 0.53→0.59 |
| rs3826803 | DNM2 | NM_001005360.1 | 400-549 | 0.3→0.25 |
| rs3826803 | DNM2 | NM_001005360.1 | 400-549 | 0.71→0.56 |
| rs3827020 | CHRNA4 | NM_000744.5 | 562-1936 | 0.88→0.92 |
| rs3827020 | CHRNA4 | NM_000744.5 | 562-1936 | 0.87→0.91 |
| rs3831458 | SNCA | NM_000345.2 | 168-209 | 0.16→0.07 |
| rs3831458 | SNCA | NM_000345.2 | 168-209 | 0.16→0.02 |
| rs4295 | ACE | NM_000789.2 | 440-533 | 0.48→0.5 |
| rs4311 | ACE | NM_000789.2 | 1510-1608 | 0.05→0.22 |
| rs4362 | ACE | NM_000789.2 | 3403-3525 | 0.15→0.06 |
| rs4363 | ACE | NM_000789.2 | 3714-4195 | 0.53→0.5 |
| rs4363 | ACE | NM_000789.2 | 3714-4195 | 0.53→0.5 |
| rs4680 | COMT | NM_000754.2 | 492-685 | 0.75→0.73 |
| rs4746147 | SEC24C | NM_004922.2 | 2190-2360 | 0.31→0.37 |
| rs4746147 | SEC24C | NM_004922.2 | 2190-2360 | 0.62→0.65 |
| rs4746147 | SEC24C | NM_004922.2 | 2190-2360 | 0.37→0.29 |
| rs4832 | ACAT2 | NM_005891.2 | 1155-1551 | 0.35→0.18 |
| rs490460 | BACE1 | NM_012104.3 | 1167-1301 | 0.08→0.01 |
| rs490460 | BACE1 | NM_012104.3 | 1167-1301 | 0.08→0.02 |
| rs490460 | BACE1 | NM_012104.3 | 1167-1301 | 0.06→0.02 |
| rs490460 | BACE1 | NM_012104.3 | 1167-1301 | 0.85→0.61 |
| rs490460 | BACE1 | NM_012104.3 | 1167-1301 | 0.27→0.42 |
| rs490460 | BACE1 | NM_012104.3 | 1167-1301 | 0.43→0.47 |
| rs490460 | BACE1 | NM_012104.3 | 1167-1301 | 0.38→0.16 |
| rs4947710 | GRB10 | NM_001001555.1 | 1630-1746 | 0.19→0.27 |
| rs4947710 | GRB10 | NM_001001555.1 | 1630-1746 | 0.06→0.1 |
| rs4947710 | GRB10 | NM_001001555.1 | 1630-1746 | 0.57→0.68 |
| rs4947710 | GRB10 | NM_001001555.1 | 1630-1746 | 0.5→0.6 |
| rs4947710 | GRB10 | NM_001001555.1 | 1630-1746 | 0.65→0.74 |
| rs4947710 | GRB10 | NM_001001555.1 | 1630-1746 | 0.43→0.54 |
| rs4998 | ADRB3 | NM_000025.1 | 1403-2644 | 0.08→0.1 |
| rs505058 | LMNA | NM_170707.2 | 1407-1629 | 0.22→0.25 |
| rs505058 | LMNA | NM_170707.2 | 1407-1629 | 0.29→0.32 |
| rs505058 | LMNA | NM_170707.2 | 1407-1629 | 0.51→0.55 |
| rs5104 | APOA4 | NM_000482.3 | 281-1460 | 0.7→0.27 |
| rs5174 | LRP8 | NM_004631.3 | 2996-4507 | 0.32→0.3 |
| rs5174 | LRP8 | NM_004631.3 | 2996-4507 | 0.37→0.35 |
| rs5174 | LRP8 | NM_004631.3 | 2996-4507 | 0.46→0.44 |
| rs5404 | SLC2A2 | NM_000340.1 | 806-921 | 0.48→0.51 |
| rs5443 | GNB3 | NM_002075.2 | 1105-1321 | 0.8→0.85 |
| rs5498 | ICAM1 | NM_000201.2 | 1500-1745 | 0.34→0.58 |
| rs5498 | ICAM1 | NM_000201.2 | 1500-1745 | 0.35→0.59 |
| rs5498 | ICAM1 | NM_000201.2 | 1500-1745 | 0.05→0.12 |
| rs5498 | ICAM1 | NM_000201.2 | 1500-1745 | 0.13→0.3 |
| rs5498 | ICAM1 | NM_000201.2 | 1500-1745 | 0.68→0.6 |
| rs5498 | ICAM1 | NM_000201.2 | 1500-1745 | 0.73→0.89 |
| rs5498 | ICAM1 | NM_000201.2 | 1500-1745 | 0.69→0.85 |
| rs5498 | ICAM1 | NM_000201.2 | 1500-1745 | 0.77→0.9 |
| rs5498 | ICAM1 | NM_000201.2 | 1500-1745 | 0.48→0.71 |
| rs556349 | SORL1 | NM_003105.4 | 2520-2651 | 0.26→0.33 |
| rs561945 | ACAD8 | NM_014384.2 | 1154-1256 | 0.25→0.15 |
| rs561945 | ACAD8 | NM_014384.2 | 1154-1256 | 0.3→0.18 |
| rs561945 | ACAD8 | NM_014384.2 | 1154-1256 | 0.07→0.04 |
| rs561945 | ACAD8 | NM_014384.2 | 1154-1256 | 0.06→0.03 |
| rs561945 | ACAD8 | NM_014384.2 | 1154-1256 | 0.12→0.06 |
| rs607755 | RELN | NM_005045.2 | 737-815 | 0.09→0.14 |
| rs607755 | RELN | NM_005045.2 | 737-815 | 0.07→0.11 |
| rs607755 | RELN | NM_005045.2 | 737-815 | 0.16→0.23 |
| rs607755 | RELN | NM_005045.2 | 737-815 | 0.05→0.07 |
| rs638405 | BACE1 | NM_012104.3 | 1167-1301 | 0.27→0.3 |
| rs688 | LDLR | NM_000527.3 | 1874-2013 | 0.08→0.03 |
| rs700518 | CYP19A1 | NM_031226.2 | 397-547 | 0.71→0.73 |
| rs745975 | HNF4A | NM_000457.3 | 205-379 | 0.49→0.62 |
| rs745975 | HNF4A | NM_000457.3 | 205-379 | 0.15→0.21 |
| rs745975 | HNF4A | NM_000457.3 | 205-379 | 0.05→0.16 |
| rs746868 | LTA | NM_000595.2 | 132-239 | 0.23→0.17 |
| rs7528638 | NCSTN | NM_015331.2 | 1304-1476 | 0.06→0.13 |
| rs7528638 | NCSTN | NM_015331.2 | 1304-1476 | 0.64→0.66 |
| rs758995 | ADCYAP1R1 | NM_001118.3 | 275-380 | 0.06→0.01 |
| rs758995 | ADCYAP1R1 | NM_001118.3 | 275-380 | 0.18→0.05 |
| rs758995 | ADCYAP1R1 | NM_001118.3 | 275-380 | 0.46→0.19 |
| rs758995 | ADCYAP1R1 | NM_001118.3 | 275-380 | 0.32→0.11 |
| rs758995 | ADCYAP1R1 | NM_001118.3 | 275-380 | 0.51→0.48 |
| rs758995 | ADCYAP1R1 | NM_001118.3 | 275-380 | 0.59→0.38 |
| rs758995 | ADCYAP1R1 | NM_001118.3 | 275-380 | 0.34→0.12 |
| rs7724759 | CAST | NM_001750.5 | 1066-1104 | 0.12→0.02 |
| rs7724759 | CAST | NM_001750.5 | 1066-1104 | 0.08→0.01 |
| rs7724759 | CAST | NM_001750.5 | 1066-1104 | 0.12→0.02 |
| rs7724759 | CAST | NM_001750.5 | 1066-1104 | 0.06→0.01 |
| rs7865761 | POMT1 | NM_007171.3 | 1351-1443 | 0.55→0.53 |
| rs8178990 | CHAT | NM_020549.4 | 852-905 | 0.18→0.22 |
| rs8192673 | GRB14 | NM_004490.2 | 1836-1923 | 0.31→0.27 |
| rs8192675 | SLC2A2 | NM_000340.1 | 806-921 | 0.48→0.45 |
| rs8192695 | ABCC8 | NM_000352.3 | 417-538 | 0.05→0.02 |
| rs8192695 | ABCC8 | NM_000352.3 | 417-538 | 0.07→0.03 |
| rs8192695 | ABCC8 | NM_000352.3 | 417-538 | 0.1→0.15 |
| rs8383 | PSEN2 | NM_000447.2 | 1619-2298 | 0.04→0.01 |
| rs850713 | GRN | NM_002087.2 | 569-681 | 0.02→0.08 |
| rs850713 | GRN | NM_002087.2 | 682-817 | 0.59→0.62 |
| rs8716 | PFKM | NM_000289.4 | 2369-2909 | 0.17→0.41 |
| rs8716 | PFKM | NM_000289.4 | 2369-2909 | 0.21→0.52 |
| rs9864 | SNCG | NM_003087.1 | 340-411 | 0.26→0.31 |
| **A cryptic exon appears sharing a splice site with an annotated exon** | | | | |
| rs1061170 | CFH | NM_000186.3 | 1400-1576 | 0.08 |
| rs1142530 | NDUFS7 | NM_024407.4 | 97-165 | 0.04 |
| rs11568053 | AGT | NM_000029.3 | 506-1364 | 0.42 |
| rs12435998 | SEL1L | NM_005065.3 | 386-553 | 0.05 |
| rs1332018 | GSTM3 | NM_000849.3 | 1-179 | 0.38 |
| rs1554948 | TNK1 | NM_003985.3 | 70-323 | 0.47 |
| rs16141 | NPY | NM_000905.2 | 87-274 | 0.05 |
| rs17561 | IL1A | NM_000575.3 | 1280-1450 | 0.05 |
| rs17577 | MMP9 | NM_004994.2 | 1921-2024 | 0.04 |
| rs1800206 | PPARA | NM_005036.4 | 636-774 | 0.07 |
| rs1937 | TFAM | NM_003201.1 | 1-233 | 0.3 |
| rs2016520 | PPARD | NM_006238.3 | 209-439 | 0.06 |
| rs20432 | PTGS2 | NM_000963.1 | 774-857 | 0.08 |
| rs2230806 | ABCA1 | NM_005502.2 | 857-1033 | 0.21 |
| rs2285781 | SORCS2 | NM_020777.2 | 2124-2252 | 0.44 |
| rs2302515 | APOBEC1 | NM_001644.3 | 79-476 | 0.28 |
| rs3212319 | CDC2 | NM_001786.2 | 619-782 | 0.05 |
| rs328 | LPL | NM_000237.2 | 1693-1797 | 0.17 |
| rs3767140 | HSPG2 | NM_005529.5 | 615-743 | 0.34 |
| rs4244285 | CYP2C19 | NM_000769.1 | 643-819 | 0.53 |
| rs4604 | ECHS1 | NM_004092.3 | 879-1329 | 0.38 |
| rs560018 | GSTM4 | NM_000850.3 | 569-669 | 0.08 |
| rs6070157 | PCK1 | NM_002591.3 | 388-569 | 0.43 |
| rs668156 | HMGCS2 | NM_005518.2 | 1472-1583 | 0.05 |
| rs6923761 | GLP1R | NM_002062.3 | 463-569 | 0.43 |
| rs8192675 | SLC2A2 | NM_000340.1 | 806-921 | 0.11 |
| **Exon sharing a splice site with an annotated exon disappears** | | | | |
| rs11568053 | AGT | NM_000029.3 | 506-1364 | 0.04 |
| rs12026 | PON2 | NM_000305.2 | 489-615 | 0.03 |
| rs1800194 | LRP1 | NM_002332.2 | 3813-4013 | 0.08 |
| rs1800454 | TAP2 | NM_000544.3 | 1068-1265 | 0.15 |
| rs2024076 | MYH8 | NM_002472.2 | 2149-2266 | 0.03 |
| rs2074308 | ABCC8 | NM_000352.3 | 1798-1943 | 0.39 |
| rs2229765 | IGF1R | NM_000875.3 | 3007-3236 | 0.11 |
| rs2274159 | DFNB31 | NM_015404.2 | 2886-3067 | 0.07 |
| rs2297235 | GSTO2 | NM_183239.1 | 398-662 | 0.96 |
| rs2306325 | SEC24C | NM_004922.2 | 152-351 | 0.15 |
| rs2368564 | REN | NM_000537.2 | 1005-1103 | 0.03 |
| rs3736235 | OLR1 | NM_002543.3 | 679-794 | 0.03 |
| rs3743074 | CHRNA3 | NM_000743.2 | 454-563 | 0.22 |
| rs4362 | ACE | NM_000789.2 | 3403-3525 | 0.11 |
| rs505058 | LMNA | NM_170707.2 | 1407-1629 | 0.6 |
| rs5443 | GNB3 | NM_002075.2 | 1105-1321 | 0.14 |
| rs638405 | BACE1 | NM_012104.3 | 1167-1301 | 0.51 |
| rs700518 | CYP19A1 | NM_031226.2 | 397-547 | 0.19 |
| rs7724759 | CAST | NM_001750.5 | 1066-1104 | 0.03 |
| rs8192690 | ABCC8 | NM_000352.3 | 4735-4978 | 0.35 |
| rs850713 | GRN | NM_002087.2 | 569-681 | 0.27 |
| rs935359 | ENPP2 | NM_006209.3 | 1324-1449 | 0.04 |
| rs9864 | SNCG | NM_003087.1 | 340-411 | 0.4 |
| **A cryptic exon appears** | | | | |
| rs10031715 | APBB2 | ENSG00000163697 | 63013-63133 | 0.24 |
| rs1003857 | RPS6KA2 | ENSG00000071242 | 91341-91460 | 0.04 |
| rs1029353 | AHSG | ENSG00000145192 | 10367-10534 | 0.28 |
| rs1044317 | ABCG1 | ENSG00000160179 | 83638-83721 | 0.03 |
| rs1048101 | ADRA1A | ENSG00000120907 | 97900-97978 | 0.23 |
| rs1076424 | ADORA2B | ENSG00000170425 | 28094-28171 | 0.06 |
| rs1135216 | TAP1 | ENSG00000168394 | 9657-9776 | 0.12 |
| rs1152654 | ADAM12 | ENSG00000148848 | 320434-320476 | 0.09 |
| rs11925306 | MME | ENSG00000196549 | 68124-68247 | 0.03 |
| rs12149 | BACE2 | ENSG00000182240 | 110956-111090 | 0.12 |
| rs12766648 | DNMBP | ENSG00000107554 | 75380-75469 | 0.04 |
| rs1346604 | RFTN1 | ENSG00000131378 | 4985-5076 | 0.28 |
| rs1358337 | CD36 | ENSG00000135218 | 59882-60105 | 0.04 |
| rs1564483 | BCL2 | ENSG00000171791 | 194869-195019 | 0.04 |
| rs16912153 | UBE2D1 | ENSG00000072401 | 3534-3930 | 0.03 |
| rs171649 | PIK3R1 | ENSG00000145675 | 50114-50287 | 0.03 |
| rs1797912 | PPARG | ENSG00000132170 | 142668-142854 | 0.04 |
| rs1800947 | CRP | ENSG00000132693 | 3935-4001 | 0.06 |
| rs1801282 | PPARG | ENSG00000132170 | 65541-65638 | 0.08 |
| rs1805343 | RXRA | ENSG00000186350 | 37812-37836 | 0.13 |
| rs2066853 | AHR | ENSG00000106546 | 43876-43972 | 0.06 |
| rs2069454 | CDK5 | ENSG00000164885 | 5028-5175 | 0.06 |
| rs2071586 | PCK2 | ENSG00000100889 | 11988-12156 | 0.14 |
| rs2072661 | CHRNB2 | ENSG00000160716 | 11384-11621 | 0.41 |
| rs2077624 | RFTN1 | ENSG00000131378 | 104864-105148 | 0.03 |
| rs2234753 | RXRA | ENSG00000186350 | 3002-3282 | 0.04 |
| rs2268345 | HTRA1 | ENSG00000166033 | 27320-27630 | 0.03 |
| rs2284396 | LRP6 | ENSG00000070018 | 147813-147969 | 0.09 |
| rs2290841 | ADAM12 | ENSG00000148848 | 319573-319716 | 0.37 |
| rs2306325 | SEC24C | ENSG00000176986 | 5382-5544 | 0.14 |
| rs2306604 | TFAM | ENSG00000108064 | 6442-6517 | 0.09 |
| rs2575875 | ABCA1 | ENSG00000165029 | 30949-31120 | 0.14 |
| rs2777799 | ABCA1 | ENSG00000165029 | 134386-134469 | 0.07 |
| rs2835908 | KCNJ6 | ENSG00000157542 | 207052-207204 | 0.07 |
| rs2862616 | SOAT1 | ENSG00000057252 | 35411-35515 | 0.09 |
| rs2882676 | ACAN | ENSG00000157766 | 56647-57003 | 0.03 |
| rs3397 | TNFRSF1B | ENSG00000028137 | 43102-43231 | 0.11 |
| rs34044517 | NEDD9 | ENSG00000111859 | 193774-194111 | 0.04 |
| rs3737787 | USF1 | ENSG00000158773 | 9258-9404 | 0.07 |
| rs4149263 | ABCA1 | ENSG00000165029 | 16151-16391 | 0.03 |
| rs440446 | APOE | ENSG00000130203 | 3130-3263 | 0.03 |
| rs4532962 | SORCS3 | ENSG00000156395 | 19873-20069 | 0.03 |
| rs4935775 | SORL1 | ENSG00000137642 | 69458-69539 | 0.19 |
| rs5746136 | SOD2 | ENSG00000112096 | 14071-14174 | 0.05 |
| rs578506 | SORL1 | ENSG00000137642 | 3524-3579 | 0.18 |
| rs600879 | SORCS1 | ENSG00000108018 | 4058-4167 | 0.4 |
| rs6334 | NTRK1 | ENSG00000198400 | 18325-18508 | 0.22 |
| rs6493494 | CYP19A1 | ENSG00000137869 | 69135-69285 | 0.09 |
| rs7893643 | SORCS1 | ENSG00000108018 | 445193-445332 | 0.05 |
| rs872328 | ADAM12 | ENSG00000148848 | 369921-370044 | 0.13 |
| **An exon disappears** | | | | |
| rs1044317 | ABCG1 | ENSG00000160179 | 83640-83721 | 0.05 |
| rs1057335 | SERPINF2 | ENSG00000167711 | 14350-14505 | 0.12 |
| rs1060619 | GAPDH | ENSG00000111640 | 4144-4318 | 0.29 |
| rs10875861 | CCNT1 | ENSG00000129315 | 15843-15974 | 0.07 |
| rs11139063 | APBA1 | ENSG00000107282 | 158033-158148 | 0.03 |
| rs11280 | C6orf130 | ENSG00000124596 | 8392-8456 | 0.07 |
| rs1205 | CRP | ENSG00000132693 | 4951-5131 | 0.21 |
| rs12641331 | APBB2 | ENSG00000163697 | 154506-154844 | 0.04 |
| rs1278319 | ADAM12 | ENSG00000148848 | 263074-263176 | 0.03 |
| rs1358337 | CD36 | ENSG00000135218 | 59867-60047 | 0.05 |
| rs171649 | PIK3R1 | ENSG00000145675 | 50114-50280 | 0.05 |
| rs17561 | IL1A | ENSG00000115008 | 8643-8744 | 0.04 |
| rs1799999 | PPP1R3A | ENSG00000154415 | 43632-43874 | 0.1 |
| rs196295 | BAG3 | ENSG00000151929 | 28417-28478 | 0.09 |
| rs2018621 | POMT1 | ENSG00000130714 | 10311-10502 | 0.15 |
| rs2059806 | INSR | ENSG00000171105 | 130651-130874 | 0.18 |
| rs2228591 | NCOA2 | ENSG00000140396 | 279585-279898 | 0.05 |
| rs2234978 | FAS | ENSG00000026103 | 24556-24805 | 0.03 |
| rs2236319 | SIRT1 | ENSG00000096717 | 7670-7791 | 0.15 |
| rs2237582 | PON1 | ENSG00000005421 | 22548-22643 | 0.24 |
| rs2279015 | SLC11A1 | ENSG00000018280 | 15355-15389 | 0.15 |
| rs2279755 | ADAM12 | ENSG00000148848 | 137775-137789 | 0.03 |
| rs2284396 | LRP6 | ENSG00000070018 | 147819-147969 | 0.06 |
| rs2299261 | PON1 | ENSG00000005421 | 7192-7293 | 0.25 |
| rs2299267 | PON2 | ENSG00000105854 | 5273-5373 | 0.09 |
| rs2306604 | TFAM | ENSG00000108064 | 6529-6777 | 0.05 |
| rs2781530 | APBA1 | ENSG00000107282 | 233833-234014 | 0.03 |
| rs363717 | ABCA1 | ENSG00000165029 | 148738-148889 | 0.06 |
| rs370088 | SORT1 | ENSG00000134243 | 85400-85451 | 0.04 |
| rs3742376 | CYP46A1 | ENSG00000036530 | 17983-18231 | 0.04 |
| rs3773882 | MME | ENSG00000196549 | 79754-79857 | 0.36 |
| rs3827225 | ABCG1 | ENSG00000160179 | 17531-17768 | 0.03 |
| rs440446 | APOE | ENSG00000130203 | 3143-3194 | 0.05 |
| rs4832 | ACAT2 | ENSG00000120437 | 19851-19943 | 0.08 |
| rs4998 | ADRB3 | ENSG00000188778 | 5562-5698 | 0.48 |
| rs5104 | APOA4 | ENSG00000110244 | 4437-4682 | 0.12 |
| rs5498 | ICAM4 | ENSG00000105371 | 854-1000 | 0.46 |
| rs556349 | SORL1 | ENSG00000137642 | 106102-106247 | 0.06 |
| rs6923761 | GLP1R | ENSG00000112164 | 20464-20676 | 0.08 |
| rs6959138 | CCDC126 | ENSG00000169193 | 32196-32298 | 0.32 |
| rs714816 | HTRA1 | ENSG00000166033 | 38131-38176 | 0.19 |
| rs7294695 | LRP6 | ENSG00000070018 | 99045-99128 | 0.4 |
| rs730179 | TLL2 | ENSG00000095587 | 105234-105346 | 0.12 |
| rs736824 | HNF4A | ENSG00000101076 | 7749-7876 | 0.09 |
| rs7374 | DHCR24 | ENSG00000116133 | 39453-39568 | 0.46 |
| rs741073 | NGFR | ENSG00000064300 | 22116-22231 | 0.19 |
| rs8123020 | PCK1 | ENSG00000124253 | 3856-3926 | 0.14 |
| rs8192675 | SLC2A2 | ENSG00000163581 | 22888-23047 | 0.57 |
| rs890 | GRIN2B | ENSG00000150086 | 420761-421009 | 0.11 |
| rs978903 | PON3 | ENSG00000105852 | 24134-24494 | 0.06 |

**Legend:** Here we predicted probabilistic exon scores change which depends on exon length, 5’SS and 3’SS strengths and the effect of exonic splicing enhancers/silencers. The arrow indicates change of associated exonic probabilistic score from allele variant present in HG18 reference genome to alternative polymorphic variant.

**Table 2**. SNPs associated with the breast cancer predicted to change the pattern of splicing.

| **SNP ID** | **Gene** | **Sequence ID** | **Affected exon coordinates** | **Associated score change** |
| --- | --- | --- | --- | --- |
| **Score of an annotated exon changes** | | | | |
| rs42046 | CDK6 | NM_001259.5 | 1041-1091 | 0.61→0.59 |
| rs1139793 | TXNRD2 | NM_006440.3 | 1120-1215 | 0.81→0.79 |
| rs2508740 | C11orf30 | NM_020193.3 | 1657-1827 | 0.46→0.42 |
| rs2424928 | DNMT3B | NM_006892.3 | 2227-2317 | 0.81→0.73 |
| rs3092904 | RB1 | NM_000321.2 | 2830-2879 | 0.46→0.52 |
| rs3092904 | RB1 | NM_000321.2 | 2830-2879 | 0.46→0.34 |
| rs3092904 | RB1 | NM_000321.2 | 2830-2879 | 0.46→0.42 |
| rs2229765 | IGF1R | NM_000875.3 | 3007-3236 | 0.61→0.64 |
| rs4680 | COMT | NM_000754.2 | 492-685 | 0.75→0.73 |
| rs3731249 | CDKN2A | NM_058197.3 | 637-943 | 0.98→0.96 |
| rs9344 | CCND1 | NM_053056.2 | 784-932 | 0.93→0.81 |
| **Score of an exon sharing a splice site with an annotated exon changes** | | | | |
| rs1042522 | TP53 | NM_001126114.1 | 272-293 | 0.35→0.59 |
| rs1042522 | TP53 | NM_001126114.1 | 294-572 | 0.36→0.6 |
| rs1139793 | TXNRD2 | NM_006440.3 | 1120-1215 | 0.22→0.19 |
| rs1139793 | TXNRD2 | NM_006440.3 | 1120-1215 | 0.39→0.36 |
| rs1410492 | CCND3 | NM_001760.2 | 364-579 | 0.23→0.17 |
| rs1625895 | TP53 | NM_001126114.1 | 757-869 | 0.65→0.61 |
| rs1799793 | ERCC2 | NM_000400.2 | 847-980 | 0.83→0.92 |
| rs1799793 | ERCC2 | NM_000400.2 | 847-980 | 0.83→0.92 |
| rs1799793 | ERCC2 | NM_000400.2 | 847-980 | 0.07→0.16 |
| rs1799793 | ERCC2 | NM_000400.2 | 847-980 | 0.95→0.97 |
| rs1799793 | ERCC2 | NM_000400.2 | 847-980 | 0.7→0.86 |
| rs1799793 | ERCC2 | NM_000400.2 | 847-980 | 0.48→0.7 |
| rs1799794 | XRCC3 | NM_001100119.1 | 64-157 | 0.28→0.26 |
| rs1799794 | XRCC3 | NM_001100119.1 | 64-157 | 0.59→0.57 |
| rs1801132 | ESR1 | NM_001122740.1 | 1022-1357 | 0.71→0.51 |
| rs1801270 | CDKN1A | NM_078467.1 | 231-680 | 0.9→0.75 |
| rs206118 | BRCA2 | NM_000059.3 | 1-188 | 0.16→0.3 |
| rs206118 | BRCA2 | NM_000059.3 | 1-188 | 0.13→0.25 |
| rs206118 | BRCA2 | NM_000059.3 | 1-188 | 0.11→0.21 |
| rs206118 | BRCA2 | NM_000059.3 | 1-188 | 0.07→0.15 |
| rs206118 | BRCA2 | NM_000059.3 | 1-188 | 0.36→0.54 |
| rs206118 | BRCA2 | NM_000059.3 | 1-188 | 0.2→0.36 |
| rs206118 | BRCA2 | NM_000059.3 | 1-188 | 0.63→0.78 |
| rs2229571 | BARD1 | NM_000465.2 | 500-1449 | 0.03→0.06 |
| rs2229765 | IGF1R | NM_000875.3 | 3007-3236 | 0.21→0.23 |
| rs2229765 | IGF1R | NM_000875.3 | 3007-3236 | 0.21→0.23 |
| rs2286196 | PARP12 | NM_022750.2 | 2372-2502 | 0.21→0.26 |
| rs2287499 | WDR79 | NM_018081.1 | 1-603 | 0.57→0.4 |
| rs2289195 | DNMT3A | NM_175629.1 | 2512-2660 | 0.11→0.13 |
| rs2424928 | DNMT3B | NM_006892.3 | 2227-2317 | 0.17→0.11 |
| rs2424928 | DNMT3B | NM_006892.3 | 2227-2317 | 0.19→0.12 |
| rs2479717 | CCND3 | NM_001760.2 | 580-739 | 0.1→0.07 |
| rs2479717 | CCND3 | NM_001760.2 | 580-739 | 0.07→0.04 |
| rs2479717 | CCND3 | NM_001760.2 | 580-739 | 0.55→0.86 |
| rs2508740 | C11orf30 | NM_020193.3 | 1657-1827 | 0.1→0.08 |
| rs2508740 | C11orf30 | NM_020193.3 | 1657-1827 | 0.14→0.11 |
| rs2508740 | C11orf30 | NM_020193.3 | 1657-1827 | 0.16→0.19 |
| rs3092904 | RB1 | NM_000321.2 | 2830-2879 | 0.01→0.04 |
| rs3092904 | RB1 | NM_000321.2 | 2830-2879 | 0.22→0.18 |
| rs3092904 | RB1 | NM_000321.2 | 2830-2879 | 0.01→0.05 |
| rs3092904 | RB1 | NM_000321.2 | 2830-2879 | 0.05→0.03 |
| rs3092904 | RB1 | NM_000321.2 | 2830-2879 | 0.22→0.16 |
| rs3092904 | RB1 | NM_000321.2 | 2830-2879 | 0.22→0.19 |
| rs3217805 | CCND2 | NM_001759.2 | 681-840 | 0.5→0.44 |
| rs3217805 | CCND2 | NM_001759.2 | 681-840 | 0.23→0.19 |
| rs3731249 | CDKN2A | NM_058197.3 | 637-943 | 0.31→0.16 |
| rs3731249 | CDKN2A | NM_058197.3 | 637-943 | 0.46→0.23 |
| rs3731249 | CDKN2A | NM_058197.3 | 637-943 | 0.24→0.1 |
| rs4680 | COMT | NM_000754.2 | 492-685 | 0.75→0.73 |
| rs5748469 | TXNRD2 | NM_006440.3 | 206-262 | 0.39→0.21 |
| rs5748469 | TXNRD2 | NM_006440.3 | 206-262 | 0.39→0.21 |
| rs602652 | CCND1 | NM_053056.2 | 784-932 | 0.13→0.11 |
| rs602652 | CCND1 | NM_053056.2 | 784-932 | 0.13→0.11 |
| rs756661 | TXNRD2 | NM_006440.3 | 408-482 | 0.06→0.25 |
| rs9344 | CCND1 | NM_053056.2 | 784-932 | 0.23→0.07 |
| rs9344 | CCND1 | NM_053056.2 | 784-932 | 0.13→0.04 |
| rs9344 | CCND1 | NM_053056.2 | 784-932 | 0.7→0.42 |
| rs9344 | CCND1 | NM_053056.2 | 784-932 | 0.84→0.63 |
| rs9344 | CCND1 | NM_053056.2 | 784-932 | 0.83→0.56 |
| **A cryptic exon appears sharing a splice site with an annotated exon** | | | | |
| rs1139793 | TXNRD2 | NM_006440.3 | 1120-1215 | 0.09 |
| rs1799783 | ERCC2 | NM_000400.2 | 278-391 | 0.04 |
| rs2276599 | DNMT3A | NM_175629.1 | 1353-1460 | 0.06 |
| rs3092904 | RB1 | NM_000321.2 | 2830-2879 | 0.04 |
| rs602652 | CCND1 | NM_053056.2 | 784-932 | 0.3 |
| **Exon sharing a splice site with an annotated exon disappears** | | | | |
| rs1042838 | PGR | NM_000926.4 | 2650-2955 | 0.03 |
| rs2229765 | IGF1R | NM_000875.3 | 3007-3236 | 0.11 |
| rs2234996 | ATM | NM_000051.3 | 717-881 | 0.03 |
| rs3092904 | RB1 | NM_000321.2 | 2830-2879 | 0.03 |
| rs42046 | CDK6 | NM_001259.5 | 1041-1091 | 0.48 |
| rs9344 | CCND1 | NM_053056.2 | 784-932 | 0.03 |
| **A cryptic exon appears** | | | | |
| rs1033181 | ESR1 | ENSG00000091831 | 69064-69135 | 0.1 |
| rs11391 | HDAC2 | ENSG00000196591 | 33310-33337 | 0.55 |
| rs11571171 | PGR | ENSG00000082175 | 29168-29363 | 0.07 |
| rs11920625 | ATR | ENSG00000175054 | 39329-39451 | 0.05 |
| rs1379130 | PGR | ENSG00000082175 | 5407-5631 | 0.91 |
| rs1410051 | TXN | ENSG00000136810 | 4196-4435 | 0.09 |
| rs1805839 | NBN | ENSG00000104320 | 7664-7775 | 0.04 |
| rs2070424 | SOD1 | ENSG00000142168 | 10161-10238 | 0.25 |
| rs2241531 | DNMT1 | ENSG00000130816 | 37786-37892 | 0.18 |
| rs2267692 | TBXAS1 | ENSG00000059377 | 71003-71153 | 0.5 |
| rs2286196 | PARP12 | ENSG00000059378 | 39022-39097 | 0.17 |
| rs2291857 | MDM2 | ENSG00000135679 | 18876-19056 | 0.51 |
| rs2911678 | GSR | ENSG00000104687 | 48370-48475 | 0.12 |
| rs3217795 | CCND2 | ENSG00000118971 | 6078-6126 | 0.21 |
| rs3788306 | TXNRD2 | ENSG00000184470 | 60438-60507 | 0.19 |
| rs4149533 | SULT1E1 | ENSG00000109193 | 10636-10835 | 0.07 |
| rs542551 | BRCA2 | ENSG00000139618 | 72802-72917 | 0.26 |
| rs584330 | MBD2 | ENSG00000134046 | 57058-57210 | 0.15 |
| rs611018 | ATM | ENSG00000149311 | 90633-90775 | 0.03 |
| rs6504074 | BRIP1 | ENSG00000136492 | 43726-43962 | 0.04 |
| rs8 | CDK6 | ENSG00000105810 | 57903-58029 | 0.04 |
| rs858524 | FXR2 | ENSG00000129245 | 9884-9980 | 0.39 |
| rs9341066 | ESR1 | ENSG00000091831 | 293809-294139 | 0.03 |
| rs9534174 | BRCA2 | ENSG00000139618 | 7176-7247 | 0.15 |
| **An exon disappears** | | | | |
| rs1201644 | MDM2 | ENSG00000135679 | 15561-15711 | 0.53 |
| rs13447720 | MRE11A | ENSG00000020922 | 64687-64786 | 0.24 |
| rs1465764 | DNMT3A | ENSG00000119772 | 7455-7564 | 0.65 |
| rs16979884 | CSTF1 | ENSG00000101138 | 14182-14412 | 0.3 |
| rs1805839 | NBN | ENSG00000104320 | 7661-7775 | 0.07 |
| rs1884054 | ESR1 | ENSG00000091831 | 165890-165915 | 0.06 |
| rs1951775 | RB1 | ENSG00000139687 | 141156-141298 | 0.13 |
| rs2069408 | CDK2 | ENSG00000123374 | 6765-6967 | 0.39 |
| rs2075623 | BARD1 | ENSG00000138376 | 83531-83725 | 0.09 |
| rs2267692 | TBXAS1 | ENSG00000059377 | 71011-71153 | 0.25 |
| rs2284205 | TBXAS1 | ENSG00000059377 | 94244-94436 | 0.55 |
| rs228769 | HDAC5 | ENSG00000108840 | 10815-11182 | 0.04 |
| rs2295464 | C1orf190 | ENSG00000171357 | 59862-59935 | 0.51 |
| rs2459107 | ESR1 | ENSG00000091831 | 273941-274096 | 0.17 |
| rs2854345 | RB1 | ENSG00000139687 | 16328-16510 | 0.62 |
| rs2911678 | GSR | ENSG00000104687 | 48411-48471 | 0.13 |
| rs3020396 | ESR1 | ENSG00000091831 | 154128-154188 | 0.09 |
| rs3125795 | EHMT1 | ENSG00000181090 | 50123-50199 | 0.24 |
| rs3218038 | CCNE1 | ENSG00000105173 | 5935-5992 | 0.06 |
| rs3731343 | CDK6 | ENSG00000105810 | 192675-192781 | 0.03 |
| rs3778216 | HDAC2 | ENSG00000196591 | 14660-14760 | 0.25 |
| rs3817198 | LSP1 | ENSG00000130592 | 37715-37804 | 0.03 |
| rs4752894 | PTPRJ | ENSG00000149177 | 121714-121819 | 0.12 |
| rs584330 | MBD2 | ENSG00000134046 | 57046-57286 | 0.21 |
| rs599164 | ATM | ENSG00000149311 | 25120-25195 | 0.06 |
| rs613120 | PGR | ENSG00000082175 | 29987-30068 | 0.03 |
| rs8030950 | IGF1R | ENSG00000140443 | 214253-214341 | 0.03 |
| rs8140110 | TXN2 | ENSG00000100348 | 11939-12048 | 0.18 |
| rs827421 | ESR1 | ENSG00000091831 | 31454-31622 | 0.06 |
| rs858524 | FXR2 | ENSG00000129245 | 9892-9980 | 0.04 |

**Legend:** Here we predicted probabilistic exon scores change which depends on exon length, 5’SS and 3’SS strengths and the effect of exonic splicing enhancers/silencers. The arrow indicates change of associated exonic probabilistic score from allele variant present in HG18 reference genome to alternative polymorphic variant.
