# Supplementary material for: A method of predicting changes in human gene splicing induced by genetic variants in context of cis-acting elements
Source: BMC Bioinformatics. 2010 Jan 12;11:22. doi: 10.1186/1471-2105-11-22 (PMC3098058; doi:10.1186/1471-2105-11-22)
Supplement: Additional file 4 — Splicing regulatory elements reported in [40]and their statistical significance. Repertoire of exonic and intronic splicing enhancer/silencer elements used in building of SpliceScan II tool. [file 1471-2105-11-22-S4.PDF]

Supplementary materials

1 Found Elements

1.1 5′SS ISEs/ISSs

D.IE.1	0.034	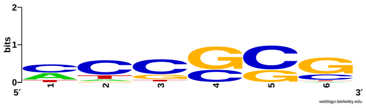	ACCGCG, CTCGCG
D.IE.2	0.021	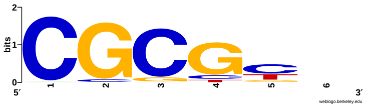	CGCGTC, CGCTTTT, GACGCTG
D.IE.3	0.073	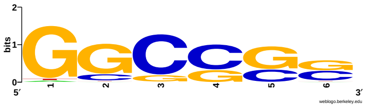	AGGCCGGG, AGGGCCGG, AGGGCGGG, CAGCCGG, CCGGCCGG, CCTCCCCG, CCTGCCCG, CGGCCCG, CGGCCGC, CGGCCGG, CGGCCGGG, CGGGCCGG, GCAGCCCC, GCTGCCCC, GGCCCCTC, GGCCCGG, GGCCCGGC, GGCCCGT, GGCCGC, GGCCGCA, GGCCGG, GGCCGGC, GGCCGGG, GGCCGGGA, GGCCGGGC, GGCCGTGG, GGCGCC, GGCGCG, GGCGCGG, GGCGCTGG, GGCGGCC, GGCTCGG, GGGCCCGG, GGGCCGC, GGGCCGG, GGGCCGGC, GGGCCGGG, GGGCGCG, GGGCGGC, GGGCGGCC, GGGGCCGC, GTGCCGC, TGGGCCGG, TGGGCGGG
D.IE.4	0.016	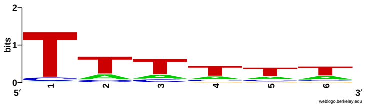	ACTTTAT, ACTTTTTT, ATATTTTT, ATCATTTT, ATCCTTT, ATTATTA, ATTTATT, ATTTATTT, ATTTTATT, ATTTTCAT, ATTTTGTT, ATTTTTTT, CTATCTA, CTTTATTT, CTTTGAT, CTTTTAAA, CTTTTATT, CTTTTTCT, TAATTTC, TATTCAT, TATTTTAT, TATTTTCT, TATTTTTT, TCTCTCTC, TTTATTC, TTTCTTTA, TTTCTTTT, TTTGAGAC, TTTTAATT, TTTTCTTA, TTTTTAAA, TTTTTATT, TTTTTCCT, TTTTTCTT, TTTTTTA, TTTTTTAA, TTTTTTCT, TTTTTTTA
D.IE.5	0.20	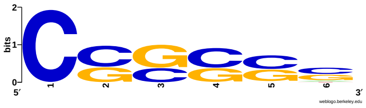	ACCGCCC, ACCGGCC, ATCCCAGC, CCCCCCGC, CCCCCGC, CCCCCGCC, CCCCCGGC, CCCCGCC, CCCCGCCC, CCCCGCG, CCCCGGCC, CCCGACC, CCCGCC, CCCGCCC, CCCGCCCC, CCCGCCCG, CCCGCCCT, CCCGCCG, CCCGCCT, CCCGCG, CCCGCGC, CCCGCGG, CCCGCTT, CCCGGCC, CCCGGCCC, CCCGGCG, CCGCCC, CCGCCCC, CCGCCCCC, CCGCCCCG, CCGCCCCT, CCGCCCG, CCGCCCGC, CCGCCCTG, CCGCCG, CCGCCGC, CCGCCGG, CCGCG, CCGCGC, CCGCGCC, CCGCGG, CCGCGGC, CCGCGGCC, CCGCGGG, CCGCGGGG, CCGGCAG, CCGGCC, CCGGCCC, CCGGCCCC, CCGGCCCT, CCGGCCG, CCGGCG, CCGGCGG, CCGGGAGG, CCGGGCC, CCGGGCG, CCGGGCGG, CGCCCCC, CGCCCG, CGCCCGG, CGCCCTT, CGCCGC, CGCCGG, CGCGC, CGCGCC, CGCGCCC, CGCGCG, CGCGGC, CGCGGCC, CGGCCCC, CGGCCCCC, CGGCCG, CGGCGG, CGGGAGGG, CGGGCAG, CGGGCAGG, CGGGCC, CGGGCG, CGGGCGG, CGGGCGGG, GACCAGCC, GCCCCCGC, GCCCCGCC, GCCCGCCC, GCCCGGCC, GCCGCCG, GCCGGGC, GCCGGGCC, GCCGGGCG, GCGCCCG, GCGCCGC, GCGGCCC, GCGGCCCC, GCGGCCG, TCCGCCC

D.IE.6	0.052	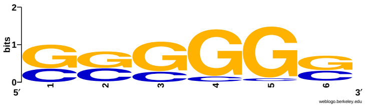	ACGGGGC, AGGCGGGG, AGGGGGGC, ATGGGGGG, CAGGCGGG, CGCGGG, CGCGGGC, CGCGGGG, CGGCGGG, CGGCTGGG, CGGGGC, CGGGGCA, CGGGGCAG, CGGGGCTG, CGGGGGC, CGGGGGG, CGGGGGGC, CGGGGGGG, CGGGGGT, CTCGGGGC, CTGCGGGG, CTGGCGGG, CTGGGGGC, CTGGGGGG, GAGGGGGC, GAGGGGGG, GCAGGGCC, GCGGGAGG, GCGGGC, GCGGGCC, GCGGGCG, GCGGGCGG, GCGGGCT, GCGGGG, GCGGGGAG, GCGGGGC, GCGGGGCC, GCGGGGCT, GCGGGGG, GCGGGGGC, GCGGGGGG, GCGGGGT, GGCGGGAG, GGCGGGC, GGCGGGCC, GGCGGGCG, GGCGGGG, GGCGGGGC, GGCGGGGG, GGGCGGG, GGGCGGGA, GGGCGGGC, GGGCGGGG, GGGGGCAG, GGGGGGAG, GGGGGGC, GGGGGGCA, GGGGGGGC, GGGTGGGG, GGTGGGGA, GGTGGGGG, GGTGGGGT, GTGGGGGC, GTGGGGGG, TAGCTGGG, TGCGGGG, TGGGGGGC
D.IE.7	0.14	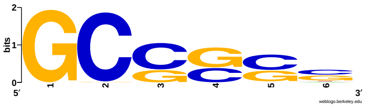	CGCCCCG, CGCCCGC, CGCCGCC, CGCCGGC, CGCCGGG, CTGCCCCG, CTGCCCGC, CTGCCCGG, GCCCCCCC, GCCCCCCG, GCCCCCG, GCCCCCGG, GCCCCCTC, GCCCCCTG, GCCCCG, GCCCCGC, GCCCCGG, GCCCCGGC, GCCCCTCC, GCCCCTGC, GCCCGC, GCCCGCC, GCCCGCG, GCCCGG, GCCCGGC, GCCCGTC, GCCCTCCC, GCCGC, GCCGCAC, GCCGCC, GCCGCCC, GCCGCCCC, GCCGCG, GCCGCGC, GCCGCGG, GCCGCGGG, GCCGCTC, GCCGGC, GCCGGCC, GCCGGCCC, GCCGGCG, GCCGGG, GCCGGGCT, GCCTGCCC, GCGCCC, GCGCCCC, GCGCCG, GCGCCGG, GCGCCTC, GCGCGC, GCGCGG, GCGCGGC, GCGCGGG, GCGGCG, GCGGCGG, GCGGCGGG, GGCCCCCG, GGCCCCCT, GGCCCCG, GGCCCCGC, GGCCCCGG, GGCCCGC, GGCCGCCC, GGCCGCG, GGCCGCGG, GGCCGGCC, GGCGCCC, GGCGCCG, GGCGGCG, TGCGCCC, TGGCCCCG
D.IE.8	0.081	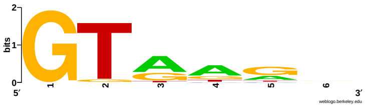	AAGTAAG, AGTAAGT, AGTACAG, AGTAGCTG, AGTGCAGT, ATGTAAG, CAGTAAG, CGGTGAG, CTGTAATC, GAGTGCAG, GGGTGGGA, GGGTGGGC, GGTAAGT, GGTAGAT, GGTAGGA, GGTAGGT, GGTATGT, GGTGAGC, GGTGAGT, GGTGAGTG, GGTTAAG, GGTTCAA, GTAAG, GTAAGCA, GTAAGGA, GTAAGT, GTAAGTA, GTAAGTC, GTAAGTG, GTAAGTT, GTAATCC, GTAGAAC, GTGAAG, GTGAGT, GTGAGTA, GTGCAA, GTGGCTCA, GTTAGTA, TAGTAAG, TAGTTAG, TAGTTGC, TGGAGTGC, TGTAAGT, TGTAATCC
D.IE.9	0.034	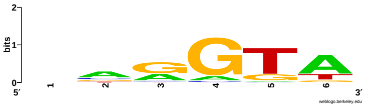	AAAGGT, AAAGGTA, ACAGGTA, AGAGGTA, ATAGGTA, CAAGGTA, CCAGGTA, CCCAGGTT, CTGAGGCA, GAAGGTA, GCAGGTA, GCAGGTG, GCCAGGCG, GCCAGGGT, GGAGGTA, GGCAAGT, GGCAGGT, GGGAGGGT, GGTCAGT, GTAGGTA, TAAGGTA, TCAGGTA, TGAGGTA, TTAGGTA
D.IE.10	0.012	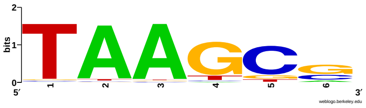	AATCCCAG, TAAGCAC, TAAGCG, TAAGTGC, TAATCCC, TAATCCCA
D.IE.11	0.16	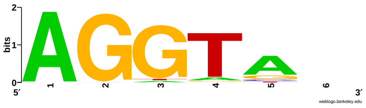	AAGGTA, AAGGTAA, AAGGTAG, AAGGTAT, AAGGTGA, AAGGTGAG, AGACCAGC, AGAGACAG, AGGCAAG, AGGTA, AGGTAA, AGGTAAA, AGGTAAC, AGGTAAG, AGGTAAT, AGGTAC, AGGTACA, AGGTACT, AGGTAG, AGGTAGA, AGGTAGG, AGGTAGT, AGGTAT, AGGTATA, AGGTATG, AGGTATT, AGGTCAG, AGGTCAGG, AGGTCT, AGGTGA, AGGTGAA, AGGTGAG, AGGTGAGG, AGGTGC, AGGTGGG, AGGTTAG, AGTATAG, CAGATAG, CAGGAGAA, CAGGAGTT, CAGGTA, CAGGTAA, CAGGTAC, CAGGTAG, CAGGTAT, CAGGTGA, CAGGTGAG, GAGAAAGA, GAGATAG, GAGGAAGA, GAGGTA, GAGGTAA, GAGGTAG, GAGGTCAG, GAGGTGA, GCAGGTGA, TAGATAG, TAGATGA, TAGGACT, TAGGTA, TAGGTAA, TAGGTAG, TAGGTAT, TAGGTCA, TAGGTGA, TAGGTTG

D.IE.12	0.052	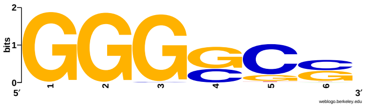	AGGGCGG, AGGGGCGG, AGGGGGCC, AGGGGGTG, ATGGGCAG, CAGGGCCG, CCGGGCCC, CCGGGCCG, CGGGCCC, CGGGCCCC, CGGGCCG, CGGGCGC, CGGGCTGG, CGGGGAC, CGGGGCC, CGGGGCCC, CGGGGCCG, CGGGGCCT, CGGGGCG, CGGGGCGG, CGGGGGCC, CGGGGTG, CGGGGTGG, CTGGGCCC, CTGGGCCG, CTGGGCGG, CTGGGGCC, CTGGGGCG, GAGGGGAC, GCGGGGCG, GGGCAGGG, GGGCCCC, GGGCCCCC, GGGCCCCG, GGGCCCCT, GGGCCCG, GGGCCCTG, GGGCCG, GGGCCTGG, GGGCGCC, GGGCGCTG, GGGCTCG, GGGCTCGG, GGGCTGGG, GGGGCACC, GGGGCAGG, GGGGCC, GGGGCCC, GGGGCCCA, GGGGCCCC, GGGGCCCG, GGGGCCCT, GGGGCCG, GGGGCCGG, GGGGCCT, GGGGCCTC, GGGGCCTG, GGGGCG, GGGGCGC, GGGGCGCC, GGGGCGG, GGGGCGGC, GGGGCGGG, GGGGCTGC, GGGGCTGG, GGGGGCC, GGGGGCCC, GGGGGCCG, GGGGGCG, GGGGGCGG, GGGGGGCC, GGGGGGCG, GGGGTGGG, GTGGGCCC, GTGGGCGG, GTGGGGAC, GTGGGGCC, GTGGGGCG, TGGGCCCC, TGGGGCAC, TGGGGCC, TGGGGCCA, TGGGGCCC, TGGGGCCG, TGGGGCCT, TGGGGCG, TGGGGCGG, TGGGGGCC, TGGGGGCG
D.IE.13	0.053	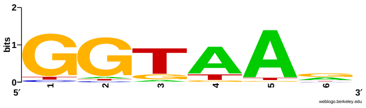	CCTGTAA, CCTGTAAT, CGGGTGGG, GGGTAAG, GGTAA, GGTAAAG, GGTAAAT, GGTAAG, GGTAAGA, GGTAAGG, TCTGTAAA, TGGTAAG
D.IE.14	0.027	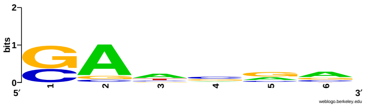	ACACACA, ACACACAC, AGAAGAAG, AGACAGAG, AGAGAGA, AGAGAGAC, AGAGAGAG, CAACGA, CACACAC, CACACACA, CACCACCA, CAGAAGAG, CAGAGAAG, CAGAGAGA, GAAGGA, GAGAAGCA, GAGACAGA, GAGAGACA, GAGAGAGA, GATCGA, GGACTAC, TACAGATG
D.IE.15	0.043	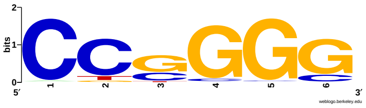	ACCCTCCT, ACCGGGC, ACCGGGG, ACTGGGCC, ACTGGGGA, CACCGGG, CCAGGCGG, CCAGGGGC, CCCATGGG, CCCCCGGG, CCCCGGG, CCCCGGGC, CCCCGGGG, CCCGGG, CCCGGGC, CCCGGGCA, CCCGGGCC, CCCGGGG, CCCGGGGA, CCCGGGGC, CCCGGGGG, CCCTCCCG, CCCTGCCG, CCGGGC, CCGGGCAG, CCGGGCT, CCGGGCTG, CCGGGG, CCGGGGA, CCGGGGC, CCGGGGCA, CCGGGGCC, CCGGGGCG, CCGGGGCT, CCGGGGG, CCGGGGGC, CCGGGGGG, CCGGGGT, CCGGGGTG, CCTGGGCG, CCTGGGGC, CTCGGGG, CTGGGGAC, CTGGGGC, CTGGGGCA, CTGGGGTC, GCCAGGGC, GCCAGGGG, GCCCCGGG, GCCCGGG, GCCCGGGC, GCCCGGGG, GCCGGGG, GCCGGGGA, GCCGGGGC, GCCGGGGG, GCCTGGGG, GCTCGGG, GCTGGGCC, GCTGGGGC, GCTGGGGG, GCTTGGGG, GGCCAGGG, GGCCCGGG, GGCCGGGG, GGCTCGGG, GGCTGGGC, GGCTGGGG, TCCGGGG

1.2 3′SS ISEs/ISSs

A.IE.1	0.018	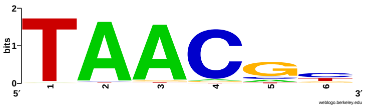	AATAACAT, AATGTAAC, AGTAACTT, ATAACATT, ATAACTGA, GTAACATT, GTAACTTT, GTTAACAT, GTTAACC, TAACACT, TAACACTT, TAACATTT, TAACCATT, TAACCCTT, TAACCTTT, TAACGC, TAACGT, TAACGTG, TAACGTT, TAACTCTT, TAACTGTG, TAACTTTG, TAATGTGT, TGTAACTT, TTAACACT, TTAACCTT, TTAACTCA, TTAACTCT, TTAACTGA, TTAACTTA, TTAATTTT, TTTAACAA, TTTAACG, TTTGTAAC
A.IE.2	0.048	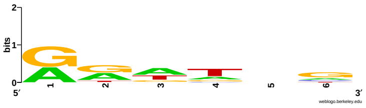	AAAATGTT, AAGATAG, AAGATCC, AAGATGG, ACATTGT, AGAATAG, AGAATGG, AGATAG, AGATAGA, AGATAGG, AGATCG, AGATGAG, AGATGAGG, AGATGGA, AGATGGG, AGATTAG, AGGATAG, AGGATGG, ATATTAT, ATTATTG, ATTTTATT, CAGATAG, CAGATGAG, GAATTGG, GAGATAG, GAGATGG, GATATTAA, GATGAGG, GATTCG, GATTTGAA, GGAATAG, GGATAGA, GGATCAG, GGATCG, GGATGAG, GGATGGA, GGATGGG, GGATTAC, GGATTAG, GGATTGG, GGGATGG, GGGATGGG, GGGATTA, GGGATTG, TAAATTG, TAATCATT, TAATTGAT, TAATTTCT, TAGATAG, TAGATGG, TATTGATG, TGGATAG, TGGGATTA, TTGATTAA
A.IE.3	0.028	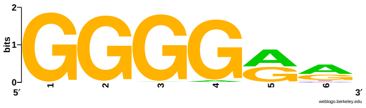	AGGGGAA, AGGGGAAG, AGGGGAG, AGGGGAGG, AGGGGG, AGGGGGA, AGGGGGAG, AGGGGGG, CGGGGAG, CGGGGAGG, CTGGGGAG, GAGGGGAG, GCGGGGG, GGGGAA, GGGGAAA, GGGGAAAA, GGGGAAC, GGGGAAG, GGGGAAGG, GGGGAG, GGGGAGA, GGGGAGG, GGGGATG, GGGGGA, GGGGGAA, GGGGGAG, GGGGGAGG, GGGGGCA, GGGGGG, GGGGGGA, GGGGGGG, GGGGGGGG, GTGGGGAG, GTGGGGGG, TGGGGAA, TGGGGAAG, TGGGGAG, TGGGGAGA, TGGGGAGG, TGGGGGA, TGGGGGAG, TGGGGGG, TTGGGGA, TTGGGGAG, TTGGGGG, TTTGGGAA
A.IE.4	0.041	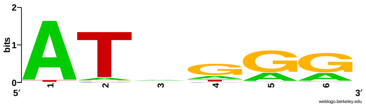	AAAATGGA, AATAGGA, AATAGGG, AATTAGG, ATAGAGG, ATAGGAA, ATAGGAG, ATAGGCA, ATAGGG, ATAGGGA, ATAGGGT, ATATGGG, ATATTTTT, ATCAAGG, ATCAGAG, ATCGAG, ATCGGG, ATGGAGG, ATGGGAG, ATGGGGA, ATGGGGG, ATTGGAG, ATTGGGA, ATTGGGG, CATAGAG, CATAGGA, CATAGGG, CATTAGG, CTATGGG, GAAATGGA, GAATGGG, GATAAGG, GATAGAG, GATAGGA, GATGGAG, GATTAGA, GATTGGG, GCATTAG, TATAGG, TATAGGA, TATATTA, TATATTTT, TATGAGG, TATGGGG, TATTAGC, TCGGGA, TTCGGA, TTCGGG

A.IE.5	0.046	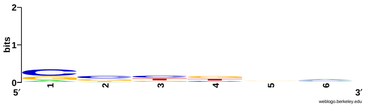	AAACTGAC, AAATGACC, AACTGAC, AAGTGCTG, ACACTGAC, ACGCTGA, AGACTGAC, AGAGCTGG, AGCCCTGA, AGCTGAC, AGGCTGAC, AGGGCTGG, AGTGCTGG, ATACTGAT, ATGACTGA, ATGCTGAC, ATGCTGAT, ATTCTGAC, CACCCTGA, CACTGAC, CACTGAT, CACTGATT, CAGCTGAC, CAGGCTGG, CAGTGACC, CATCTGAC, CATGCTGA, CCACCTGA, CCACTGAC, CCACTGAT, CCACTGCA, CCAGCTGA, CCCACTGA, CCCCCTGA, CCCCGGC, CCCGGCC, CCCTGAT, CCGCTCA, CCGCTGA, CCGCTGAC, CCGTGAC, CCTGCTCA, CCTGTGAC, CGCTCAC, CGCTGA, CGCTGAC, CGTGACC, CTCACTGA, CTCCCTGA, CTCGCTC, CTGCTGAC, CTGCTGAT, CTGGCTGA, CTTACTGA, CTTCTGAC, CTTCTGAT, CTTGCTGA, CTTGTGAC, GACTGAC, GAGCTGAC, GAGCTGG, GAGCTGGA, GAGCTGGG, GAGGCTGG, GATGCTGA, GCACTCAC, GCACTGAC, GCAGCTGG, GCCCCTCA, GCCCCTGA, GCCCTGAT, GCCGCTC, GCGCTGA, GGACTGAC, GGAGCTGG, GGCACTCA, GGCCCTCA, GGCCCTGA, GGCCTGA, GGCTGAC, GGGACTGA, GGGCCTGA, GGGCTGAC, GGGGCTGA, GGTCCTGA, GGTCTGAC, GGTGCTGG, GTCCCTGA, GTCTGAC, GTCTGAT, GTGACTCT, GTGCTGAC, GTGCTGAT, GTGCTGGG, GTTCTGAC, GTTCTGAT, TAACTGAT, TAATGACT, TACTGAC, TAGCTGG, TCACCTCT, TCACGCT, TCACTCAC, TCACTCAT, TCACTCTC, TCACTGAC, TCCACTCA, TCCCCTGA, TCCCTGAT, TCCTGCAG, TCTGCTAA, TGACCTCC, TGACCTCT, TGACCTGC, TGACCTGT, TGACGCC, TGACGCT, TGACTGAC, TGACTGAT, TGACTGCT, TGCTGAC, TGGCTGAC, TGGGCTGA, TGGGCTGG, TGGTCTGA, TGGTGACT, TGTACTGA, TGTCTGAC, TGTCTGAT, TGTGCTGA, TTACTGAC, TTACTGAT, TTCACTGA, TTCTGAC, TTGACTGA, TTGCTGAC, TTGCTGAT, TTTCTGAC, TTTCTGAT
A.IE.6	0.14	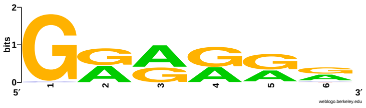	AAGAAAAG, AAGAAAGA, AAGGAAAG, AAGGAAGA, AGAAAAG, AGAAAAGA, AGAAAAGG, AGAAAGA, AGAAAGAA, AGAAAGAG, AGAAGAG, AGAAGGA, AGAAGGAA, AGAGAAG, AGAGAAGT, AGAGGAG, AGAGGGA, AGGAAAAG, AGGAAAG, AGGAAAGA, AGGAAAGG, AGGAAGA, AGGAAGAA, AGGAAGAG, AGGAAGGA, AGGAAGGG, AGGAGAG, AGGAGAGA, AGGAGAGG, AGGAGGA, AGGAGGAA, AGGAGGAG, AGGAGGGA, AGGAGGGG, AGGGAAAA, AGGGAAG, AGGGAAGA, AGGGAAGG, AGGGAGAA, AGGGAGAG, AGGGAGGA, AGGGAGGG, ATGAAGAA, ATGAGAG, ATGGAAG, CAGAAGGA, CAGAGAAG, CAGAGGAG, CAGAGGGA, CAGGAGAG, CAGGAGGA, CCGGGAG, CGAGAA, CGAGGA, CGGGAGG, CTGAGGCA, CTGGAAG, CTGGAGA, CTGGAGAA, CTGGAGG, CTGGAGGA, CTGGGAGG, GAAAAAAG, GAAAAAG, GAAAAAGA, GAAAAGAA, GAAAAGG, GAAAGAA, GAAAGAAA, GAAAGAG, GAAAGG, GAAAGGA, GAAAGGAA, GAAGAG, GAAGAGG, GAAGGA, GAAGGAA, GAAGGAAA, GAAGGCA, GAAGGG, GAAGGGA, GAGAAAGA, GAGAAAGG, GAGAAG, GAGAAGC, GAGAAGG, GAGAAGGA, GAGAAGGG, GAGAGA, GAGAGAA, GAGAGAAA, GAGAGAAG, GAGAGAGA, GAGAGG, GAGAGGA, GAGAGGAG, GAGAGGG, GAGAGGGA, GAGGAA, GAGGAAAA, GAGGAAGA, GAGGAAGG, GAGGAG, GAGGAGC, GAGGAGGA, GAGGAGGG, GAGGATC, GAGGGA, GAGGGAA, GAGGGCA, GAGGGGA, GAGGGGG, GGAAAAG, GGAAAGA, GGAAAGG, GGAACAG, GGAAGA, GGAAGAA, GGAAGAAA, GGAAGAG, GGAAGAGA, GGAAGAGG, GGAAGAT, GGAAGG, GGAAGGA, GGAAGGAA, GGAAGGAG, GGAAGGG, GGAAGGGA, GGAGAA, GGAGAAAA, GGAGAAAG, GGAGAAG, GGAGAAGA, GGAGAAGG, GGAGAGA, GGAGAGAG, GGAGAGC, GGAGAGG, GGAGAGGA, GGAGAGGG, GGAGATA, GGAGATG, GGAGGA, GGAGGAA, GGAGGAAG, GGAGGAC, GGAGGAG, GGAGGAGG, GGAGGAT, GGAGGCA, GGAGGCG, GGAGGG, GGAGGGA, GGAGGGAA, GGAGGGAG, GGAGGGC, GGAGGGG, GGAGGGGA, GGAGGGGG, GGGAAAG, GGGAAG, GGGAAGA, GGGAAGAG, GGGAAGG, GGGAAGGA, GGGAAGGG, GGGAAGT, GGGAGAA, GGGAGAAG, GGGAGAG, GGGAGAGA, GGGAGAGG, GGGAGCA, GGGAGG, GGGAGGA, GGGAGGAG, GGGAGGG, GGGAGGGA, GGGAGGGG, GGGAGGGT, GGGAGGT, GGGAGGTG, GGGGAGAG, GGGGAGGA, GGGGAGGG, GTGGAAG, GTGGAGA, GTGGGAGG, TCGAGA, TGAGGAA, TGAGGAAA, TGAGGAG, TGGAAGA, TGGAAGG, TGGAGAA, TGGAGAAG, TGGAGAG, TGGAGG, TGGAGGA, TGGAGGAG, TGGAGGG, TGGAGGTG, TGGGAAG, TGGGAAGA, TGGGAAGG, TGGGACA, TGGGAG, TGGGAGA, TGGGAGAG, TGGGAGG, TGGGAGGA, TGGGAGGC, TGGGAGGG, TTGGAAG, TTGGAGG, TTGGGA, TTGGGAAG, TTGGGAG, TTGGGAGG

A.IE.7	0.12	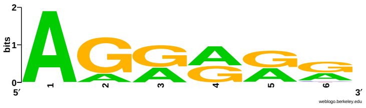	AAAAAAAG, AAAAAAGA, AAAAAGAG, AAAAGAAG, AAAAGAG, AAAAGAGA, AAAGAAAG, AAAGAAG, AAAGAGG, AAAGGAAG, AAAGGAG, AAGAAAG, AAGAAAGG, AAGAAGA, AAGAAGAA, AAGAAGC, AAGAAGG, AAGAAGGA, AAGAGAG, AAGAGAGA, AAGAGGA, AAGAGGAA, AAGAGGG, AAGGAAG, AAGGAAGG, AAGGAG, AAGGAGA, AAGGAGAA, AAGGAGAG, AAGGAGC, AAGGAGG, AAGGGAA, AAGGGAAA, AAGGGAG, ACAGAGA, ACAGAGAA, ACAGAGAG, ACAGAGG, AGAAAAAG, AGAAAGG, AGAAAGGA, AGAAATGG, AGAAGA, AGAAGAA, AGAAGAAA, AGAAGAAG, AGAAGAGA, AGAAGAGG, AGAAGG, AGAAGGAG, AGAAGGG, AGAAGGGA, AGAGAAAA, AGAGAAAG, AGAGAAGA, AGAGAAGG, AGAGACA, AGAGAG, AGAGAGA, AGAGAGAA, AGAGAGAG, AGAGAGG, AGAGAGGA, AGAGAGGG, AGAGGA, AGAGGAA, AGAGGAAA, AGAGGAAG, AGAGGAC, AGAGGAGA, AGAGGAGG, AGAGGG, AGAGGGAG, AGAGGGC, AGAGGGG, AGGAAG, AGGAAGG, AGGAGA, AGGAGAA, AGGAGAAG, AGGAGAT, AGGAGCA, AGGAGG, AGGAGGG, AGGAGGT, AGGAGGTG, AGGGAAA, AGGGAG, AGGGAGA, AGGGAGG, AGGGGAT, CAAGAAG, CAAGAGA, CAAGAGG, CAAGGAG, CAAGGGA, CAGAAAAA, CAGAAAG, CAGAAGA, CAGAAGG, CAGAGAA, CAGAGAAA, CAGAGAG, CAGAGAGA, CAGAGAGG, CAGAGGA, CAGAGGAA, CAGAGGG, CAGGAAG, CAGGAAGG, CAGGAGA, CAGGAGAA, CAGGAGG, CAGGAGGC, CAGGGAGA, CAGGGAGG, CAGGGGA, CCAGGAGA, CCAGGAGG, CCAGGGAG, CTAGAGA, GAAAGAGA, GAAGAAG, GAAGAAGA, GAAGAGA, GAAGAGAA, GAAGAGGA, GAAGGAAG, GAAGGAG, GAAGGAGA, GAAGGAGG, GAGAAAG, GAGAAGA, GAGAAGAA, GAGAGAG, GAGAGAGG, GAGGAAG, GAGGAGA, GAGGAGAA, GAGGAGG, GAGGGAG, GAGGGAGA, GAGGGAGG, GCAGAGAG, GCAGAGGG, GCAGGAGA, GCAGGAGG, GCAGGGAG, GGAGGAGA, GTAGAAG, TAAGGGA, TAGAAGA, TAGAAGC, TAGAAGG, TAGAGAAA, TAGAGAG, TAGAGG, TAGAGGA, TAGAGGG, TAGGAAG, TAGGAG, TAGGAGA, TAGGAGG, TAGGGA, TAGGGAA, TAGGGAG, TAGGGAT, TAGGGG, TAGGGGA, TGAGGAGG, TTAAGGG, TTAGAGG, TTAGGAG, TTAGGG, TTAGGGA, TTAGGGG
A.IE.8	0.057	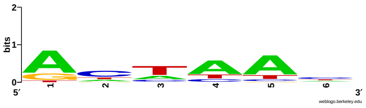	AAAACTAA, AAACTAAC, AAACTAAT, AAATTAAT, AACACTAA, AACTAAAA, AACTAAC, AACTAACA, AACTAAT, AACTAATG, AACTAATT, AACTTTTT, AATACTAA, AATACTGA, AATCTAAT, AATGCTAA, AATTAAC, AATTAACA, AATTAACT, ACAAAAT, ACACTAA, ACACTAAT, ACACTCAC, ACTAAATT, ACTAAC, ACTAACA, ACTAACAT, ACTAAT, ACTAATAT, ACTAATC, ACTAATG, ACTAATGA, ACTAATGT, ACTAATT, ACTAATTA, ACTAATTT, ACTTACAT, AGACTAG, AGCTAAC, AGTAACC, ATAACTAA, ATACTAA, ATACTAAT, ATACTTAA, ATACTTT, ATATTAAC, ATCTAAC, ATGACTAA, ATGCTAA, ATGCTAAC, ATGCTAAT, ATGTTAAC, ATTAACAT, ATTAACC, ATTAACT, ATTAACTG, ATTAACTT, ATTAATCT, ATTAATGA, ATTAATTT, ATTACTAA, ATTCTAAC, ATTCTAAT, CAAAAAAA, CACTAAC, CACTAACA, CACTAAT, CACTAATG, CAGCTAC, CATACTAA, CATTAAC, CATTAACA, CATTAACT, CATTAATG, CCAAAAAA, CGCTAA, CTATTAAT, CTGCTAAC, CTGCTAAT, CTTACTAA, CTTCTAAC, CTTCTAAT, CTTGTAAT, CTTTAACT, CTTTAATG, GAAACTAA, GAACTAA, GAACTAAT, GAACTAG, GAACTGG, GACTAAC, GACTAAT, GAGCTAG, GATTAAC, GCACTAA, GCTAATC, GCTAATGT, GGCTAAC, GTCTAAC, GTCTAAT, GTGCTAA, GTGCTAAC, GTGCTAAT, GTTAATAA, GTTACTAA, GTTCTAAT, GTTTTAAC, TAAAATAA, TAACTAAT, TAATAACT, TAATGCAT, TAATTAAC, TAATTAAT, TACTAAC, TACTAACA, TACTAAT, TACTAATG, TACTAATT, TAGCTAG, TATCTAAT, TATTAAC, TATTAACT, TATTAATT, TCAAAAAA, TCACTAA, TCACTAAC, TCACTAAT, TCAGTAAC, TCATTAAC, TCTTTAAC, TGAACTAA, TGACTAA, TGACTAAT, TGACTCTT, TGACTTGT, TGATTAAT, TGCTAAC, TGCTAAT, TGCTAATA, TGCTAATG, TGCTAATT, TGTCTAAC, TGTCTAAT, TGTTAATG, TGTTTAAC, TTAACTAA, TTAATAA, TTAATCTA, TTAATTAC, TTACTAAA, TTACTAAC, TTACTAAT, TTATAACA, TTATTAAC, TTCTAAC, TTCTAAT, TTGCTAA, TTGCTAAC, TTGCTAAT, TTGTAAC, TTGTTAAC, TTTAACTA, TTTAATAT, TTTAATTT, TTTACTAA, TTTATTAA, TTTCTAAC, TTTCTAAT, TTTTAACC, TTTTAACT, TTTTAATA, TTTTTAAC

A.IE.9	0.043	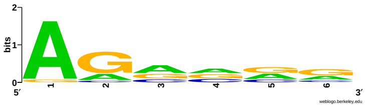	AAACAGG, AACCAGG, AACCCAGG, AAGACAG, AAGAGCA, AAGCAAG, AAGCAGA, AAGCAGG, AAGGCAG, AAGGGCA, ACACACAC, ACAGCCC, AGAAGCAG, AGACAGA, AGACAGG, AGACCAG, AGACGG, AGAGACAG, AGAGCAA, AGAGCAG, AGAGCCAG, AGAGGCAG, AGCAGAT, AGCAGGG, AGGACAG, CAACAAG, CAACAGA, CACACACA, CAGAGCAG, CAGCAGA, CTAGGCA, GAACAGG, GAAGCAG, GAAGCAGG, GACAGAG, GAGACAG, GAGAGCA, GAGCAAG, GAGCACA, GAGCAGGG, GAGCCACC, GAGCCAGG, GAGGCAG, GAGGCGG, GCAGGCAG, GGACAAG, GGAGCAG, GGAGCAGG, GGAGGCAG, TACAGGC, TACAGGG, TACCAGG, TAGAACA, TAGACAG, TAGAGGC, TAGCAAG, TAGCAGA, TAGCAGC, TAGGACA, TAGGCAG, TAGGCCA, TAGGGCA, TGACCGC, TGAGCCAC, TGAGGCAG, TTAGGCA
A.IE.10	0.032	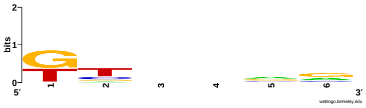	AATTCTAA, AGCCTAG, AGCTAGG, AGGCCTCA, AGGCTCAC, AGGCTGGG, AGTACAG, AGTAGCA, AGTCAGA, AGTCCAG, AGTCCCAG, AGTCTAG, AGTGCAG, AGTTCAG, ATACAGG, ATCCAGG, ATGCTCAC, ATGCTCAT, ATGTAGG, ATTACAGG, ATTAGCA, ATTCTCAC, ATTGCAG, ATTGCTAA, ATTGGCA, ATTGTAT, ATTTTAGA, CAGCTCAC, CATCTCAC, CATCTCAT, CCTGCAGA, CCTGCAGG, CCTTCTAA, CCTTCTGA, CGTTCTC, CTGCAGG, CTGCTCAC, CTGCTCAT, CTGTAGA, CTGTAGG, CTGTTCAC, CTTCCAG, CTTCCCAG, CTTCCTAA, CTTCTCAC, CTTGCAG, CTTTAGA, CTTTAGG, GATTACAG, GCTGCAGG, GGCTCAC, GGGCTCAC, GGTACAG, GGTCAGG, GGTCTCAC, GGTTGCA, GTACAGA, GTAGCAG, GTCAGGA, GTCAGGG, GTCCAGA, GTCTCAC, GTCTCATT, GTGCAGG, GTGCTCAC, GTGTCTAA, GTTCTCAC, GTTGCAG, GTTTCTAA, TACAGG, TATGCAG, TATGTAG, TATTCTAA, TCTCCAGG, TCTGTAG, TGCTAGG, TGCTCAC, TGGCCAGG, TGGCTCAC, TGGCTCAT, TGGTGCA, TGTACTCA, TGTCAGG, TGTCCTAA, TGTCTAA, TGTCTCAC, TGTCTCAT, TGTCTTAC, TGTGCTAA, TGTTCTAA, TTACAGG, TTCCAGA, TTCCAGG, TTCCTAG, TTCTAGG, TTGCAGG, TTGCCAG, TTGCTCAC, TTGGCAG, TTGTAGA, TTGTAGG, TTGTATT, TTGTATTT, TTGTCTAA, TTTAGAG, TTTAGGG, TTTCCCAG, TTTCCTAA, TTTCCTGG, TTTGCTAA, TTTGGAG, TTTGGAGA, TTTGGGA, TTTTAGG, TTTTCCAG, TTTTCTAA, TTTTGACC
A.IE.11	0.052	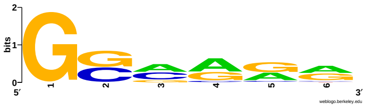	AGCAAAG, AGCAAGA, AGCAAGG, AGCAGAG, AGCAGAGA, AGCAGAGG, AGCAGCAG, AGCAGGA, AGCATAG, AGGCAAA, AGGCAAG, AGGCAGA, AGGCAGAG, AGGCAGG, AGGCAGGA, AGGGCAGG, AGGGGCAG, CAGCAGCA, CTGCAGAG, CTGCAGGA, CTGCAGGG, CTGCAGGT, CTGGGCAG, GAGGCAGA, GAGGCAGG, GAGGGCAG, GCAAAGA, GCAAAGG, GCAAGAA, GCAAGAG, GCAGAAG, GCAGAGA, GCAGAGG, GCAGGA, GCAGGAA, GCAGGAG, GCCAGAA, GCGAGA, GGAGCAA, GGCAAG, GGCAAGA, GGCAAGG, GGCAGA, GGCAGAA, GGCAGAC, GGCAGAG, GGCAGAGA, GGCAGAGG, GGCAGGA, GGCAGGAG, GGCATGG, GGCGAGG, GGCGGGGG, GGGCAAG, GGGCAGAG, GGGCAGGG, GGGCCGGG, GGGCGGGC, GGGCGGGG, GGGGCAA, GGGGCAGG, GGGGGCAG, TGCAAGG, TGCAGAG, TGCAGGA, TGCAGGG, TGCAGGTG, TGGAGCA, TGGAGCAG, TGGCAAG, TGGCAGA, TGGCAGAG, TGGGCAA, TGGGCAGG, TGGGGCAG, TTGGGCA

A.IE.12	0.19	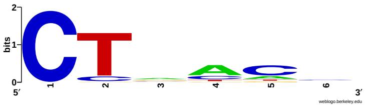	AACTAACT, ACCCTCAC, ACCCTCTC, ACCCTGAC, ACCTAAC, ACCTCACC, ACCTCACT, ACCTGAC, ACCTGACC, ACCTGACT, ACTAACC, ACTAACCT, ACTAACG, ACTAACT, ACTAACTT, ACTCACC, ACTCACCC, ACTCACCT, ACTCACTC, ACTCTAAT, ACTCTGAC, ACTGAC, ACTGACAT, ACTGACC, ACTGACCA, ACTGACCC, ACTGACCT, ACTGACG, ACTGACT, ACTGACTC, ACTGACTG, ACTGACTT, ACTGATC, ACTGATTT, AGCCCCTC, AGCCTCAC, AGCCTGAC, AGCTCACC, AGCTCACT, AGCTCTGG, AGCTGACC, AGCTGACT, ATCCACC, ATCTAATG, CACCCCTC, CACCTCAC, CACCTGAC, CACTCAC, CACTCACC, CACTCACT, CACTCCAG, CACTGACA, CACTGACC, CACTGACT, CACTGATG, CACTGCAC, CCACTCAC, CCCAACCT, CCCACTCA, CCCCACTC, CCCCACTT, CCCCCCTC, CCCCCTCA, CCCCTCAC, CCCCTGAC, CCCTAAC, CCCTAACC, CCCTCAC, CCCTCACA, CCCTCACC, CCCTCACT, CCCTCTCA, CCCTCTGA, CCCTGAC, CCCTGACA, CCCTGACC, CCCTGACT, CCCTGATC, CCCTGATG, CCTAAC, CCTAACA, CCTAACC, CCTAACCC, CCTAACG, CCTAACT, CCTAACTG, CCTAACTT, CCTAATC, CCTAATG, CCTCAC, CCTCACC, CCTCACCA, CCTCACCC, CCTCACCT, CCTCACG, CCTCACT, CCTCACTC, CCTCACTG, CCTCACTT, CCTCCCAA, CCTCCTAA, CCTCTAA, CCTCTAAC, CCTCTAAT, CCTCTCAC, CCTCTCAT, CCTCTGAC, CCTCTGAT, CCTGAC, CCTGACAC, CCTGACC, CCTGACCA, CCTGACCC, CCTGACG, CCTGACT, CCTGACTC, CCTGACTG, CCTTGACC, CCTTGACT, CCTTTTTT, CGAACT, CGCTGACC, CTAAACTT, CTAAC, CTAACA, CTAACAC, CTAACAG, CTAACAT, CTAACATG, CTAACATT, CTAACC, CTAACCA, CTAACCAT, CTAACCC, CTAACCCT, CTAACCT, CTAACCTC, CTAACCTG, CTAACCTT, CTAACG, CTAACGC, CTAACGT, CTAACT, CTAACTC, CTAACTCT, CTAACTG, CTAACTGA, CTAACTGT, CTAACTT, CTAACTTT, CTAATAAT, CTAATATT, CTAATCTT, CTAATG, CTAATGAT, CTAATGC, CTAATGCT, CTAATGT, CTAATGTG, CTAATGTT, CTAATTCT, CTAATTGT, CTACAGG, CTACGA, CTCACACC, CTCACC, CTCACCAC, CTCACCAT, CTCACCC, CTCACCCC, CTCACCCT, CTCACCG, CTCACCT, CTCACCTC, CTCACCTG, CTCACCTT, CTCACG, CTCACGC, CTCACGT, CTCACTC, CTCACTCA, CTCACTCC, CTCACTCT, CTCACTGC, CTCACTTG, CTCATGCC, CTCATGTG, CTCATGTT, CTCATTCC, CTCCCAAA, CTCCCAGG, CTCCTAAC, CTCCTCAC, CTCCTGAT, CTCTAAAT, CTCTAAC, CTCTAACC, CTCTAACT, CTCTAAT, CTCTAATG, CTCTAATT, CTCTCAC, CTCTCACC, CTCTGAC, CTCTGACC, CTCTGACT, CTCTGATC, CTCTGATG, CTGAC, CTGACACC, CTGACACT, CTGACATC, CTGACATG, CTGACATT, CTGACC, CTGACCA, CTGACCAC, CTGACCAG, CTGACCAT, CTGACCC, CTGACCCA, CTGACCCC, CTGACCCG, CTGACCCT, CTGACCG, CTGACCT, CTGACCTC, CTGACCTG, CTGACCTT, CTGACG, CTGACGC, CTGACGG, CTGACGT, CTGACTCA, CTGACTCT, CTGACTG, CTGACTGA, CTGACTGC, CTGACTGT, CTGACTTG, CTGACTTT, CTGATCCC, CTGATCTC, CTGATCTT, CTGATGCC, CTGATGCT, CTGATGTG, CTGATTCT, CTGCACTC, CTTATTCA, CTTGACTC, GACCCCTC, GACCTCTC, GACCTGAC, GACTGACC, GACTGACT, GCCCCCCT, GCCCTCAC, GCCCTGAC, GCCTAAC, GCCTCAC, GCCTCACA, GCCTCACC, GCCTCACT, GCCTCTCA, GCCTCTGA, GCCTGAC, GCCTGACC, GCCTGACT, GCTAAC, GCTAACA, GCTAACC, GCTAACG, GCTAACT, GCTCACAC, GCTCACC, GCTCACCC, GCTCACCT, GCTCACG, GCTCACT, GCTCACTC, GCTCACTG, GCTCACTT, GCTCATGT, GCTCTAA, GCTCTAAC, GCTCTGAC, GCTCTGAT, GCTGAC, GCTGACA, GCTGACAC, GCTGACAT, GCTGACC, GCTGACCA, GCTGACCC, GCTGACCT, GCTGACG, GCTGACT, GCTGACTC, GCTGACTG, GCTGACTT, GCTGATTT, GCTTACC, GGCCTCAC, GGCCTGAC, GGCTCACC, GGCTCACT, GGCTCATG, GGCTCTGA, GGCTGACC, GGCTGACT, GTCCTCAC, GTCCTGAC, GTCTCACC, GTCTCACT, GTCTGACC, GTCTGACT, TACTCAC, TACTGACT, TACTGAT, TACTGATG, TCACCCTC, TCCCACTC, TCCCCCCC, TCCCTAAC, TCCCTCAC, TCCCTGAC, TCCTAAC, TCCTAACA, TCCTAACC, TCCTAACT, TCCTAAT, TCCTAATG, TCCTAATT, TCCTCAC, TCCTCACC, TCCTCACT, TCCTCTAA, TCCTGAC, TCCTGACA, TCCTGACT, TCCTGATG, TCGAAC, TCTAAATG, TCTAAC, TCTAACA, TCTAACAT, TCTAACC, TCTAACCA, TCTAACCC, TCTAACCT, TCTAACT, TCTAACTC, TCTAACTG, TCTAACTT, TCTAATA, TCTAATAA, TCTAATAT, TCTAATCT, TCTAATG, TCTAATGA, TCTAATGT, TCTAATTG, TCTCACC, TCTCACCA, TCTCACCC, TCTCACCT, TCTCACG, TCTCACTG, TCTCCTAA, TCTCTAAC, TCTCTAAT, TCTCTGAC, TCTCTGAT, TCTGAC, TCTGACC, TCTGACCA, TCTGACCC, TCTGACCT, TCTGACG, TCTGACT, TCTGACTC, TCTGACTG, TCTGACTT, TCTGATGC, TCTGATTC, TCTGATTG, TCTTAACT, TCTTACCT, TCTTGACT, TGACCCCT, TGACCCT, TGACCCTC, TGACCCTT, TGCCTCAC, TGCCTGAC, TGCTAACA, TGCTAACC, TGCTAACT, TGCTCACC, TGCTCACT, TGCTCATG, TGCTGACA, TGCTGACC, TGCTGACT, TGCTGATC

			TTCCTAAC, TTCCTAAT, TTCCTGAC, TTCTAACA, TTCTAACC, TTCTAACT, TTCTAATA, TTCTAATC, TTCTAATG, TTCTAATT, TTCTCACT, TTCTGACC, TTCTGACT, TTCTGATC, TTCTTAAC, TTCTTACC
A.IE.13	0.021	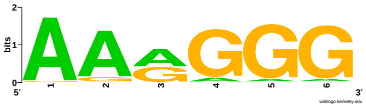	AAAAAAGG, AAAAAGGA, AAAAGGG, AAAGGG, AAAGGGA, AAAGGGAA, AAAGGGG, AAGGGG, AAGGGGA, AAGGGGG, AGAAAGGG, CAAAAGG, CAAAGGG, GAAAAGGA, GAAAGGG, GAAGGGG, GGAAGGGG
A.IE.14	0.048	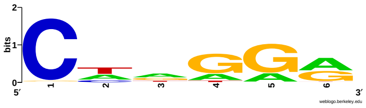	ACAAGGA, ACAAGGG, ACAGGGA, ACATAGG, ACCAAGG, ACCAGGA, ACTAGGA, ACTGGGA, AGCCAGGA, AGCTAGA, AGCTGGAG, AGCTGGGA, AGCTGGGG, CAAGGA, CAAGGAA, CACAGAGA, CAGGGAA, CAGGGAAG, CCAAGAA, CCAAGAG, CCAAGGA, CCAGGAG, CCAGGAGT, CCATAGA, CCATAGG, CCCAAAG, CCCAGCTA, CCCAGGAG, CCCAGGGA, CCCTAGA, CCTAGGA, CCTAGGG, CCTGGAGA, CCTGGAGG, CCTGGGAG, CCTGGGCA, CCTGGGGG, CTAGAGG, CTAGGAA, CTAGGAG, CTAGGAT, CTAGGG, CTAGGGA, CTATAGA, CTGGGAA, CTGGGAAG, CTGGGAG, CTGGGATT, CTTGGAG, CTTGGGA, CTTGGGAG, CTTGGGG, GACAGAGA, GCAAGGA, GCAAGGG, GCACAGAG, GCAGGGA, GCATAGA, GCCAAGA, GCCAGGA, GCCAGGAG, GCCCAGAG, GCCCAGGA, GCCTGGAG, GCCTGGGG, GCTAGGA, GCTAGGG, GCTGGAGG, GCTGGGA, GCTGGGAT, GCTGGGGA, GCTGGGGG, GCTTGGG, GGCAGGGA, GGCCGGGG, GGCTGGGA, TCAAGGG, TCAGGGG, TCCAAGA, TCCAAGG, TCCAGAG, TCCAGGA, TCCCAAAG, TCCCAGAG, TCCCAGGG, TCCCCAGG, TCCTAGG, TCCTGGAG, TCCTGGGA, TCCTGGGG, TCTAGAG, TCTAGGG, TCTGGAG, TGCTGGGA, TTCACTAA
A.IE.15	0.12	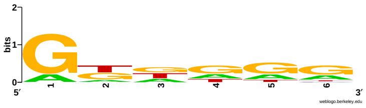	AAAGTGG, AAGGTAG, AAGTAGA, AAGTAGG, AAGTGGG, AGAGTAG, AGAGTGG, AGGGTAG, AGGGTGGG, AGGTAAG, AGGTAG, AGGTAGA, AGGTAGC, AGGTAGG, AGGTATG, AGGTGAG, AGGTGAGG, AGGTGGA, AGGTGGAG, AGGTGGG, AGGTGGGA, AGGTGGGG, AGGTTAG, AGGTTGC, AGGTTGG, AGTAGAA, AGTAGAG, AGTAGG, AGTAGGA, AGTAGGG, AGTATAG, AGTGAGG, AGTGGAG, AGTGGGA, AGTGGGG, AGTGTAG, AGTTGGA, AGTTGGG, CAAGTAG, CAGGGTA, CAGGTGGG, CGGGGTGG, CGGTGAC, CTGGGTGG, CTGGTGAC, CTGTGGGA, CTGTGTGG, GAAGTAG, GAAGTGG, GAGGTAG, GAGGTGG, GAGGTGGG, GAGGTTG, GAGTACA, GAGTAG, GAGTAGA, GAGTAGG, GAGTGGG, GAGTTGG, GCAGGTGG, GCAGTAG, GCAGTGAG, GGAGGTGG, GGAGTAG, GGAGTGG, GGGGGTGG, GGGGTGAC, GGGGTGGC, GGGGTGGG, GGGTAGG, GGGTAGGG, GGGTGGA, GGGTGGG, GGGTGGGC, GGGTGGGG, GGGTTAG, GGGTTGG, GGTAAGG, GGTAGAG, GGTAGG, GGTAGGA, GGTAGGG, GGTAGGT, GGTATGG, GGTGAAG, GGTGACCT, GGTGAGGG, GGTGGAG, GGTGGAGG, GGTGGGA, GGTGGGAG, GGTGGGG, GGTGGGGA, GGTGGGGG, GGTGGTGG, GGTGTGG, GGTTAAG, GGTTAGG, GGTTGGG, GGTTTAG, GTAAGGG, GTAGAG, GTAGAGA, GTAGAGC, GTAGAGG, GTAGGA, GTAGGAA, GTAGGAG, GTAGGCA, GTAGGG, GTAGGGA, GTAGGGG, GTGAGCCA, GTGAGGA, GTGAGGG, GTGGAGG, GTGGGA, GTGGGAG, GTGGGGA, GTGGGGG, GTGGGGGC, GTGGGTGG, GTGGTAG, GTGGTGG, GTGTAAC, GTGTAATT, GTGTAGA, GTGTAGG, GTGTGGGG, GTTAGG, GTTAGGA, GTTGGAG, GTTGGGA, GTTGGGG, GTTGTAG, GTTTAATG, GTTTAGG, TAGGTGC, TAGGTGG, TAGTACA, TAGTAGA, TAGTGGG, TAGTTGG, TCGTAG, TGGGGTGG, TGGGTGGG, TGGTAGA, TGGTAGG, TGTAGAG, TGTAGG, TGTAGGA, TGTAGGC, TGTAGGG, TGTAGGT, TGTGGGA, TGTGTAAC, TGTGTAG, TGTGTGGG, TGTTAGG, TGTTGACT, TGTTTAAT, TTAGTAG, TTAGTGG, TTGGTAG

1.3 5′SS ESEs/ESSs

D.EE.1	0.37	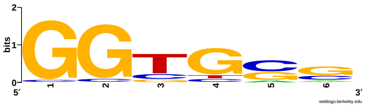	ACGGGCG, AGGTGGTG, CCGCCGC, GCAGTGGG, GGCCGCG, GGCGGAC, GGGCAGCT, GGGTGGG, GGTGACG, GGTGCG, GGTGCGG, GGTGGGC, GGTTGAC, TGGTGGGC
D.EE.2	0.21	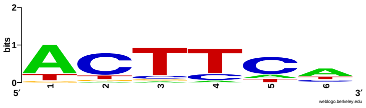	ACTTCA, CTGGTCTC, GGTCTCCC, TGGTCTC, TGGTCTCA, TGGTCTCC, TGGTCTCT, TTACTTA
D.EE.3	0.086	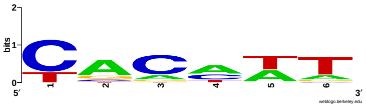	AACAGATT, CACATTA, CACCTTA, CACTGTGA, CTCACTGT, CTCATTGT, TGCAATA
D.EE.4	0.076	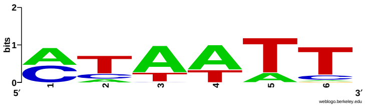	AAATATTT, ACTATTT, ATAAACT, CTAATTT
D.EE.5	0.26	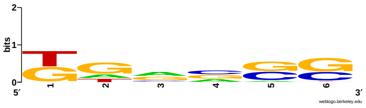	AAGAAGGT, ATGAACG, ATGAGCTG, CTGACCCT, CTGACTGT, GAAGGAGG, GACCCTGC, GAGCGCC, GATGGAGG, GGACCCT, GGACCCTG, GGGCCCTG, GGTGAGG, GTGACGG, GTGAGCC, GTGGCGG, TGACGGG, TGAGCGG, TGGACCCT, TGGATCCT, TGTGGTGG

1.4 3′SS ESEs/ESSs

A.EE.1	0.14	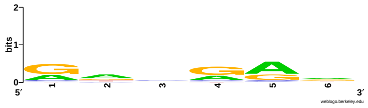	AACGAAT, AACGAATG, AACGAGT, AACGAGTG, AATGAATG, AATGAGTG, AGTGACCT, ATTTTGGA, CAGTGACC, CCCGTGAG, CCGTGAGC, CGACGAGT, CGATGAGT, CGTGAGCT, GACGAAT, GACGAATG, GACGAGT, GACGAGTG, GAGGGAGA, GATGAATG, GATGAGT, GATGAGTG, GGACGAGT, GGATGAAT, GGATGAGT, GGTCATCA, GGTGACAT, GTCATCAC, TAAATGAA, TAGATGAA, TCAGTGAC, TCGACGA, TCGACGAG, TCGATGAG, TCGGTGA, TGACGAGT, TGATGAAT, TGATGAGT, TGGATCCT, TTGACGA, TTGACGAG, TTGATGAA, TTGATGAG, TTGGTGAC
A.EE.2	0.22	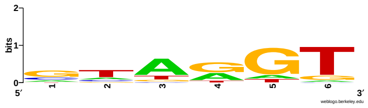	ACAGGTA, CCAGGTA, GTAAGT, TACAGGT, TCAGTGGT, TCCACAGG, TGTACAAA, TTCCAGAG
A.EE.3	0.47	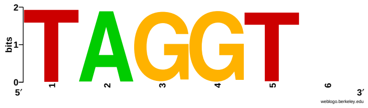	CTAGGT, TAGGT, TAGGTA, TAGGTC, TAGGTT
A.EE.4	0.12	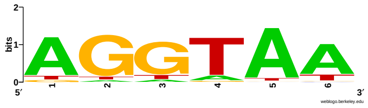	AGGTAA, AGGTCATC, CAGGTAT, TGTTTAA, TTTATAA, TTTTTCAG
A.EE.5	0.055	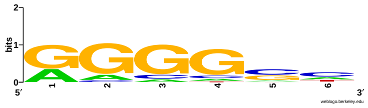	AGGGCTA, GACCCTGC, GACCCTTC, GAGGCCTT, GAGGGGC, GGACCCCG, GGACCCTG, GGGGCAA, GGGGCCCC, GGGGGGC, TGGACCCT, TGGACTCT

2 Statistical significance of the elements found

Table 1: Statistical significance of the elements found

Element	Count next to SS	Background count	Chi-square test	LOD
**3′SS ESE**				
AACGAAT	87	16	1.72×10^{-70}	2.44
AACGAATG	67	6	6.87×10^{-137}	3.48
AACGAGT	115	29	2.07×10^{-57}	1.99
AACGAGTG	93	5	0.00	4.22

Continued on next page...

Table 1 – continued from previous page

Element	Count next to SS	Background count	Chi-square test	LOD
AATGAATG	127	23	2.81×10^{-104}	2.47
AATGAGTG	118	20	1.93×10^{-106}	2.56
ACAGGTA	68	19	2.55×10^{-29}	1.84
AGGTAA	120	37	2.16×10^{-42}	1.70
AGTGACCT	76	19	4.47×10^{-39}	2.00
ATTTTGGA	109	35	6.72×10^{-36}	1.64
CAGGTAT	67	12	9.12×10^{-57}	2.48
CAGTGACC	91	28	1.10×10^{-32}	1.70
CCAGGTA	88	20	3.26×10^{-52}	2.14
CCCGTGAG	64	11	1.76×10^{-57}	2.54
CCGTGAGC	68	14	3.25×10^{-47}	2.28
CGACGAGT	66	5	7.31×10^{-164}	3.72
CGATGAGT	66	14	6.55×10^{-44}	2.24
CGTGAGCT	77	10	1.25×10^{-99}	2.94
CTAGGT	102	24	4.48×10^{-57}	2.09
GACGAAT	107	23	1.10×10^{-68}	2.22
GACGAATG	89	4	0.00	4.48
GACGAGT	185	39	7.01×10^{-121}	2.25
GACGAGTG	163	15	0.00	3.44
GATGAATG	184	26	8.19×10^{-211}	2.82
GATGAGT	248	79	1.30×10^{-80}	1.65
GATGAGTG	194	23	1.89×10^{-278}	3.08
GGACGAGT	85	15	5.11×10^{-73}	2.50
GGATGAAT	82	18	2.03×10^{-51}	2.19
GGATGAGT	83	17	1.13×10^{-57}	2.29
GGTGACAT	74	14	7.19×10^{-58}	2.40
GTAAGT	92	28	1.12×10^{-33}	1.72
TAAATGAA	76	25	1.98×10^{-24}	1.60
TACAGGT	74	14	7.19×10^{-58}	2.40
TAGATGAA	68	13	1.54×10^{-52}	2.39
TAGGT	353	108	6.85×10^{-123}	1.71
TAGGTA	64	8	3.04×10^{-87}	3.00
TAGGTC	115	35	1.15×10^{-41}	1.72
TAGGTT	87	21	4.99×10^{-47}	2.05
TCAGTGAC	86	27	7.03×10^{-30}	1.67
TCAGTGGT	77	21	2.42×10^{-34}	1.87
TCCACAGG	64	21	6.39×10^{-21}	1.61
TCGACGA	95	24	1.34×10^{-47}	1.98
TCGACGAG	64	6	6.02×10^{-124}	3.42
TCGATGAG	68	11	3.37×10^{-66}	2.63
TCGGTGA	71	22	1.51×10^{-25}	1.69
TGACGAGT	64	6	6.02×10^{-124}	3.42

Continued on next page...

Table 1 – continued from previous page

Element	Count next to SS	Background count	Chi-square test	LOD
TGATGAAT	143	25	3.84×10^{-123}	2.52
TGATGAGT	120	29	4.62×10^{-64}	2.05
TGTACAAA	68	20	7.11×10^{-27}	1.77
TGTTTAA	106	33	5.35×10^{-37}	1.68
TTCCAGAG	83	26	5.19×10^{-29}	1.67
TTGACGA	117	31	8.01×10^{-54}	1.92
TTGACGAG	64	14	9.93×10^{-41}	2.19
TTGATGAA	164	45	2.07×10^{-70}	1.87
TTGATGAG	125	40	3.53×10^{-41}	1.64
TTGGTGAC	69	11	1.78×10^{-68}	2.65
TTTATAA	80	25	3.82×10^{-28}	1.68
TTTTTCAG	64	16	3.55×10^{-33}	2.00
**3′SS ESS**				
AGGGCTA	21	69	7.53×10^{-09}	-1.72
AGGTCATC	20	71	1.42×10^{-09}	-1.83
GACCCTGC	29	169	4.79×10^{-27}	-2.54
GACCCTTC	19	63	2.96×10^{-08}	-1.73
GAGGCCTT	17	63	6.81×10^{-09}	-1.89
GAGGGAGA	26	78	3.91×10^{-09}	-1.58
GAGGGGC	33	107	8.43×10^{-13}	-1.70
GGACCCCG	21	64	7.66×10^{-08}	-1.61
GGACCCTG	44	186	2.18×10^{-25}	-2.08
GGGGCAA	24	73	9.75×10^{-09}	-1.60
GGGGCCCC	22	67	3.85×10^{-08}	-1.61
GGGGGGC	18	63	1.43×10^{-08}	-1.81
GGTCATCA	23	73	4.85×10^{-09}	-1.67
GTCATCAC	18	65	5.55×10^{-09}	-1.85
TGGACCCT	34	169	2.90×10^{-25}	-2.31
TGGACTCT	23	76	1.20×10^{-09}	-1.72
TGGATCCT	25	76	4.91×10^{-09}	-1.60
**5′SS ESE**				
AAATATTT	77	25	2.48×10^{-25}	1.62
AACAGATT	66	19	4.16×10^{-27}	1.80
ACTATTT	83	26	5.19×10^{-29}	1.67
ACTTCA	65	18	1.60×10^{-28}	1.85
ATAAACT	94	23	1.37×10^{-49}	2.03
CACATTA	71	21	1.02×10^{-27}	1.76
CACCTTA	95	29	1.56×10^{-34}	1.71
CACTGTGA	107	27	1.74×10^{-53}	1.99
CTAATTT	76	23	2.16×10^{-28}	1.72
CTCACTGT	95	21	1.17×10^{-58}	2.18
CTCATTGT	64	12	6.21×10^{-51}	2.42

Continued on next page...

Table 1 – continued from previous page

Element	Count next to SS	Background count	Chi-square test	LOD
CTGACTGT	69	21	1.13×10^{-25}	1.72
CTGGTCTC	120	20	9.49×10^{-111}	2.58
GGTCTCCC	65	18	1.60×10^{-28}	1.85
TGCAATA	74	24	1.86×10^{-24}	1.62
TGGTCTC	220	64	1.09×10^{-84}	1.78
TGGTCTCA	72	15	4.99×10^{-49}	2.26
TGGTCTCC	78	24	2.97×10^{-28}	1.70
TGGTCTCT	72	15	4.99×10^{-49}	2.26
TTACTTA	71	22	1.51×10^{-25}	1.69
**5′SS ESS**				
AAGAAGGT	21	65	4.83×10^{-08}	-1.63
ACGGGCG	21	66	3.04×10^{-08}	-1.65
AGGTGGTG	18	63	1.43×10^{-08}	-1.81
ATGAACG	32	98	2.61×10^{-11}	-1.61
ATGAGCTG	22	68	2.43×10^{-08}	-1.63
CCGCCGC	23	76	1.20×10^{-09}	-1.72
CTGACCCT	21	64	7.66×10^{-08}	-1.61
GAAGGAGG	27	86	1.99×10^{-10}	-1.67
GACCCTGC	29	190	1.60×10^{-31}	-2.71
GAGCGCC	19	69	1.75×10^{-09}	-1.86
GATGGAGG	17	84	2.66×10^{-13}	-2.30
GCAGTGGG	21	75	4.50×10^{-10}	-1.84
GGACCCT	69	267	8.47×10^{-34}	-1.95
GGACCCTG	28	179	1.53×10^{-29}	-2.68
GGCCGCG	23	76	1.20×10^{-09}	-1.72
GGCGGAC	19	67	4.51×10^{-09}	-1.82
GGGCAGCT	23	75	1.92×10^{-09}	-1.71
GGGCCCTG	25	90	7.30×10^{-12}	-1.85
GGGTGGG	12	69	6.79×10^{-12}	-2.52
GGTGACG	21	77	1.75×10^{-10}	-1.87
GGTGAGG	32	96	6.49×10^{-11}	-1.58
GGTGCG	58	203	2.50×10^{-24}	-1.81
GGTGCGG	16	86	4.41×10^{-14}	-2.43
GGTGGGC	27	100	2.87×10^{-13}	-1.89
GGTTGAC	21	64	7.66×10^{-08}	-1.61
GTGACGG	24	72	1.54×10^{-08}	-1.58
GTGAGCC	23	72	7.70×10^{-09}	-1.65
GTGGCGG	27	82	1.25×10^{-09}	-1.60
TGACGGG	17	65	2.62×10^{-09}	-1.93
TGAGCGG	20	75	2.14×10^{-10}	-1.91
TGGACCCT	16	180	2.31×10^{-34}	-3.49
TGGATCCT	20	75	2.14×10^{-10}	-1.91

Continued on next page...

Table 1 – continued from previous page

Element	Count next to SS	Background count	Chi-square test	LOD
TGGTGGGC	22	69	1.53×10^{-08}	-1.65
TGTGGTGG	15	66	3.43×10^{-10}	-2.14
**5′SS ISE**				
ACCCTCCT	76	22	1.14×10^{-30}	1.79
ACCGCCC	82	21	1.99×10^{-40}	1.97
ACCGCG	109	34	7.32×10^{-38}	1.68
ACCGGCC	76	18	1.52×10^{-42}	2.08
ACCGGGC	86	25	3.11×10^{-34}	1.78
ACCGGGG	108	33	5.88×10^{-39}	1.71
ACGGGGC	125	33	1.00×10^{-57}	1.92
ACTGGGCC	68	21	1.11×10^{-24}	1.70
ACTGGGGA	91	28	1.10×10^{-32}	1.70
ACTTTAT	82	21	1.99×10^{-40}	1.97
ACTTTTTT	80	22	4.01×10^{-35}	1.86
AGGCCGGG	80	20	4.84×10^{-41}	2.00
AGGCGGGG	73	17	5.12×10^{-42}	2.10
AGGGCCGG	67	13	1.04×10^{-50}	2.37
AGGGCGG	147	47	3.42×10^{-48}	1.65
AGGGCGGG	80	18	2.30×10^{-48}	2.15
AGGGGCGG	73	20	2.12×10^{-32}	1.87
AGGGGGCC	74	21	6.16×10^{-31}	1.82
AGGGGGGC	66	18	1.12×10^{-29}	1.87
AGGGGGTG	111	33	5.40×10^{-42}	1.75
ATATTTTT	123	31	2.47×10^{-61}	1.99
ATCATTTT	64	16	3.55×10^{-33}	2.00
ATCCTTT	79	19	4.14×10^{-43}	2.06
ATGGGCAG	93	28	1.11×10^{-34}	1.73
ATGGGGGG	67	20	7.81×10^{-26}	1.74
ATTATTA	76	24	2.55×10^{-26}	1.66
ATTTATT	212	67	3.22×10^{-70}	1.66
ATTTATTT	133	37	4.11×10^{-56}	1.85
ATTTTATT	112	35	9.99×10^{-39}	1.68
ATTTTCAT	64	18	2.17×10^{-27}	1.83
ATTTTGTT	82	26	4.64×10^{-28}	1.66
ATTTTTTT	245	71	9.70×10^{-95}	1.79
CACCGGG	90	28	1.04×10^{-31}	1.68
CAGCCGG	97	31	2.05×10^{-32}	1.65
CAGGCGGG	72	14	3.40×10^{-54}	2.36
CAGGGCCG	64	18	2.17×10^{-27}	1.83
CCAGGCGG	65	21	7.87×10^{-22}	1.63
CCAGGGGC	140	43	1.64×10^{-49}	1.70
CCCATGGG	72	23	1.66×10^{-24}	1.65

Continued on next page...

Table 1 – continued from previous page

Element	Count next to SS	Background count	Chi-square test	LOD
CCCCCCGC	79	20	9.65×10^{-40}	1.98
CCCCCGC	224	64	5.48×10^{-89}	1.81
CCCCCGCC	127	30	3.52×10^{-70}	2.08
CCCCCGGC	71	21	1.02×10^{-27}	1.76
CCCCCGGG	104	25	3.11×10^{-56}	2.06
CCCCGCC	344	108	3.60×10^{-114}	1.67
CCCCGCCC	214	59	1.48×10^{-90}	1.86
CCCCGCG	96	20	9.07×10^{-65}	2.26
CCCCGGCC	85	22	3.94×10^{-41}	1.95
CCCCGGG	336	86	4.51×10^{-160}	1.97
CCCCGGGC	107	18	1.05×10^{-97}	2.57
CCCCGGGG	131	29	5.24×10^{-80}	2.18
CCCGACC	76	24	2.55×10^{-26}	1.66
CCCGCC	731	217	9.10×10^{-267}	1.75
CCCGCCC	402	94	1.77×10^{-221}	2.10
CCCGCCCC	201	51	5.97×10^{-98}	1.98
CCCGCCCG	69	10	1.10×10^{-77}	2.79
CCCGCCCT	90	20	3.19×10^{-55}	2.17
CCCGCCG	116	23	9.03×10^{-84}	2.33
CCCGCCT	142	43	1.68×10^{-51}	1.72
CCCGCG	266	61	7.55×10^{-152}	2.12
CCCGCGC	87	14	9.01×10^{-85}	2.64
CCCGCGG	126	23	2.55×10^{-102}	2.45
CCCGCTT	68	21	1.11×10^{-24}	1.70
CCCGGCC	270	73	1.24×10^{-117}	1.89
CCCGGCCC	118	26	8.99×10^{-73}	2.18
CCCGGCG	87	20	9.67×10^{-51}	2.12
CCCGGG	922	287	1.61×10^{-307}	1.68
CCCGGGC	264	59	6.30×10^{-157}	2.16
CCCGGGCA	74	20	1.43×10^{-33}	1.89
CCCGGGCC	121	21	1.44×10^{-105}	2.53
CCCGGGG	401	77	1.92×10^{-298}	2.38
CCCGGGGA	78	17	1.58×10^{-49}	2.20
CCCGGGGC	147	21	1.98×10^{-166}	2.81
CCCGGGGG	124	23	1.85×10^{-98}	2.43
CCCTCCCG	64	18	2.17×10^{-27}	1.83
CCCTGCCG	66	18	1.12×10^{-29}	1.87
CCGCCC	734	208	2.95×10^{-291}	1.82
CCGCCCC	337	89	2.60×10^{-152}	1.92
CCGCCCCC	123	33	2.54×10^{-55}	1.90
CCGCCCCG	80	12	8.58×10^{-86}	2.74
CCGCCCCT	69	21	1.13×10^{-25}	1.72

Continued on next page...

Table 1 – continued from previous page

Element	Count next to SS	Background count	Chi-square test	LOD
CCGCCCG	132	18	4.90×10^{-159}	2.87
CCGCCCGC	65	5	1.34×10^{-158}	3.70
CCGCCCTG	67	15	4.24×10^{-41}	2.16
CCGCCG	263	72	3.32×10^{-112}	1.87
CCGCCGC	110	21	5.08×10^{-84}	2.39
CCGCCGG	88	18	3.72×10^{-61}	2.29
CCGCG	660	194	1.95×10^{-245}	1.77
CCGCGC	220	51	8.27×10^{-124}	2.11
CCGCGCC	77	16	1.65×10^{-52}	2.27
CCGCGG	299	68	1.12×10^{-172}	2.14
CCGCGGC	108	21	2.27×10^{-80}	2.36
CCGCGGCC	65	10	9.39×10^{-68}	2.70
CCGCGGG	134	20	2.46×10^{-143}	2.74
CCGCGGGG	64	5	2.00×10^{-153}	3.68
CCGGCAG	110	35	7.89×10^{-37}	1.65
CCGGCC	566	174	4.42×10^{-194}	1.70
CCGGCCC	218	61	7.08×10^{-90}	1.84
CCGGCCCC	89	23	4.31×10^{-43}	1.95
CCGGCCCT	72	16	1.56×10^{-44}	2.17
CCGGCCG	123	18	3.19×10^{-135}	2.77
CCGGCCGG	73	6	1.00×10^{-164}	3.60
CCGGCG	203	59	2.03×10^{-78}	1.78
CCGGCGG	100	23	5.22×10^{-58}	2.12
CCGGGAGG	74	23	2.06×10^{-26}	1.69
CCGGGC	692	208	6.25×10^{-247}	1.73
CCGGGCAG	86	16	1.43×10^{-68}	2.43
CCGGGCC	240	62	3.76×10^{-113}	1.95
CCGGGCCC	120	23	5.78×10^{-91}	2.38
CCGGGCCG	77	3	0.00	4.68
CCGGGCG	136	35	2.39×10^{-65}	1.96
CCGGGCGG	76	7	6.21×10^{-150}	3.44
CCGGGCT	165	53	2.08×10^{-53}	1.64
CCGGGCTG	94	19	2.38×10^{-66}	2.31
CCGGGG	877	207	0.00	2.08
CCGGGGA	152	49	5.21×10^{-49}	1.63
CCGGGGC	316	54	2.04×10^{-278}	2.55
CCGGGGCA	65	14	2.64×10^{-42}	2.22
CCGGGGCC	131	19	1.34×10^{-145}	2.79
CCGGGGCG	70	5	8.88×10^{-186}	3.81
CCGGGGCT	86	18	8.17×10^{-58}	2.26
CCGGGGG	256	49	3.45×10^{-192}	2.39
CCGGGGGC	92	18	3.96×10^{-68}	2.35

Continued on next page...

Table 1 – continued from previous page

Element	Count next to SS	Background count	Chi-square test	LOD
CCGGGGGG	77	15	1.12×10^{-57}	2.36
CCGGGGT	141	39	5.73×10^{-60}	1.85
CCGGGGTG	64	13	2.01×10^{-45}	2.30
CCTCCCCG	87	21	4.99×10^{-47}	2.05
CCTGCCCG	75	21	4.73×10^{-32}	1.84
CCTGGGCG	78	17	1.58×10^{-49}	2.20
CCTGGGGC	232	70	1.58×10^{-83}	1.73
CGCCCCC	181	58	1.12×10^{-58}	1.64
CGCCCCG	111	22	2.75×10^{-80}	2.33
CGCCCG	233	62	1.41×10^{-104}	1.91
CGCCCGC	93	26	1.95×10^{-39}	1.84
CGCCCGG	103	20	6.84×10^{-77}	2.36
CGCCCTT	68	20	7.11×10^{-27}	1.77
CGCCGC	166	52	2.69×10^{-56}	1.67
CGCCGCC	81	17	2.45×10^{-54}	2.25
CGCCGG	167	51	2.49×10^{-59}	1.71
CGCCGGC	65	11	1.33×10^{-59}	2.56
CGCCGGG	89	27	8.06×10^{-33}	1.72
CGCGC	447	140	1.97×10^{-148}	1.67
CGCGCC	164	40	1.37×10^{-85}	2.04
CGCGCCC	71	20	3.99×10^{-30}	1.83
CGCGCG	125	29	4.37×10^{-71}	2.11
CGCGGC	190	54	1.80×10^{-76}	1.81
CGCGGCC	83	21	1.05×10^{-41}	1.98
CGCGGG	280	70	4.95×10^{-139}	2.00
CGCGGGC	96	20	9.07×10^{-65}	2.26
CGCGGGG	107	24	2.19×10^{-64}	2.16
CGCGTC	78	22	7.38×10^{-33}	1.83
CGCTTTT	65	16	1.68×10^{-34}	2.02
CGGCCCC	191	57	1.76×10^{-70}	1.74
CGGCCCCC	79	15	2.43×10^{-61}	2.40
CGGCCCG	105	20	1.50×10^{-80}	2.39
CGGCCG	282	79	1.85×10^{-115}	1.84
CGGCCGC	101	25	3.53×10^{-52}	2.01
CGGCCGG	136	21	5.62×10^{-139}	2.70
CGGCCGGG	75	6	1.40×10^{-174}	3.64
CGGCGG	245	68	3.33×10^{-102}	1.85
CGGCGGG	138	28	5.54×10^{-96}	2.30
CGGCTGGG	70	15	9.03×10^{-46}	2.22
CGGGAGGG	81	21	3.60×10^{-39}	1.95
CGGGCAG	184	59	1.51×10^{-59}	1.64
CGGGCAGG	84	18	1.44×10^{-54}	2.22
				Continued on next page...

Table 1 – continued from previous page

Element	Count next to SS	Background count	Chi-square test	LOD
CGGGCC	524	158	2.10×10^{-186}	1.73
CGGGCCC	205	54	7.90×10^{-94}	1.92
CGGGCCCC	71	15	2.19×10^{-47}	2.24
CGGGCCG	135	19	4.89×10^{-156}	2.83
CGGGCCGG	81	6	6.91×10^{-206}	3.75
CGGGCG	295	84	2.78×10^{-117}	1.81
CGGGCGC	97	23	1.03×10^{-53}	2.08
CGGGCGG	153	25	1.52×10^{-144}	2.61
CGGGCGGG	86	6	5.80×10^{-234}	3.84
CGGGCTGG	83	16	5.66×10^{-63}	2.38
CGGGGAC	106	34	4.99×10^{-35}	1.64
CGGGGC	880	219	0.00	2.01
CGGGGCA	168	43	5.17×10^{-81}	1.97
CGGGGCAG	89	20	1.05×10^{-53}	2.15
CGGGGCC	335	60	4.45×10^{-276}	2.48
CGGGGCCC	106	13	1.05×10^{-146}	3.03
CGGGGCCG	82	12	8.44×10^{-91}	2.77
CGGGGCCT	86	20	2.73×10^{-49}	2.10
CGGGGCG	175	32	5.40×10^{-141}	2.45
CGGGGCGG	107	13	7.78×10^{-150}	3.04
CGGGGCTG	127	34	2.87×10^{-57}	1.90
CGGGGGC	241	69	3.02×10^{-95}	1.80
CGGGGGCC	89	21	8.21×10^{-50}	2.08
CGGGGGG	176	58	3.79×10^{-54}	1.60
CGGGGGGC	78	18	2.09×10^{-45}	2.12
CGGGGGGG	67	20	7.81×10^{-26}	1.74
CGGGGGT	108	34	6.63×10^{-37}	1.67
CGGGGTG	158	51	9.46×10^{-51}	1.63
CGGGGTGG	80	20	4.84×10^{-41}	2.00
CGGGTGGG	85	23	3.13×10^{-38}	1.89
CTCGCG	88	27	7.99×10^{-32}	1.70
CTCGGGG	201	54	5.04×10^{-89}	1.90
CTCGGGGC	69	12	7.78×10^{-61}	2.52
CTGCCCCG	91	27	7.35×10^{-35}	1.75
CTGCCCGC	67	21	1.04×10^{-23}	1.67
CTGCCCGG	70	21	1.10×10^{-26}	1.74
CTGCGGGG	71	16	5.09×10^{-43}	2.15
CTGGCGGG	72	15	4.99×10^{-49}	2.26
CTGGGCCC	220	65	2.26×10^{-82}	1.76
CTGGGCCG	85	23	3.13×10^{-38}	1.89
CTGGGCGG	79	20	9.65×10^{-40}	1.98
CTGGGGAC	132	43	5.83×10^{-42}	1.62

Continued on next page...

Table 1 – continued from previous page

Element	Count next to SS	Background count	Chi-square test	LOD
CTGGGGC	690	206	2.63×10^{-249}	1.74
CTGGGGCA	171	56	2.70×10^{-53}	1.61
CTGGGGCC	236	62	3.28×10^{-108}	1.93
CTGGGGCG	103	15	2.74×10^{-114}	2.78
CTGGGGGC	241	65	1.19×10^{-105}	1.89
CTGGGGGG	183	49	1.11×10^{-81}	1.90
CTGGGGTC	125	38	3.14×10^{-45}	1.72
CTTTATTT	72	23	1.66×10^{-24}	1.65
CTTTGAT	70	15	9.03×10^{-46}	2.22
CTTTTAAA	70	21	1.10×10^{-26}	1.74
CTTTTATT	68	16	1.22×10^{-38}	2.09
CTTTTTCT	78	19	9.64×10^{-42}	2.04
GACGCTG	66	21	9.25×10^{-23}	1.65
GAGGGGAC	83	27	4.41×10^{-27}	1.62
GAGGGGGC	121	37	2.23×10^{-43}	1.71
GAGGGGGG	106	31	2.33×10^{-41}	1.77
GCAGCCCC	134	43	8.67×10^{-44}	1.64
GCAGGGCC	159	50	1.30×10^{-53}	1.67
GCCAGGCG	64	18	2.17×10^{-27}	1.83
GCCAGGGC	172	55	4.52×10^{-56}	1.64
GCCAGGGG	137	39	1.70×10^{-55}	1.81
GCCAGGGT	100	27	7.82×10^{-45}	1.89
GCCCCCCC	105	30	1.12×10^{-42}	1.81
GCCCCCCG	70	23	1.12×10^{-22}	1.61
GCCCCCG	212	66	3.25×10^{-72}	1.68
GCCCCCGC	64	14	9.93×10^{-41}	2.19
GCCCCCGG	77	21	2.42×10^{-34}	1.87
GCCCCCTC	116	37	1.44×10^{-38}	1.65
GCCCCCTG	122	37	2.25×10^{-44}	1.72
GCCCCG	661	201	5.91×10^{-231}	1.72
GCCCCGC	227	54	1.50×10^{-122}	2.07
GCCCCGCC	126	26	1.23×10^{-85}	2.28
GCCCCGG	268	67	3.69×10^{-133}	2.00
GCCCCGGC	65	16	1.68×10^{-34}	2.02
GCCCCGGG	126	23	2.55×10^{-102}	2.45
GCCCCTCC	187	57	1.91×10^{-66}	1.71
GCCCCTGC	167	54	2.32×10^{-53}	1.63
GCCCGC	501	143	6.23×10^{-197}	1.81
GCCCGCC	224	48	2.30×10^{-142}	2.22
GCCCGCCC	121	13	3.92×10^{-197}	3.22
GCCCGCG	99	18	2.95×10^{-81}	2.46
GCCCGG	616	185	2.20×10^{-220}	1.74

Continued on next page...

Table 1 – continued from previous page

Element	Count next to SS	Background count	Chi-square test	LOD
GCCCGGC	200	55	3.96×10^{-85}	1.86
GCCCGGCC	92	21	3.84×10^{-54}	2.13
GCCCGGG	304	64	9.69×10^{-198}	2.25
GCCCGGGC	94	15	1.75×10^{-92}	2.65
GCCCGGGG	144	23	1.86×10^{-140}	2.65
GCCCGTC	76	25	1.98×10^{-24}	1.60
GCCCTCCC	175	57	4.57×10^{-55}	1.62
GCCGC	1226	379	0.00	1.69
GCCGCAC	67	18	7.43×10^{-31}	1.90
GCCGCC	484	143	7.27×10^{-179}	1.76
GCCGCCC	230	52	1.57×10^{-134}	2.15
GCCGCCCC	88	13	4.21×10^{-96}	2.76
GCCGCCG	107	29	1.52×10^{-47}	1.88
GCCGCG	265	59	1.93×10^{-158}	2.17
GCCGCGC	90	19	1.19×10^{-59}	2.24
GCCGCGG	134	20	2.46×10^{-143}	2.74
GCCGCGGG	69	8	3.68×10^{-103}	3.11
GCCGCTC	87	17	1.20×10^{-64}	2.36
GCCGGC	420	119	1.34×10^{-167}	1.82
GCCGGCC	207	40	1.19×10^{-153}	2.37
GCCGGCCC	78	13	1.18×10^{-72}	2.58
GCCGGCG	78	17	1.58×10^{-49}	2.20
GCCGGG	856	227	0.00	1.91
GCCGGGC	281	77	1.48×10^{-119}	1.87
GCCGGGCC	109	19	1.03×10^{-94}	2.52
GCCGGGCG	73	22	1.55×10^{-27}	1.73
GCCGGGCT	67	11	5.83×10^{-64}	2.61
GCCGGGG	350	59	0.00	2.57
GCCGGGGA	69	15	3.48×10^{-44}	2.20
GCCGGGGC	130	17	2.29×10^{-165}	2.93
GCCGGGGG	114	18	2.32×10^{-113}	2.66
GCCTGCCC	152	44	1.33×10^{-59}	1.79
GCCTGGGG	240	65	1.79×10^{-104}	1.88
GCGCCC	390	115	4.87×10^{-145}	1.76
GCGCCCC	142	33	2.77×10^{-80}	2.11
GCGCCCG	92	20	2.56×10^{-58}	2.20
GCGCCG	188	59	2.67×10^{-63}	1.67
GCGCCGC	65	16	1.68×10^{-34}	2.02
GCGCCGG	76	15	6.85×10^{-56}	2.34
GCGCCTC	83	26	5.19×10^{-29}	1.67
GCGCGC	142	43	1.68×10^{-51}	1.72
GCGCGG	218	50	8.86×10^{-125}	2.12

Continued on next page...

Table 1 – continued from previous page

Element	Count next to SS	Background count	Chi-square test	LOD
GCGCGGC	65	10	9.39×10^{-68}	2.70
GCGCGGG	104	20	1.04×10^{-78}	2.38
GCGGCCC	162	45	3.99×10^{-68}	1.85
GCGGCCCC	71	15	2.19×10^{-47}	2.24
GCGGCCG	114	21	1.44×10^{-91}	2.44
GCGGCG	209	53	7.25×10^{-102}	1.98
GCGGCGG	96	21	3.32×10^{-60}	2.19
GCGGCGGG	76	10	9.81×10^{-97}	2.93
GCGGGAGG	75	21	4.73×10^{-32}	1.84
GCGGGC	530	154	1.15×10^{-201}	1.78
GCGGGCC	175	40	4.29×10^{-101}	2.13
GCGGGCG	142	33	2.77×10^{-80}	2.11
GCGGGCGG	75	10	6.96×10^{-94}	2.91
GCGGGCT	106	30	8.89×10^{-44}	1.82
GCGGGG	851	245	0.00	1.80
GCGGGGAG	67	21	1.04×10^{-23}	1.67
GCGGGGC	380	70	1.63×10^{-300}	2.44
GCGGGGCC	131	22	1.84×10^{-119}	2.57
GCGGGGCG	101	11	3.70×10^{-162}	3.20
GCGGGGCT	102	25	1.63×10^{-53}	2.03
GCGGGGG	239	72	3.11×10^{-86}	1.73
GCGGGGGC	79	21	1.03×10^{-36}	1.91
GCGGGGGG	80	24	2.93×10^{-30}	1.74
GCGGGGT	115	35	1.15×10^{-41}	1.72
GCTCGGG	122	35	5.92×10^{-49}	1.80
GCTGCCCC	178	56	9.38×10^{-60}	1.67
GCTGGGCC	212	58	6.34×10^{-91}	1.87
GCTGGGGC	205	54	7.90×10^{-94}	1.92
GCTGGGGG	244	65	3.25×10^{-109}	1.91
GCTTGGGG	94	30	1.52×10^{-31}	1.65
GGCCAGGG	193	54	8.47×10^{-80}	1.84
GGCCCCCG	70	18	1.55×10^{-34}	1.96
GGCCCCCT	98	25	2.81×10^{-48}	1.97
GGCCCCG	263	69	1.22×10^{-120}	1.93
GGCCCCGC	91	16	1.93×10^{-78}	2.51
GGCCCCGG	118	27	1.14×10^{-68}	2.13
GGCCCCTC	111	30	1.74×10^{-49}	1.89
GGCCCGC	114	37	1.00×10^{-36}	1.62
GGCCCGG	240	71	1.76×10^{-89}	1.76
GGCCCGGC	76	15	6.85×10^{-56}	2.34
GGCCCGGG	112	27	3.79×10^{-60}	2.05
GGCCCGT	72	18	4.13×10^{-37}	2.00

Continued on next page...

Table 1 – continued from previous page

Element	Count next to SS	Background count	Chi-square test	LOD
GGCCGC	442	136	9.28×10^{-152}	1.70
GGCCGCA	88	27	7.99×10^{-32}	1.70
GGCCGCCC	72	16	1.56×10^{-44}	2.17
GGCCGCG	106	22	1.00×10^{-71}	2.27
GGCCGCGG	65	9	9.24×10^{-78}	2.85
GGCCGG	679	197	1.82×10^{-258}	1.79
GGCCGGC	180	45	4.48×10^{-90}	2.00
GGCCGGCC	71	16	5.09×10^{-43}	2.15
GGCCGGG	390	87	1.70×10^{-231}	2.16
GGCCGGGA	68	18	4.66×10^{-32}	1.92
GGCCGGGC	141	32	9.82×10^{-83}	2.14
GGCCGGGG	152	26	8.19×10^{-135}	2.55
GGCCGTGG	71	21	1.02×10^{-27}	1.76
GGCGCC	405	134	3.26×10^{-121}	1.60
GGCGCCC	148	39	3.20×10^{-68}	1.92
GGCGCCG	87	20	9.67×10^{-51}	2.12
GGCGCG	190	56	1.05×10^{-71}	1.76
GGCGCGG	96	24	6.74×10^{-49}	2.00
GGCGCTGG	67	21	1.04×10^{-23}	1.67
GGCGGCC	183	45	4.89×10^{-94}	2.02
GGCGGCG	89	20	1.05×10^{-53}	2.15
GGCGGGAG	75	18	3.77×10^{-41}	2.06
GGCGGGC	226	67	4.72×10^{-84}	1.75
GGCGGGCC	67	16	3.12×10^{-37}	2.07
GGCGGGCG	67	15	4.24×10^{-41}	2.16
GGCGGGG	414	104	5.72×10^{-203}	1.99
GGCGGGGC	187	37	2.87×10^{-134}	2.34
GGCGGGGG	139	33	4.99×10^{-76}	2.07
GGCTCGG	116	38	1.07×10^{-36}	1.61
GGCTCGGG	64	13	2.01×10^{-45}	2.30
GGCTGGGC	221	71	6.82×10^{-71}	1.64
GGCTGGGG	299	92	2.67×10^{-103}	1.70
GGGAGGGT	122	32	5.40×10^{-57}	1.93
GGGCAGGG	359	107	4.29×10^{-131}	1.75
GGGCCCC	470	142	8.58×10^{-167}	1.73
GGGCCCCC	100	31	2.86×10^{-35}	1.69
GGGCCCCG	133	21	6.35×10^{-132}	2.66
GGGCCCCT	118	38	1.63×10^{-38}	1.63
GGGCCCG	215	59	1.05×10^{-91}	1.87
GGGCCCGG	119	18	2.89×10^{-125}	2.72
GGGCCCTG	192	62	3.10×10^{-61}	1.63
GGGCCG	632	191	1.88×10^{-223}	1.73

Continued on next page...

Table 1 – continued from previous page

Element	Count next to SS	Background count	Chi-square test	LOD
GGGCCGC	139	42	1.20×10^{-50}	1.73
GGGCCGG	353	79	1.11×10^{-208}	2.16
GGGCCGGC	76	14	1.14×10^{-61}	2.44
GGGCCGGG	228	34	1.02×10^{-242}	2.75
GGGCCTGG	273	85	1.98×10^{-92}	1.68
GGGCGCC	154	48	7.65×10^{-53}	1.68
GGGCGCG	76	18	1.52×10^{-42}	2.08
GGGCGCTG	65	18	1.60×10^{-28}	1.85
GGGCGGC	189	48	4.47×10^{-92}	1.98
GGGCGGCC	85	18	3.53×10^{-56}	2.24
GGGCGGG	437	114	4.84×10^{-201}	1.94
GGGCGGGA	74	12	1.22×10^{-71}	2.62
GGGCGGGC	111	20	4.81×10^{-92}	2.47
GGGCGGGG	258	63	2.78×10^{-133}	2.03
GGGCTCG	110	35	7.89×10^{-37}	1.65
GGGCTCGG	66	14	6.55×10^{-44}	2.24
GGGCTGGG	411	124	1.73×10^{-146}	1.73
GGGGCACC	67	20	7.81×10^{-26}	1.74
GGGGCAGG	272	69	6.64×10^{-132}	1.98
GGGGCC	1666	506	0.00	1.72
GGGGCCC	601	151	1.29×10^{-293}	1.99
GGGGCCCA	136	42	1.13×10^{-47}	1.70
GGGGCCCC	176	37	1.41×10^{-115}	2.25
GGGGCCCG	116	17	2.13×10^{-127}	2.77
GGGGCCCT	156	39	2.56×10^{-78}	2.00
GGGGCCG	318	71	6.89×10^{-189}	2.16
GGGGCCGC	73	15	1.06×10^{-50}	2.28
GGGGCCGG	177	29	2.80×10^{-166}	2.61
GGGGCCT	435	140	3.28×10^{-137}	1.64
GGGGCCTC	93	29	1.42×10^{-32}	1.68
GGGGCCTG	238	69	5.09×10^{-92}	1.79
GGGGCG	680	202	5.45×10^{-248}	1.75
GGGGCGC	159	40	5.61×10^{-79}	1.99
GGGGCGCC	68	11	3.37×10^{-66}	2.63
GGGGCGG	374	99	3.77×10^{-168}	1.92
GGGGCGGC	88	14	4.66×10^{-87}	2.65
GGGGCGGG	244	57	1.93×10^{-135}	2.10
GGGGCTGC	185	53	1.79×10^{-73}	1.80
GGGGCTGG	356	111	1.27×10^{-119}	1.68
GGGGGCAG	183	60	8.80×10^{-57}	1.61
GGGGGCC	378	107	2.72×10^{-151}	1.82
GGGGGCCC	126	27	6.24×10^{-81}	2.22

Continued on next page...

Table 1 – continued from previous page

Element	Count next to SS	Background count	Chi-square test	LOD
GGGGGCCG	103	18	2.74×10^{-89}	2.52
GGGGGCG	233	59	1.30×10^{-113}	1.98
GGGGGCGG	154	30	1.78×10^{-113}	2.36
GGGGGGAG	145	42	7.04×10^{-57}	1.79
GGGGGGC	327	107	2.21×10^{-100}	1.61
GGGGGGCA	110	29	3.93×10^{-51}	1.92
GGGGGGCC	81	20	2.31×10^{-42}	2.02
GGGGGGCG	83	18	5.56×10^{-53}	2.21
GGGGGGGC	99	27	1.16×10^{-43}	1.87
GGGGTGGG	450	127	1.12×10^{-180}	1.83
GGGTGGGA	207	68	9.40×10^{-64}	1.61
GGGTGGGC	175	56	6.11×10^{-57}	1.64
GGGTGGGG	460	136	6.85×10^{-170}	1.76
GGTGGGGA	216	68	4.98×10^{-72}	1.67
GGTGGGGG	367	100	4.64×10^{-157}	1.88
GGTGGGGT	153	48	6.96×10^{-52}	1.67
GTGCCGC	64	18	2.17×10^{-27}	1.83
GTGGGCCC	102	32	3.59×10^{-35}	1.67
GTGGGCGG	69	21	1.13×10^{-25}	1.72
GTGGGGAC	96	30	1.94×10^{-33}	1.68
GTGGGGCC	163	42	8.54×10^{-78}	1.96
GTGGGGCG	68	16	1.22×10^{-38}	2.09
GTGGGGGC	155	49	8.43×10^{-52}	1.66
GTGGGGGG	200	64	8.18×10^{-65}	1.64
TAATTTC	70	22	1.40×10^{-24}	1.67
TATTCAT	65	17	2.53×10^{-31}	1.93
TATTTTAT	98	24	1.50×10^{-51}	2.03
TATTTTCT	85	27	6.24×10^{-29}	1.65
TATTTTTT	129	41	5.58×10^{-43}	1.65
TCCGCCC	85	23	3.13×10^{-38}	1.89
TCCGGGG	109	31	1.37×10^{-44}	1.81
TGCGCCC	64	17	4.22×10^{-30}	1.91
TGCGGGG	134	42	9.69×10^{-46}	1.67
TGGCCCCG	64	17	4.22×10^{-30}	1.91
TGGGCCCC	124	39	3.44×10^{-42}	1.67
TGGGCCGG	81	21	3.60×10^{-39}	1.95
TGGGCGGG	99	28	4.76×10^{-41}	1.82
TGGGGCAC	83	23	6.51×10^{-36}	1.85
TGGGGCC	541	153	5.36×10^{-216}	1.82
TGGGGCCA	126	40	4.12×10^{-42}	1.66
TGGGGCCC	195	43	7.25×10^{-119}	2.18
TGGGGCCG	75	17	6.05×10^{-45}	2.14

Continued on next page...

Table 1 – continued from previous page

Element	Count next to SS	Background count	Chi-square test	LOD
TGGGGCCT	148	41	1.09×10^{-62}	1.85
TGGGGCG	193	51	5.58×10^{-88}	1.92
TGGGGCGG	91	17	5.00×10^{-72}	2.42
TGGGGGCC	152	49	5.21×10^{-49}	1.63
TGGGGGCG	72	18	4.13×10^{-37}	2.00
TGGGGGGC	154	51	3.71×10^{-47}	1.59
TTTATTC	101	33	2.50×10^{-32}	1.61
TTTCTTTA	65	20	8.10×10^{-24}	1.70
TTTCTTTT	175	54	6.42×10^{-61}	1.70
TTTTAATT	81	26	3.99×10^{-27}	1.64
TTTTCTTA	64	20	7.67×10^{-23}	1.68
TTTTTAAA	141	42	1.10×10^{-52}	1.75
TTTTTATT	115	35	1.15×10^{-41}	1.72
TTTTTCCT	70	20	5.09×10^{-29}	1.81
TTTTTCTT	142	40	1.63×10^{-58}	1.83
TTTTTTA	258	82	3.81×10^{-84}	1.65
TTTTTTAA	127	34	2.87×10^{-57}	1.90
TTTTTTCT	114	32	1.29×10^{-47}	1.83
TTTTTTTA	135	37	2.13×10^{-58}	1.87
**5′SS ISS**				
AAAGGT	27	81	1.97×10^{-09}	-1.58
AAAGGTA	31	113	1.22×10^{-14}	-1.87
AAGGTA	101	383	4.43×10^{-47}	-1.92
AAGGTAA	16	121	1.35×10^{-21}	-2.92
AAGGTAG	27	91	1.96×10^{-11}	-1.75
AAGGTAT	27	92	1.23×10^{-11}	-1.77
AAGGTGA	43	149	3.82×10^{-18}	-1.79
AAGGTGAG	5	66	5.97×10^{-14}	-3.72
AAGTAAG	24	115	2.14×10^{-17}	-2.26
AATCCCAG	18	87	1.39×10^{-13}	-2.27
ACACACA	135	505	6.38×10^{-61}	-1.90
ACACACAC	45	342	4.78×10^{-58}	-2.93
ACAGGTA	25	112	2.02×10^{-16}	-2.16
AGAAGAAG	21	64	7.66×10^{-08}	-1.61
AGACAGAG	30	98	6.46×10^{-12}	-1.71
AGACCAGC	13	75	8.11×10^{-13}	-2.53
AGAGACAG	29	96	8.01×10^{-12}	-1.73
AGAGAGA	166	598	7.39×10^{-70}	-1.85
AGAGAGAC	20	64	3.80×10^{-08}	-1.68
AGAGAGAG	64	332	5.61×10^{-49}	-2.38
AGAGGTA	32	96	6.49×10^{-11}	-1.58
AGGCAAG	47	147	1.61×10^{-16}	-1.65

Continued on next page...

Table 1 – continued from previous page

Element	Count next to SS	Background count	Chi-square test	LOD
AGGTA	27	139	2.10×10^{-21}	-2.36
AGGTAA	52	446	1.08×10^{-77}	-3.10
AGGTAAA	14	132	9.53×10^{-25}	-3.24
AGGTAAC	16	72	4.12×10^{-11}	-2.17
AGGTAAG	5	131	3.46×10^{-28}	-4.71
AGGTAAT	13	105	2.75×10^{-19}	-3.01
AGGTAC	69	233	6.29×10^{-27}	-1.76
AGGTACA	16	81	5.11×10^{-13}	-2.34
AGGTACT	19	75	1.00×10^{-10}	-1.98
AGGTAG	101	362	7.82×10^{-43}	-1.84
AGGTAGA	34	116	2.66×10^{-14}	-1.77
AGGTAGG	20	114	1.32×10^{-18}	-2.51
AGGTAGT	20	67	9.35×10^{-09}	-1.74
AGGTAT	81	327	3.75×10^{-42}	-2.01
AGGTATA	13	84	9.42×10^{-15}	-2.69
AGGTATG	10	92	1.24×10^{-17}	-3.20
AGGTATT	32	114	1.59×10^{-14}	-1.83
AGGTCAG	43	187	6.23×10^{-26}	-2.12
AGGTCAGG	24	73	9.75×10^{-09}	-1.60
AGGTCT	31	95	5.15×10^{-11}	-1.62
AGGTGA	160	569	6.48×10^{-66}	-1.83
AGGTGAA	36	134	2.54×10^{-17}	-1.90
AGGTGAG	26	231	1.82×10^{-41}	-3.15
AGGTGAGG	12	79	4.76×10^{-14}	-2.72
AGGTGC	27	89	4.96×10^{-11}	-1.72
AGGTGGG	16	75	9.57×10^{-12}	-2.23
AGGTTAG	12	76	2.11×10^{-13}	-2.66
AGTAAGT	30	126	1.20×10^{-17}	-2.07
AGTACAG	38	118	1.77×10^{-13}	-1.63
AGTAGCTG	18	64	8.92×10^{-09}	-1.83
AGTATAG	26	78	3.91×10^{-09}	-1.58
AGTGCAGT	13	69	1.57×10^{-11}	-2.41
ATAGGTA	22	66	6.09×10^{-08}	-1.58
ATCCCAGC	31	97	2.06×10^{-11}	-1.65
ATGTAAG	31	107	2.02×10^{-13}	-1.79
CAACGA	21	67	1.91×10^{-08}	-1.67
CAAGGTA	18	87	1.39×10^{-13}	-2.27
CACACAC	127	472	8.51×10^{-57}	-1.89
CACACACA	54	358	4.27×10^{-58}	-2.73
CACCACCA	25	76	4.91×10^{-09}	-1.60
CAGAAGAG	25	76	4.91×10^{-09}	-1.60
CAGAGAAG	26	82	6.24×10^{-10}	-1.66
				Continued on next page...

Table 1 – continued from previous page

Element	Count next to SS	Background count	Chi-square test	LOD
CAGAGAGA	35	117	3.43×10^{-14}	-1.74
CAGATAG	29	94	2.02×10^{-11}	-1.70
CAGGAGAA	22	66	6.09×10^{-08}	-1.58
CAGGAGTT	12	75	3.47×10^{-13}	-2.64
CAGGTA	82	388	1.98×10^{-54}	-2.24
CAGGTAA	10	97	1.01×10^{-18}	-3.28
CAGGTAC	18	72	1.96×10^{-10}	-2.00
CAGGTAG	23	110	1.08×10^{-16}	-2.26
CAGGTAT	26	109	1.86×10^{-15}	-2.07
CAGGTGA	39	185	7.01×10^{-27}	-2.25
CAGGTGAG	5	85	4.05×10^{-18}	-4.09
CAGTAAG	27	98	7.38×10^{-13}	-1.86
CCAGGTA	24	106	1.66×10^{-15}	-2.14
CCCAGGTT	19	69	1.75×10^{-09}	-1.86
CCTGTAA	38	148	1.54×10^{-19}	-1.96
CCTGTAAT	5	70	7.90×10^{-15}	-3.81
CGGTGAG	19	66	7.23×10^{-09}	-1.80
CTATCTA	23	76	1.20×10^{-09}	-1.72
CTGAGGCA	23	86	1.09×10^{-11}	-1.90
CTGTAATC	9	72	1.13×10^{-13}	-3.00
GAAGGA	47	158	1.04×10^{-18}	-1.75
GAAGGTA	24	86	2.30×10^{-11}	-1.84
GACCAGCC	20	69	3.66×10^{-09}	-1.79
GAGAAAGA	31	96	3.26×10^{-11}	-1.63
GAGAAGCA	16	69	1.76×10^{-10}	-2.11
GAGACAGA	24	114	3.47×10^{-17}	-2.25
GAGAGACA	19	74	1.62×10^{-10}	-1.96
GAGAGAGA	64	322	7.03×10^{-47}	-2.33
GAGATAG	31	93	1.28×10^{-10}	-1.58
GAGGAAGA	27	81	1.97×10^{-09}	-1.58
GAGGTA	84	292	4.33×10^{-34}	-1.80
GAGGTAA	13	87	2.13×10^{-15}	-2.74
GAGGTAG	24	104	4.34×10^{-15}	-2.12
GAGGTCAG	21	68	1.20×10^{-08}	-1.70
GAGGTGA	52	166	8.88×10^{-19}	-1.67
GAGTGCAG	20	82	7.55×10^{-12}	-2.04
GATCGA	27	84	4.99×10^{-10}	-1.64
GCAGGTA	24	92	1.34×10^{-12}	-1.94
GCAGGTG	20	67	9.35×10^{-09}	-1.74
GCAGGTGA	15	63	1.47×10^{-09}	-2.07
GGACTAC	20	64	3.80×10^{-08}	-1.68
GGAGGTA	23	87	6.81×10^{-12}	-1.92

Continued on next page...

Table 1 – continued from previous page

Element	Count next to SS	Background count	Chi-square test	LOD
GGCAAGT	36	108	4.26×10^{-12}	-1.58
GGCAGGT	17	67	1.01×10^{-09}	-1.98
GGGTAAG	22	76	5.85×10^{-10}	-1.79
GGTAA	43	147	9.65×10^{-18}	-1.77
GGTAAAG	30	106	1.56×10^{-13}	-1.82
GGTAAAT	34	106	2.68×10^{-12}	-1.64
GGTAAG	43	314	8.33×10^{-53}	-2.87
GGTAAGA	18	94	4.54×10^{-15}	-2.38
GGTAAGG	16	81	5.11×10^{-13}	-2.34
GGTAAGT	6	92	3.07×10^{-19}	-3.94
GGTAGAT	19	67	4.51×10^{-09}	-1.82
GGTAGGA	24	91	2.16×10^{-12}	-1.92
GGTAGGT	27	86	1.99×10^{-10}	-1.67
GGTATGT	20	86	1.10×10^{-12}	-2.10
GGTCAGT	34	107	1.70×10^{-12}	-1.65
GGTGAGC	44	134	7.54×10^{-15}	-1.61
GGTGAGT	14	161	4.87×10^{-31}	-3.52
GGTGAGTG	4	67	1.40×10^{-14}	-4.07
GGTTAAG	21	95	3.14×10^{-14}	-2.18
GGTTCAA	40	131	1.85×10^{-15}	-1.71
GTAAG	36	170	8.88×10^{-25}	-2.24
GTAAGCA	21	72	1.85×10^{-09}	-1.78
GTAAGGA	23	94	2.42×10^{-13}	-2.03
GTAAGT	81	369	8.05×10^{-51}	-2.19
GTAAGTA	12	98	3.71×10^{-18}	-3.03
GTAAGTC	16	65	1.22×10^{-09}	-2.02
GTAAGTG	25	116	2.93×10^{-17}	-2.21
GTAAGTT	23	101	8.40×10^{-15}	-2.13
GTAATCC	20	87	6.81×10^{-13}	-2.12
GTAGAAC	17	69	3.85×10^{-10}	-2.02
GTAGGTA	14	65	2.52×10^{-10}	-2.22
GTGAAG	38	116	4.41×10^{-13}	-1.61
GTGAGT	14	90	1.14×10^{-15}	-2.68
GTGAGTA	23	91	1.01×10^{-12}	-1.98
GTGCAA	22	73	2.38×10^{-09}	-1.73
GTGGCTCA	18	87	1.39×10^{-13}	-2.27
GTTAGTA	21	68	1.20×10^{-08}	-1.70
TAAGCAC	28	97	2.45×10^{-12}	-1.79
TAAGCG	26	78	3.91×10^{-09}	-1.58
TAAGGTA	15	72	1.85×10^{-11}	-2.26
TAAGTGC	28	89	1.01×10^{-10}	-1.67
TAATCCC	30	143	3.39×10^{-21}	-2.25

Continued on next page...

Table 1 – continued from previous page

Element	Count next to SS	Background count	Chi-square test	LOD
TAATCCCA	6	92	3.07×10^{-19}	-3.94
TACAGATG	21	67	1.91×10^{-08}	-1.67
TAGATAG	27	82	1.25×10^{-09}	-1.60
TAGATGA	33	113	5.23×10^{-14}	-1.78
TAGCTGGG	19	66	7.23×10^{-09}	-1.80
TAGGACT	21	69	7.53×10^{-09}	-1.72
TAGGTA	65	261	7.08×10^{-34}	-2.01
TAGGTAA	9	77	9.23×10^{-15}	-3.10
TAGGTAG	17	67	1.01×10^{-09}	-1.98
TAGGTAT	19	76	6.22×10^{-11}	-2.00
TAGGTCA	19	64	1.85×10^{-08}	-1.75
TAGGTGA	24	80	3.82×10^{-10}	-1.74
TAGGTTG	21	64	7.66×10^{-08}	-1.61
TAGTAAG	23	75	1.92×10^{-09}	-1.71
TAGTTAG	24	72	1.54×10^{-08}	-1.58
TAGTTGC	20	67	9.35×10^{-09}	-1.74
TCAGGTA	23	101	8.40×10^{-15}	-2.13
TCTCTCTC	92	308	8.15×10^{-35}	-1.74
TCTGTAAA	28	90	6.34×10^{-11}	-1.68
TGAGGTA	27	102	1.12×10^{-13}	-1.92
TGGAGTGC	19	66	7.23×10^{-09}	-1.80
TGGTAAG	12	104	1.85×10^{-19}	-3.12
TGTAAGT	28	139	4.72×10^{-21}	-2.31
TGTAATCC	5	68	2.17×10^{-14}	-3.77
TTAGGTA	22	82	3.45×10^{-11}	-1.90
TTTGAGAC	19	91	4.43×10^{-14}	-2.26
**3′SS ISE**				
AAAACTAA	134	41	8.50×10^{-48}	1.71
AAAATGTT	69	18	2.76×10^{-33}	1.94
AAACTAAC	72	11	1.52×10^{-75}	2.71
AAACTAAT	132	26	5.51×10^{-96}	2.34
AAACTGAC	67	21	1.04×10^{-23}	1.67
AAATGACC	64	20	7.67×10^{-23}	1.68
AAATTAAT	169	56	1.61×10^{-51}	1.59
AACACTAA	70	15	9.03×10^{-46}	2.22
AACTAAAA	88	26	5.12×10^{-34}	1.76
AACTAAC	189	36	1.96×10^{-143}	2.39
AACTAACA	69	14	6.50×10^{-49}	2.30
AACTAACT	69	14	6.50×10^{-49}	2.30
AACTAAT	269	78	1.00×10^{-103}	1.79
AACTAATG	76	15	6.85×10^{-56}	2.34
AACTAATT	92	24	8.31×10^{-44}	1.94

Continued on next page...

Table 1 – continued from previous page

Element	Count next to SS	Background count	Chi-square test	LOD
AACTGAC	195	54	4.67×10^{-82}	1.85
AACTTTTT	75	24	2.22×10^{-25}	1.64
AATAACAT	98	30	2.16×10^{-35}	1.71
AATACTAA	88	19	1.94×10^{-56}	2.21
AATACTGA	80	25	3.82×10^{-28}	1.68
AATCTAAT	92	30	1.05×10^{-29}	1.62
AATGCTAA	89	24	3.54×10^{-40}	1.89
AATGTAAC	66	18	1.12×10^{-29}	1.87
AATTAAC	236	69	6.71×10^{-90}	1.77
AATTAACA	79	24	3.01×10^{-29}	1.72
AATTAACT	84	26	5.58×10^{-30}	1.69
AATTCTAA	100	32	2.76×10^{-33}	1.64
ACAAAAT	69	21	1.13×10^{-25}	1.72
ACACTAA	178	50	3.07×10^{-73}	1.83
ACACTAAT	64	18	2.17×10^{-27}	1.83
ACACTCAC	72	18	4.13×10^{-37}	2.00
ACACTGAC	72	16	1.56×10^{-44}	2.17
ACATTGT	69	22	1.24×10^{-23}	1.65
ACCCTCAC	87	19	7.24×10^{-55}	2.20
ACCCTCTC	70	20	5.09×10^{-29}	1.81
ACCCTGAC	97	27	2.30×10^{-41}	1.85
ACCTAAC	117	36	1.56×10^{-41}	1.70
ACCTCACC	80	16	1.28×10^{-57}	2.32
ACCTCACT	83	22	1.14×10^{-38}	1.92
ACCTGAC	213	64	2.01×10^{-77}	1.73
ACCTGACC	78	22	7.38×10^{-33}	1.83
ACCTGACT	67	19	3.34×10^{-28}	1.82
ACGCTGA	73	19	3.02×10^{-35}	1.94
ACTAAATT	68	21	1.11×10^{-24}	1.70
ACTAAC	571	134	0.00	2.09
ACTAACA	187	48	1.55×10^{-89}	1.96
ACTAACAT	74	14	7.19×10^{-58}	2.40
ACTAACC	182	35	2.74×10^{-136}	2.38
ACTAACCT	68	13	1.54×10^{-52}	2.39
ACTAACG	64	7	6.03×10^{-103}	3.19
ACTAACT	178	43	3.55×10^{-94}	2.05
ACTAACTT	78	14	1.37×10^{-65}	2.48
ACTAAT	835	243	0.00	1.78
ACTAATAT	76	22	1.14×10^{-30}	1.79
ACTAATC	139	35	3.55×10^{-69}	1.99
ACTAATG	241	58	1.37×10^{-127}	2.05
ACTAATGA	64	14	9.93×10^{-41}	2.19
				Continued on next page...

Table 1 – continued from previous page

Element	Count next to SS	Background count	Chi-square test	LOD
ACTAATGT	89	17	2.76×10^{-68}	2.39
ACTAATT	276	81	4.20×10^{-104}	1.77
ACTAATTA	64	19	5.50×10^{-25}	1.75
ACTAATTT	129	36	3.46×10^{-54}	1.84
ACTCACC	213	54	7.99×10^{-104}	1.98
ACTCACCC	70	16	1.56×10^{-41}	2.13
ACTCACCT	83	22	1.14×10^{-38}	1.92
ACTCACTC	66	13	6.48×10^{-49}	2.34
ACTCTAAT	64	15	1.09×10^{-36}	2.09
ACTCTGAC	72	21	9.05×10^{-29}	1.78
ACTGAC	879	244	0.00	1.85
ACTGACAT	74	20	1.43×10^{-33}	1.89
ACTGACC	308	66	5.49×10^{-195}	2.22
ACTGACCA	81	24	2.73×10^{-31}	1.75
ACTGACCC	114	19	2.61×10^{-105}	2.58
ACTGACCT	98	25	2.81×10^{-48}	1.97
ACTGACG	69	10	1.10×10^{-77}	2.79
ACTGACT	298	93	2.78×10^{-100}	1.68
ACTGACTC	64	19	5.50×10^{-25}	1.75
ACTGACTG	94	30	1.52×10^{-31}	1.65
ACTGACTT	100	27	7.82×10^{-45}	1.89
ACTGATC	147	42	4.88×10^{-59}	1.81
ACTGATTT	128	37	1.33×10^{-50}	1.79
ACTTACAT	65	20	8.10×10^{-24}	1.70
AGACTGAC	74	18	8.85×10^{-40}	2.04
AGCCCCTC	95	30	1.75×10^{-32}	1.66
AGCCCTGA	139	40	3.15×10^{-55}	1.80
AGCCTCAC	83	24	2.10×10^{-33}	1.79
AGCCTGAC	96	26	6.88×10^{-43}	1.88
AGCTAAC	129	40	5.62×10^{-45}	1.69
AGCTCACC	66	20	8.15×10^{-25}	1.72
AGCTCACT	65	20	8.10×10^{-24}	1.70
AGCTGAC	257	79	3.21×10^{-89}	1.70
AGCTGACC	95	19	4.42×10^{-68}	2.32
AGCTGACT	83	19	8.33×10^{-49}	2.13
AGGCCTCA	81	26	3.99×10^{-27}	1.64
AGGCTCAC	65	19	4.91×10^{-26}	1.77
AGGCTGAC	95	21	1.17×10^{-58}	2.18
AGTAACC	140	44	1.80×10^{-47}	1.67
AGTAACTT	86	26	5.77×10^{-32}	1.73
ATAACATT	104	34	3.35×10^{-33}	1.61
ATAACTAA	84	20	1.87×10^{-46}	2.07

Continued on next page...

Table 1 – continued from previous page

Element	Count next to SS	Background count	Chi-square test	LOD
ATAACTGA	71	22	1.51×10^{-25}	1.69
ATACTAA	245	69	1.23×10^{-99}	1.83
ATACTAAT	81	14	1.05×10^{-71}	2.53
ATACTGAT	71	21	1.02×10^{-27}	1.76
ATACTTAA	75	19	8.90×10^{-38}	1.98
ATACTTT	79	22	5.56×10^{-34}	1.84
ATATTAAC	86	20	2.73×10^{-49}	2.10
ATATTAT	72	23	1.66×10^{-24}	1.65
ATATTTTT	91	29	1.13×10^{-30}	1.65
ATCTAAC	138	35	6.89×10^{-68}	1.98
ATCTAATG	75	19	8.90×10^{-38}	1.98
ATGACTAA	67	21	1.04×10^{-23}	1.67
ATGACTGA	76	23	2.16×10^{-28}	1.72
ATGCTAA	254	74	3.18×10^{-97}	1.78
ATGCTAAC	76	13	2.30×10^{-68}	2.55
ATGCTAAT	91	22	5.49×10^{-49}	2.05
ATGCTCAC	72	12	3.29×10^{-67}	2.58
ATGCTCAT	68	18	4.66×10^{-32}	1.92
ATGCTGAC	95	19	4.42×10^{-68}	2.32
ATGCTGAT	84	23	4.61×10^{-37}	1.87
ATGTTAAC	67	17	7.61×10^{-34}	1.98
ATTAACAT	87	28	7.16×10^{-29}	1.64
ATTAACC	159	48	9.02×10^{-58}	1.73
ATTAACT	263	85	4.68×10^{-83}	1.63
ATTAACTG	74	15	2.11×10^{-52}	2.30
ATTAACTT	102	27	3.17×10^{-47}	1.92
ATTAATCT	76	24	2.55×10^{-26}	1.66
ATTAATGA	84	27	5.34×10^{-28}	1.64
ATTAATTT	198	63	7.09×10^{-65}	1.65
ATTACTAA	76	24	2.55×10^{-26}	1.66
ATTATTG	74	24	1.86×10^{-24}	1.62
ATTCTAAC	74	19	1.68×10^{-36}	1.96
ATTCTAAT	117	34	5.60×10^{-46}	1.78
ATTCTCAC	70	16	1.56×10^{-41}	2.13
ATTCTGAC	72	19	5.13×10^{-34}	1.92
ATTGCTAA	69	22	1.24×10^{-23}	1.65
ATTGTAT	64	20	7.67×10^{-23}	1.68
ATTTTATT	103	32	3.91×10^{-36}	1.69
CACCCCTC	92	27	6.64×10^{-36}	1.77
CACCCTGA	99	27	1.16×10^{-43}	1.87
CACCTCAC	85	21	2.51×10^{-44}	2.02
CACCTGAC	83	20	4.55×10^{-45}	2.05

Continued on next page...

Table 1 – continued from previous page

Element	Count next to SS	Background count	Chi-square test	LOD
CACTAAC	187	30	1.06×10^{-180}	2.64
CACTAACA	66	10	3.59×10^{-70}	2.72
CACTAAT	202	53	4.24×10^{-93}	1.93
CACTAATG	67	10	1.24×10^{-72}	2.74
CACTCAC	284	81	1.17×10^{-112}	1.81
CACTCACC	98	18	2.61×10^{-79}	2.44
CACTCACT	103	27	1.91×10^{-48}	1.93
CACTGAC	364	77	1.23×10^{-234}	2.24
CACTGACA	92	19	5.91×10^{-63}	2.28
CACTGACC	169	28	1.96×10^{-156}	2.59
CACTGACT	119	26	2.53×10^{-74}	2.19
CACTGAT	249	79	1.51×10^{-81}	1.66
CACTGATG	85	26	5.79×10^{-31}	1.71
CACTGATT	75	24	2.22×10^{-25}	1.64
CAGCTCAC	84	23	4.61×10^{-37}	1.87
CAGCTGAC	101	21	3.02×10^{-68}	2.27
CAGTGACC	92	27	6.64×10^{-36}	1.77
CATACTAA	65	17	2.53×10^{-31}	1.93
CATCTCAC	65	18	1.60×10^{-28}	1.85
CATCTCAT	82	25	4.18×10^{-30}	1.71
CATCTGAC	69	17	1.82×10^{-36}	2.02
CATGCTGA	68	20	7.11×10^{-27}	1.77
CATTAAC	186	57	1.87×10^{-65}	1.71
CATTAACA	67	20	7.81×10^{-26}	1.74
CATTAACT	65	18	1.60×10^{-28}	1.85
CATTAATG	78	21	1.62×10^{-35}	1.89
CCACCTGA	72	18	4.13×10^{-37}	2.00
CCACTCAC	104	18	2.34×10^{-91}	2.53
CCACTGAC	125	15	1.91×10^{-177}	3.06
CCACTGAT	74	20	1.43×10^{-33}	1.89
CCAGCTGA	92	30	1.05×10^{-29}	1.62
CCCAACCT	64	19	5.50×10^{-25}	1.75
CCCACTCA	106	21	8.37×10^{-77}	2.34
CCCACTGA	112	28	9.50×10^{-57}	2.00
CCCCACTC	109	36	4.67×10^{-34}	1.60
CCCCACTT	70	23	1.12×10^{-22}	1.61
CCCCCCTC	107	30	6.85×10^{-45}	1.83
CCCCCTCA	99	26	1.72×10^{-46}	1.93
CCCCCTGA	119	22	5.18×10^{-95}	2.44
CCCCTCAC	192	33	1.27×10^{-168}	2.54
CCCCTGAC	175	32	5.40×10^{-141}	2.45
CCCTAAC	218	49	8.82×10^{-129}	2.15

Continued on next page...

Table 1 – continued from previous page

Element	Count next to SS	Background count	Chi-square test	LOD
CCCTAACC	88	15	3.02×10^{-79}	2.55
CCCTCAC	442	100	2.36×10^{-256}	2.14
CCCTCACA	72	20	2.98×10^{-31}	1.85
CCCTCACC	193	41	1.44×10^{-124}	2.23
CCCTCACT	141	22	5.27×10^{-142}	2.68
CCCTCTCA	90	26	3.90×10^{-36}	1.79
CCCTCTGA	123	33	2.54×10^{-55}	1.90
CCCTGAC	516	116	6.36×10^{-302}	2.15
CCCTGACA	95	23	6.03×10^{-51}	2.05
CCCTGACC	241	45	1.14×10^{-187}	2.42
CCCTGACT	154	32	3.67×10^{-103}	2.27
CCCTGAT	236	74	4.10×10^{-79}	1.67
CCCTGATC	72	14	3.40×10^{-54}	2.36
CCCTGATG	89	22	2.73×10^{-46}	2.02
CCGCTCA	96	22	4.48×10^{-56}	2.13
CCGCTGA	134	31	2.09×10^{-76}	2.11
CCGCTGAC	82	9	8.69×10^{-131}	3.19
CCGTGAC	92	24	8.31×10^{-44}	1.94
CCTAAC	612	171	2.46×10^{-249}	1.84
CCTAACA	164	46	8.50×10^{-68}	1.83
CCTAACC	198	50	2.82×10^{-97}	1.99
CCTAACCC	77	13	1.71×10^{-70}	2.57
CCTAACG	64	11	1.76×10^{-57}	2.54
CCTAACT	224	64	5.48×10^{-89}	1.81
CCTAACTG	71	22	1.51×10^{-25}	1.69
CCTAACTT	64	15	1.09×10^{-36}	2.09
CCTAATC	104	33	4.32×10^{-35}	1.66
CCTAATG	191	56	9.39×10^{-73}	1.77
CCTCAC	1209	383	0.00	1.66
CCTCACC	477	130	1.91×10^{-203}	1.88
CCTCACCA	95	28	9.62×10^{-37}	1.76
CCTCACCC	190	49	3.10×10^{-90}	1.96
CCTCACCT	160	36	6.89×10^{-95}	2.15
CCTCACG	113	27	1.58×10^{-61}	2.07
CCTCACT	412	112	8.85×10^{-177}	1.88
CCTCACTC	123	23	1.48×10^{-96}	2.42
CCTCACTG	164	47	2.64×10^{-65}	1.80
CCTCACTT	93	27	5.78×10^{-37}	1.78
CCTCCTAA	68	16	1.22×10^{-38}	2.09
CCTCTAA	227	61	3.00×10^{-100}	1.90
CCTCTAAC	74	8	1.98×10^{-120}	3.21
CCTCTAAT	64	13	2.01×10^{-45}	2.30

Continued on next page...

Table 1 – continued from previous page

Element	Count next to SS	Background count	Chi-square test	LOD
CCTCTCAC	132	22	1.26×10^{-121}	2.58
CCTCTCAT	78	19	9.64×10^{-42}	2.04
CCTCTGAC	184	33	2.78×10^{-152}	2.48
CCTCTGAT	74	23	2.06×10^{-26}	1.69
CCTGAC	1284	397	0.00	1.69
CCTGACAC	75	22	1.32×10^{-29}	1.77
CCTGACC	530	152	1.89×10^{-206}	1.80
CCTGACCA	102	30	1.81×10^{-39}	1.77
CCTGACCC	212	47	5.41×10^{-128}	2.17
CCTGACG	129	28	3.22×10^{-81}	2.20
CCTGACT	407	119	1.31×10^{-153}	1.77
CCTGACTC	150	34	4.59×10^{-88}	2.14
CCTGACTG	121	29	1.95×10^{-65}	2.06
CCTGCTCA	138	35	6.89×10^{-68}	1.98
CCTGTGAC	99	17	5.17×10^{-88}	2.54
CCTTCTAA	80	23	1.41×10^{-32}	1.80
CCTTCTGA	101	32	3.20×10^{-34}	1.66
CCTTGACC	77	21	2.42×10^{-34}	1.87
CCTTGACT	68	22	1.05×10^{-22}	1.63
CCTTTTTT	106	35	3.50×10^{-33}	1.60
CGCTAA	129	35	7.55×10^{-57}	1.88
CGCTCAC	125	24	1.95×10^{-94}	2.38
CGCTGA	310	92	2.34×10^{-114}	1.75
CGCTGAC	150	22	5.60×10^{-164}	2.77
CGCTGACC	72	6	6.63×10^{-160}	3.58
CGGGGAGG	69	21	1.13×10^{-25}	1.72
CGGGGTGG	65	18	1.60×10^{-28}	1.85
CGGTGAC	73	19	3.02×10^{-35}	1.94
CGTGACC	93	23	2.97×10^{-48}	2.02
CGTTCTC	69	18	2.76×10^{-33}	1.94
CTAAACTT	65	15	3.95×10^{-38}	2.12
CTAAC	2191	643	0.00	1.77
CTAACA	667	210	2.67×10^{-218}	1.67
CTAACAC	151	42	1.76×10^{-63}	1.85
CTAACAG	149	47	4.55×10^{-50}	1.66
CTAACAT	219	56	3.43×10^{-105}	1.97
CTAACATG	67	8	1.24×10^{-96}	3.07
CTAACATT	81	20	2.31×10^{-42}	2.02
CTAACC	659	179	6.67×10^{-282}	1.88
CTAACCA	185	53	1.79×10^{-73}	1.80
CTAACCAT	66	11	9.22×10^{-62}	2.58
CTAACCC	216	44	3.04×10^{-148}	2.30

Continued on next page...

Table 1 – continued from previous page

Element	Count next to SS	Background count	Chi-square test	LOD
CTAACCCT	86	14	1.62×10^{-82}	2.62
CTAACCT	217	58	8.49×10^{-97}	1.90
CTAACCTC	71	10	6.53×10^{-83}	2.83
CTAACCTG	68	22	1.05×10^{-22}	1.63
CTAACCTT	75	20	9.24×10^{-35}	1.91
CTAACG	186	33	2.74×10^{-156}	2.49
CTAACGC	65	7	1.60×10^{-106}	3.22
CTAACGT	71	12	4.77×10^{-65}	2.56
CTAACT	734	219	2.26×10^{-265}	1.74
CTAACTC	191	55	4.08×10^{-75}	1.80
CTAACTCT	88	19	1.94×10^{-56}	2.21
CTAACTG	224	58	2.47×10^{-105}	1.95
CTAACTGA	64	12	6.21×10^{-51}	2.42
CTAACTGT	70	10	2.81×10^{-80}	2.81
CTAACTT	249	64	2.58×10^{-118}	1.96
CTAACTTT	112	24	3.80×10^{-72}	2.22
CTAATAAT	91	24	1.41×10^{-42}	1.92
CTAATATT	96	31	1.72×10^{-31}	1.63
CTAATCTT	76	18	1.52×10^{-42}	2.08
CTAATG	773	253	1.91×10^{-234}	1.61
CTAATGAT	65	18	1.60×10^{-28}	1.85
CTAATGC	174	48	6.58×10^{-74}	1.86
CTAATGCT	78	22	7.38×10^{-33}	1.83
CTAATGT	251	72	8.71×10^{-99}	1.80
CTAATGTG	66	20	8.15×10^{-25}	1.72
CTAATGTT	95	23	6.03×10^{-51}	2.05
CTAATTCT	98	27	1.66×10^{-42}	1.86
CTAATTGT	76	19	4.47×10^{-39}	2.00
CTATTAAT	66	17	1.43×10^{-32}	1.96
CTCACACC	78	22	7.38×10^{-33}	1.83
CTCACC	1177	372	0.00	1.66
CTCACCAC	78	21	1.62×10^{-35}	1.89
CTCACCAT	64	15	1.09×10^{-36}	2.09
CTCACCC	419	133	8.95×10^{-136}	1.66
CTCACCCC	135	36	3.66×10^{-61}	1.91
CTCACCCT	147	37	4.26×10^{-73}	1.99
CTCACCG	103	24	1.68×10^{-58}	2.10
CTCACCT	428	120	6.05×10^{-174}	1.83
CTCACCTC	144	37	2.90×10^{-69}	1.96
CTCACCTG	158	40	1.10×10^{-77}	1.98
CTCACCTT	111	29	2.34×10^{-52}	1.94
CTCACG	301	92	2.87×10^{-105}	1.71

Continued on next page...

Table 1 – continued from previous page

Element	Count next to SS	Background count	Chi-square test	LOD
CTCACGC	109	20	3.98×10^{-88}	2.45
CTCACGT	80	23	1.41×10^{-32}	1.80
CTCACTC	329	98	1.96×10^{-120}	1.75
CTCACTCA	109	20	3.98×10^{-88}	2.45
CTCACTCC	94	26	1.43×10^{-40}	1.85
CTCACTCT	117	35	1.10×10^{-43}	1.74
CTCACTGA	130	27	1.91×10^{-87}	2.27
CTCACTGC	94	28	1.05×10^{-35}	1.75
CTCACTTG	75	19	8.90×10^{-38}	1.98
CTCATGCC	71	20	3.99×10^{-30}	1.83
CTCATGTG	67	14	1.51×10^{-45}	2.26
CTCATGTT	86	25	3.11×10^{-34}	1.78
CTCATTCC	73	20	2.12×10^{-32}	1.87
CTCCCTGA	124	32	1.79×10^{-59}	1.95
CTCCTAAC	70	9	6.52×10^{-92}	2.96
CTCCTCAC	127	33	3.49×10^{-60}	1.94
CTCCTGAT	89	26	4.56×10^{-35}	1.78
CTCGCTC	78	23	1.90×10^{-30}	1.76
CTCTAAAT	69	22	1.24×10^{-23}	1.65
CTCTAAC	271	49	9.77×10^{-221}	2.47
CTCTAACC	93	12	6.40×10^{-121}	2.95
CTCTAACT	102	17	1.99×10^{-94}	2.58
CTCTAAT	241	73	4.46×10^{-86}	1.72
CTCTAATG	69	14	6.50×10^{-49}	2.30
CTCTAATT	76	24	2.55×10^{-26}	1.66
CTCTCAC	281	93	1.21×10^{-84}	1.60
CTCTCACC	110	27	1.96×10^{-57}	2.03
CTCTGAC	508	113	3.09×10^{-302}	2.17
CTCTGACC	236	35	5.13×10^{-253}	2.75
CTCTGACT	156	36	5.49×10^{-89}	2.12
CTCTGATC	80	19	1.69×10^{-44}	2.07
CTCTGATG	102	18	3.03×10^{-87}	2.50
CTGAC	4042	1343	0.00	1.59
CTGACACC	99	23	1.47×10^{-56}	2.11
CTGACACT	78	23	1.90×10^{-30}	1.76
CTGACATC	66	21	9.25×10^{-23}	1.65
CTGACATG	75	21	4.73×10^{-32}	1.84
CTGACATT	92	28	1.12×10^{-33}	1.72
CTGACC	1574	415	0.00	1.92
CTGACCA	330	96	4.60×10^{-126}	1.78
CTGACCAC	91	25	8.77×10^{-40}	1.86
CTGACCAG	88	28	8.41×10^{-30}	1.65

Continued on next page...

Table 1 – continued from previous page

Element	Count next to SS	Background count	Chi-square test	LOD
CTGACCAT	87	23	1.27×10^{-40}	1.92
CTGACCC	553	123	0.00	2.17
CTGACCCA	111	34	8.16×10^{-40}	1.71
CTGACCCC	186	39	1.63×10^{-122}	2.25
CTGACCCG	66	11	9.22×10^{-62}	2.58
CTGACCCT	217	41	2.52×10^{-166}	2.40
CTGACCG	142	26	1.45×10^{-114}	2.45
CTGACCT	509	134	3.13×10^{-230}	1.93
CTGACCTC	180	45	4.48×10^{-90}	2.00
CTGACCTG	165	37	2.64×10^{-98}	2.16
CTGACCTT	123	35	4.80×10^{-50}	1.81
CTGACG	325	85	2.14×10^{-149}	1.93
CTGACGC	107	18	1.05×10^{-97}	2.57
CTGACGG	110	27	1.96×10^{-57}	2.03
CTGACGT	109	30	3.68×10^{-47}	1.86
CTGACTCA	82	26	4.64×10^{-28}	1.66
CTGACTCT	150	41	5.54×10^{-65}	1.87
CTGACTG	387	112	7.19×10^{-149}	1.79
CTGACTGA	88	28	8.41×10^{-30}	1.65
CTGACTGC	115	24	5.08×10^{-77}	2.26
CTGACTGT	115	28	9.64×10^{-61}	2.04
CTGACTTG	96	24	6.74×10^{-49}	2.00
CTGACTTT	167	41	3.32×10^{-86}	2.03
CTGATCCC	72	21	9.05×10^{-29}	1.78
CTGATCTC	83	25	4.12×10^{-31}	1.73
CTGATCTT	78	22	7.38×10^{-33}	1.83
CTGATGCC	85	24	1.37×10^{-35}	1.82
CTGATGCT	81	26	3.99×10^{-27}	1.64
CTGATGTG	82	26	4.64×10^{-28}	1.66
CTGATTCT	99	32	2.31×10^{-32}	1.63
CTGCTAAC	74	15	2.11×10^{-52}	2.30
CTGCTAAT	70	18	1.55×10^{-34}	1.96
CTGCTCAC	163	31	2.98×10^{-124}	2.39
CTGCTCAT	82	25	4.18×10^{-30}	1.71
CTGCTGAC	155	37	7.83×10^{-84}	2.07
CTGCTGAT	98	31	2.37×10^{-33}	1.66
CTGGCTGA	115	32	9.66×10^{-49}	1.85
CTGGTGAC	76	24	2.55×10^{-26}	1.66
CTGTTCAC	65	21	7.87×10^{-22}	1.63
CTTACTAA	73	16	4.48×10^{-46}	2.19
CTTACTGA	73	21	7.64×10^{-30}	1.80
CTTATTCA	64	20	7.67×10^{-23}	1.68

Continued on next page...

Table 1 – continued from previous page

Element	Count next to SS	Background count	Chi-square test	LOD
CTTCCTAA	80	26	3.30×10^{-26}	1.62
CTTCTAAC	84	18	1.44×10^{-54}	2.22
CTTCTAAT	96	24	6.74×10^{-49}	2.00
CTTCTCAC	100	28	3.65×10^{-42}	1.84
CTTCTGAC	106	23	4.18×10^{-67}	2.20
CTTCTGAT	109	35	6.72×10^{-36}	1.64
CTTGACTC	72	15	4.99×10^{-49}	2.26
CTTGCTGA	108	33	5.88×10^{-39}	1.71
CTTGTAAT	66	16	7.46×10^{-36}	2.04
CTTGTGAC	68	20	7.11×10^{-27}	1.77
CTTTAACT	81	23	1.14×10^{-33}	1.82
CTTTAATG	65	21	7.87×10^{-22}	1.63
GAAACTAA	75	24	2.22×10^{-25}	1.64
GAACTAA	177	52	2.58×10^{-67}	1.77
GAACTAAT	66	13	6.48×10^{-49}	2.34
GACCCCTC	69	20	6.17×10^{-28}	1.79
GACCTCTC	67	19	3.34×10^{-28}	1.82
GACCTGAC	66	19	4.16×10^{-27}	1.80
GACTAAC	126	27	6.24×10^{-81}	2.22
GACTAAT	170	42	7.86×10^{-87}	2.02
GACTGAC	223	52	2.60×10^{-124}	2.10
GACTGACC	86	13	3.81×10^{-91}	2.73
GACTGACT	72	18	4.13×10^{-37}	2.00
GAGCTGAC	71	16	5.09×10^{-43}	2.15
GATATTAA	68	19	2.55×10^{-29}	1.84
GATGCTGA	75	24	2.22×10^{-25}	1.64
GATTAAC	122	32	5.40×10^{-57}	1.93
GCACTAA	142	44	2.15×10^{-49}	1.69
GCACTCAC	68	22	1.05×10^{-22}	1.63
GCACTGAC	102	16	1.55×10^{-102}	2.67
GCCCCCCT	77	24	2.81×10^{-27}	1.68
GCCCCTCA	104	32	4.13×10^{-37}	1.70
GCCCCTGA	124	33	1.62×10^{-56}	1.91
GCCCTCAC	130	26	1.81×10^{-92}	2.32
GCCCTGAC	174	26	3.15×10^{-185}	2.74
GCCCTGAT	91	18	2.38×10^{-66}	2.34
GCCGCTC	74	23	2.06×10^{-26}	1.69
GCCTAAC	126	26	1.23×10^{-85}	2.28
GCCTCAC	339	78	6.02×10^{-192}	2.12
GCCTCACA	66	21	9.25×10^{-23}	1.65
GCCTCACC	125	23	2.22×10^{-100}	2.44
GCCTCACT	112	24	3.80×10^{-72}	2.22

Continued on next page...

Table 1 – continued from previous page

Element	Count next to SS	Background count	Chi-square test	LOD
GCCTCTCA	84	26	5.58×10^{-30}	1.69
GCCTCTGA	116	34	6.41×10^{-45}	1.77
GCCTGAC	340	83	4.42×10^{-175}	2.03
GCCTGACC	144	26	1.76×10^{-118}	2.47
GCCTGACT	94	26	1.43×10^{-40}	1.85
GCGCTGA	79	23	1.67×10^{-31}	1.78
GCTAAC	512	134	6.77×10^{-234}	1.93
GCTAACA	152	44	1.33×10^{-59}	1.79
GCTAACC	141	30	2.57×10^{-91}	2.23
GCTAACG	66	6	1.67×10^{-132}	3.46
GCTAACT	160	45	7.05×10^{-66}	1.83
GCTAATC	108	33	5.88×10^{-39}	1.71
GCTAATGT	71	18	8.23×10^{-36}	1.98
GCTCACAC	65	18	1.60×10^{-28}	1.85
GCTCACC	293	84	4.17×10^{-115}	1.80
GCTCACCC	97	26	4.51×10^{-44}	1.90
GCTCACCT	111	30	1.74×10^{-49}	1.89
GCTCACG	126	24	2.81×10^{-96}	2.39
GCTCACT	277	75	2.45×10^{-120}	1.88
GCTCACTC	78	12	6.25×10^{-81}	2.70
GCTCACTG	102	28	1.93×10^{-44}	1.87
GCTCACTT	65	20	8.10×10^{-24}	1.70
GCTCATGT	65	16	1.68×10^{-34}	2.02
GCTCTAA	172	55	4.52×10^{-56}	1.64
GCTCTAAC	70	7	2.52×10^{-125}	3.32
GCTCTGAC	137	20	7.18×10^{-151}	2.78
GCTCTGAT	84	24	1.73×10^{-34}	1.81
GCTGAC	1030	282	0.00	1.87
GCTGACA	228	74	1.13×10^{-71}	1.62
GCTGACAC	76	14	1.14×10^{-61}	2.44
GCTGACAT	75	14	9.40×10^{-60}	2.42
GCTGACC	360	78	1.00×10^{-223}	2.21
GCTGACCA	87	26	5.54×10^{-33}	1.74
GCTGACCC	143	22	9.48×10^{-147}	2.70
GCTGACCT	111	27	8.77×10^{-59}	2.04
GCTGACG	115	28	9.64×10^{-61}	2.04
GCTGACT	300	78	1.96×10^{-139}	1.94
GCTGACTC	76	18	1.52×10^{-42}	2.08
GCTGACTG	96	18	1.74×10^{-75}	2.42
GCTGACTT	88	22	5.70×10^{-45}	2.00
GCTGATTT	81	24	2.73×10^{-31}	1.75
GCTTACC	93	30	1.29×10^{-30}	1.63

Continued on next page...

Table 1 – continued from previous page

Element	Count next to SS	Background count	Chi-square test	LOD
GGACTGAC	73	9	5.56×10^{-101}	3.02
GGCACTCA	65	18	1.60×10^{-28}	1.85
GGCCCTCA	96	27	3.06×10^{-40}	1.83
GGCCCTGA	160	42	4.46×10^{-74}	1.93
GGCCGGGG	71	22	1.51×10^{-25}	1.69
GGCCTCAC	129	26	9.77×10^{-91}	2.31
GGCCTGA	299	96	2.33×10^{-95}	1.64
GGCCTGAC	132	18	4.90×10^{-159}	2.87
GGCGGGGG	111	35	9.01×10^{-38}	1.67
GGCTAAC	114	23	2.75×10^{-80}	2.31
GGCTCAC	264	87	2.66×10^{-80}	1.60
GGCTCACC	89	19	4.93×10^{-58}	2.23
GGCTCACT	74	23	2.06×10^{-26}	1.69
GGCTCATG	64	20	7.67×10^{-23}	1.68
GGCTCTGA	125	36	8.90×10^{-50}	1.80
GGCTGAC	300	71	1.19×10^{-162}	2.08
GGCTGACC	128	24	5.15×10^{-100}	2.42
GGCTGACT	84	18	1.44×10^{-54}	2.22
GGGACTGA	65	20	8.10×10^{-24}	1.70
GGGAGGGT	126	40	4.12×10^{-42}	1.66
GGGCCGGG	96	24	6.74×10^{-49}	2.00
GGGCCTGA	78	22	7.38×10^{-33}	1.83
GGGCGGGC	70	18	1.55×10^{-34}	1.96
GGGCTCAC	91	23	1.24×10^{-45}	1.98
GGGCTGAC	105	18	1.90×10^{-93}	2.54
GGGGCTGA	105	30	1.12×10^{-42}	1.81
GGGGTGAC	64	20	7.67×10^{-23}	1.68
GGGGTGGC	95	28	9.62×10^{-37}	1.76
GGGTAGGG	76	25	1.98×10^{-24}	1.60
GGGTGGGC	144	40	9.26×10^{-61}	1.85
GGTCCTGA	83	23	6.51×10^{-36}	1.85
GGTCTCAC	74	18	8.85×10^{-40}	2.04
GGTCTGAC	66	10	3.59×10^{-70}	2.72
GGTGACCT	77	24	2.81×10^{-27}	1.68
GTAACATT	80	22	4.01×10^{-35}	1.86
GTAACTTT	88	28	8.41×10^{-30}	1.65
GTCCCTGA	94	24	2.57×10^{-46}	1.97
GTCCTCAC	86	19	2.57×10^{-53}	2.18
GTCCTGAC	92	17	6.17×10^{-74}	2.44
GTCTAAC	109	24	1.95×10^{-67}	2.18
GTCTAAT	144	40	9.26×10^{-61}	1.85
GTCTCAC	228	62	1.16×10^{-98}	1.88

Continued on next page...

Table 1 – continued from previous page

Element	Count next to SS	Background count	Chi-square test	LOD
GTCTCACC	73	13	3.51×10^{-62}	2.49
GTCTCACT	88	22	5.70×10^{-45}	2.00
GTCTCATT	77	23	2.07×10^{-29}	1.74
GTCTGAC	218	56	6.29×10^{-104}	1.96
GTCTGACC	83	16	5.66×10^{-63}	2.38
GTCTGACT	76	20	5.66×10^{-36}	1.93
GTCTGAT	189	60	2.83×10^{-62}	1.66
GTGACTCT	67	22	8.47×10^{-22}	1.61
GTGCTAA	183	48	1.45×10^{-84}	1.93
GTGCTAAC	64	10	2.23×10^{-65}	2.68
GTGCTAAT	71	13	3.18×10^{-58}	2.45
GTGCTCAC	100	17	3.99×10^{-90}	2.56
GTGCTGAC	133	19	8.99×10^{-151}	2.81
GTGCTGAT	87	26	5.54×10^{-33}	1.74
GTGGGGGC	132	41	7.73×10^{-46}	1.69
GTGTAAC	137	36	1.39×10^{-63}	1.93
GTGTAATT	64	17	4.22×10^{-30}	1.91
GTGTCTAA	66	14	6.55×10^{-44}	2.24
GTTAACAT	64	19	5.50×10^{-25}	1.75
GTTAACC	97	31	2.05×10^{-32}	1.65
GTTAATAA	86	28	5.88×10^{-28}	1.62
GTTACTAA	64	12	6.21×10^{-51}	2.42
GTTCTAAT	71	22	1.51×10^{-25}	1.69
GTTCTCAC	67	20	7.81×10^{-26}	1.74
GTTCTGAC	71	12	4.77×10^{-65}	2.56
GTTCTGAT	81	22	2.76×10^{-36}	1.88
GTTTAATG	64	19	5.50×10^{-25}	1.75
GTTTCTAA	114	34	7.71×10^{-43}	1.75
GTTTTAAC	83	23	6.51×10^{-36}	1.85
TAAAATAA	71	21	1.02×10^{-27}	1.76
TAAATTG	72	19	5.13×10^{-34}	1.92
TAACACT	199	60	5.25×10^{-72}	1.73
TAACACTT	77	24	2.81×10^{-27}	1.68
TAACATTT	183	60	8.80×10^{-57}	1.61
TAACCATT	73	20	2.12×10^{-32}	1.87
TAACCCTT	70	15	9.03×10^{-46}	2.22
TAACCTTT	106	29	2.23×10^{-46}	1.87
TAACGC	112	30	1.13×10^{-50}	1.90
TAACGT	197	54	2.40×10^{-84}	1.87
TAACGTG	68	17	3.83×10^{-35}	2.00
TAACGTT	70	15	9.03×10^{-46}	2.22
TAACTAAT	91	20	9.27×10^{-57}	2.19

Continued on next page...

Table 1 – continued from previous page

Element	Count next to SS	Background count	Chi-square test	LOD
TAACTCTT	88	27	7.99×10^{-32}	1.70
TAACTGAT	69	19	1.85×10^{-30}	1.86
TAACTGTG	67	22	8.47×10^{-22}	1.61
TAACTTTG	79	23	1.67×10^{-31}	1.78
TAATAACT	72	22	1.56×10^{-26}	1.71
TAATCATT	88	26	5.12×10^{-34}	1.76
TAATGACT	76	24	2.55×10^{-26}	1.66
TAATGCAT	74	23	2.06×10^{-26}	1.69
TAATGTGT	112	31	6.00×10^{-48}	1.85
TAATTAAC	74	18	8.85×10^{-40}	2.04
TAATTAAT	147	47	3.42×10^{-48}	1.65
TAATTGAT	75	23	2.16×10^{-27}	1.71
TAATTTCT	64	15	1.09×10^{-36}	2.09
TACTAAC	167	33	2.38×10^{-120}	2.34
TACTAACA	64	14	9.93×10^{-41}	2.19
TACTAAT	282	72	3.17×10^{-135}	1.97
TACTAATG	71	15	2.19×10^{-47}	2.24
TACTAATT	102	21	6.45×10^{-70}	2.28
TACTCAC	142	44	2.15×10^{-49}	1.69
TACTGAC	184	48	8.55×10^{-86}	1.94
TACTGACT	71	20	3.99×10^{-30}	1.83
TACTGAT	226	72	1.30×10^{-73}	1.65
TACTGATG	65	19	4.91×10^{-26}	1.77
TATATTA	88	28	8.41×10^{-30}	1.65
TATATTTT	81	25	4.07×10^{-29}	1.70
TATCTAAT	76	24	2.55×10^{-26}	1.66
TATTAAC	250	78	1.77×10^{-84}	1.68
TATTAACT	87	20	9.67×10^{-51}	2.12
TATTAATT	142	44	2.15×10^{-49}	1.69
TATTCTAA	88	28	8.41×10^{-30}	1.65
TATTGATG	70	21	1.10×10^{-26}	1.74
TCACCCTC	87	28	7.16×10^{-29}	1.64
TCACCTCT	112	36	9.04×10^{-37}	1.64
TCACGCT	70	19	1.27×10^{-31}	1.88
TCACTAA	226	65	1.01×10^{-88}	1.80
TCACTAAC	69	6	7.04×10^{-146}	3.52
TCACTAAT	72	18	4.13×10^{-37}	2.00
TCACTCAC	75	15	3.93×10^{-54}	2.32
TCACTCAT	67	20	7.81×10^{-26}	1.74
TCACTCTC	71	23	1.40×10^{-23}	1.63
TCACTGAC	121	20	6.17×10^{-113}	2.60
TCAGTAAC	64	17	4.22×10^{-30}	1.91

Continued on next page...

Table 1 – continued from previous page

Element	Count next to SS	Background count	Chi-square test	LOD
TCATTAAC	66	20	8.15×10^{-25}	1.72
TCCACTCA	73	20	2.12×10^{-32}	1.87
TCCCACTC	81	26	3.99×10^{-27}	1.64
TCCCCCCC	95	23	6.03×10^{-51}	2.05
TCCCCTGA	119	35	9.33×10^{-46}	1.77
TCCCTAAC	65	13	3.75×10^{-47}	2.32
TCCCTCAC	104	23	5.35×10^{-64}	2.18
TCCCTGAC	123	31	2.47×10^{-61}	1.99
TCCCTGAT	73	22	1.55×10^{-27}	1.73
TCCTAAC	238	59	4.03×10^{-120}	2.01
TCCTAACA	70	14	1.21×10^{-50}	2.32
TCCTAACC	69	13	2.12×10^{-54}	2.41
TCCTAACT	86	20	2.73×10^{-49}	2.10
TCCTAAT	259	79	3.42×10^{-91}	1.71
TCCTAATG	79	14	1.34×10^{-67}	2.50
TCCTAATT	80	26	3.30×10^{-26}	1.62
TCCTCAC	352	111	8.20×10^{-116}	1.67
TCCTCACC	123	32	3.16×10^{-58}	1.94
TCCTCACT	117	37	1.66×10^{-39}	1.66
TCCTCTAA	67	17	7.61×10^{-34}	1.98
TCCTGAC	364	120	6.51×10^{-110}	1.60
TCCTGACA	79	22	5.56×10^{-34}	1.84
TCCTGACT	125	33	1.00×10^{-57}	1.92
TCCTGATG	82	26	4.64×10^{-28}	1.66
TCTAAATG	84	27	5.34×10^{-28}	1.64
TCTAAC	745	185	0.00	2.01
TCTAACA	219	59	2.29×10^{-96}	1.89
TCTAACAT	67	14	1.51×10^{-45}	2.26
TCTAACC	209	43	2.18×10^{-141}	2.28
TCTAACCA	70	15	9.03×10^{-46}	2.22
TCTAACCC	65	13	3.75×10^{-47}	2.32
TCTAACCT	87	15	3.84×10^{-77}	2.54
TCTAACT	262	68	2.20×10^{-122}	1.95
TCTAACTC	65	15	3.95×10^{-38}	2.12
TCTAACTG	65	11	1.33×10^{-59}	2.56
TCTAACTT	92	18	3.96×10^{-68}	2.35
TCTAATA	248	75	8.79×10^{-89}	1.73
TCTAATAA	77	23	2.07×10^{-29}	1.74
TCTAATAT	82	26	4.64×10^{-28}	1.66
TCTAATCT	79	24	3.01×10^{-29}	1.72
TCTAATG	274	75	7.58×10^{-117}	1.87
TCTAATGA	85	26	5.79×10^{-31}	1.71

Continued on next page...

Table 1 – continued from previous page

Element	Count next to SS	Background count	Chi-square test	LOD
TCTAATGT	97	19	1.30×10^{-71}	2.35
TCTAATTG	67	20	7.81×10^{-26}	1.74
TCTCACC	317	84	1.41×10^{-142}	1.92
TCTCACCA	77	24	2.81×10^{-27}	1.68
TCTCACCC	115	29	2.07×10^{-57}	1.99
TCTCACCT	132	26	5.51×10^{-96}	2.34
TCTCACG	77	19	2.13×10^{-40}	2.02
TCTCACTG	112	35	9.99×10^{-39}	1.68
TCTCCTAA	77	21	2.42×10^{-34}	1.87
TCTCTAAC	93	16	1.41×10^{-82}	2.54
TCTCTAAT	88	26	5.12×10^{-34}	1.76
TCTCTGAC	141	29	4.51×10^{-96}	2.28
TCTCTGAT	118	37	1.86×10^{-40}	1.67
TCTGAC	1175	372	0.00	1.66
TCTGACC	447	103	7.61×10^{-252}	2.12
TCTGACCA	88	23	7.55×10^{-42}	1.94
TCTGACCC	165	29	1.01×10^{-140}	2.51
TCTGACCT	156	34	3.31×10^{-97}	2.20
TCTGACG	80	21	6.23×10^{-38}	1.93
TCTGACT	401	133	1.83×10^{-119}	1.59
TCTGACTC	121	39	2.20×10^{-39}	1.63
TCTGACTG	121	31	8.96×10^{-59}	1.96
TCTGACTT	145	41	2.54×10^{-59}	1.82
TCTGATGC	67	21	1.04×10^{-23}	1.67
TCTGATTC	85	22	3.94×10^{-41}	1.95
TCTGATTG	75	24	2.22×10^{-25}	1.64
TCTGCTAA	74	23	2.06×10^{-26}	1.69
TCTTAACT	92	25	6.04×10^{-41}	1.88
TCTTACCT	66	18	1.12×10^{-29}	1.87
TCTTGACT	64	19	5.50×10^{-25}	1.75
TCTTTAAC	68	22	1.05×10^{-22}	1.63
TGAACTAA	65	15	3.95×10^{-38}	2.12
TGACCCCT	117	30	8.17×10^{-57}	1.96
TGACCCT	364	113	2.86×10^{-123}	1.69
TGACCCTC	108	24	6.67×10^{-66}	2.17
TGACCCTT	92	24	8.31×10^{-44}	1.94
TGACCGC	77	18	5.79×10^{-44}	2.10
TGACCTCC	84	26	5.58×10^{-30}	1.69
TGACCTCT	118	39	1.12×10^{-36}	1.60
TGACCTGC	76	23	2.16×10^{-28}	1.72
TGACCTGT	72	22	1.56×10^{-26}	1.71
TGACGCC	67	18	7.43×10^{-31}	1.90

Continued on next page...

Table 1 – continued from previous page

Element	Count next to SS	Background count	Chi-square test	LOD
TGACGCT	78	21	1.62×10^{-35}	1.89
TGACTAA	212	67	3.22×10^{-70}	1.66
TGACTAAT	74	18	8.85×10^{-40}	2.04
TGACTCTT	85	24	1.37×10^{-35}	1.82
TGACTGAC	108	20	3.36×10^{-86}	2.43
TGACTGAT	90	22	1.25×10^{-47}	2.03
TGACTGCT	84	27	5.34×10^{-28}	1.64
TGACTTGT	77	23	2.07×10^{-29}	1.74
TGATTAAT	84	27	5.34×10^{-28}	1.64
TGCCTCAC	88	24	5.29×10^{-39}	1.87
TGCCTGAC	110	24	5.47×10^{-69}	2.20
TGCTAAC	234	48	9.13×10^{-159}	2.29
TGCTAACA	75	15	3.93×10^{-54}	2.32
TGCTAACC	69	10	1.10×10^{-77}	2.79
TGCTAACT	83	18	5.56×10^{-53}	2.21
TGCTAAT	344	88	5.54×10^{-164}	1.97
TGCTAATA	73	23	1.89×10^{-25}	1.67
TGCTAATG	116	26	1.01×10^{-69}	2.16
TGCTAATT	93	20	6.73×10^{-60}	2.22
TGCTCAC	372	96	1.37×10^{-174}	1.95
TGCTCACC	126	28	1.41×10^{-76}	2.17
TGCTCACT	125	25	5.50×10^{-89}	2.32
TGCTCATG	89	28	9.53×10^{-31}	1.67
TGCTGAC	447	99	5.20×10^{-268}	2.17
TGCTGACA	114	30	4.37×10^{-53}	1.93
TGCTGACC	151	24	3.58×10^{-148}	2.65
TGCTGACT	136	29	7.47×10^{-88}	2.23
TGCTGATC	68	21	1.11×10^{-24}	1.70
TGGCTCAC	97	27	2.30×10^{-41}	1.85
TGGCTCAT	64	19	5.50×10^{-25}	1.75
TGGCTGAC	85	22	3.94×10^{-41}	1.95
TGGGCTGA	98	28	5.98×10^{-40}	1.81
TGGGGTGG	190	59	3.21×10^{-65}	1.69
TGGTCTGA	74	19	1.68×10^{-36}	1.96
TGGTGACT	86	18	8.17×10^{-58}	2.26
TGTAACTT	94	30	1.52×10^{-31}	1.65
TGTACTCA	64	19	5.50×10^{-25}	1.75
TGTACTGA	72	21	9.05×10^{-29}	1.78
TGTCCTAA	74	20	1.43×10^{-33}	1.89
TGTCTAA	221	71	6.82×10^{-71}	1.64
TGTCTAAC	70	14	1.21×10^{-50}	2.32
TGTCTAAT	86	20	2.73×10^{-49}	2.10

Continued on next page...

Table 1 – continued from previous page

Element	Count next to SS	Background count	Chi-square test	LOD
TGTCTCAC	84	19	2.75×10^{-50}	2.14
TGTCTCAT	79	23	1.67×10^{-31}	1.78
TGTCTGAC	90	21	3.10×10^{-51}	2.10
TGTCTGAT	89	24	3.54×10^{-40}	1.89
TGTCTTAC	66	16	7.46×10^{-36}	2.04
TGTGCTAA	85	17	4.16×10^{-61}	2.32
TGTGCTGA	130	36	2.55×10^{-55}	1.85
TGTGTAAC	80	22	4.01×10^{-35}	1.86
TGTTAATG	87	28	7.16×10^{-29}	1.64
TGTTCTAA	103	32	3.91×10^{-36}	1.69
TGTTGACT	75	24	2.22×10^{-25}	1.64
TGTTTAAC	81	26	3.99×10^{-27}	1.64
TGTTTAAT	151	49	4.26×10^{-48}	1.62
TTAACACT	70	18	1.55×10^{-34}	1.96
TTAACCTT	90	28	1.04×10^{-31}	1.68
TTAACTAA	64	18	2.17×10^{-27}	1.83
TTAACTCA	67	18	7.43×10^{-31}	1.90
TTAACTCT	94	30	1.52×10^{-31}	1.65
TTAACTGA	82	26	4.64×10^{-28}	1.66
TTAACTTA	67	20	7.81×10^{-26}	1.74
TTAATAA	98	31	2.37×10^{-33}	1.66
TTAATCTA	64	18	2.17×10^{-27}	1.83
TTAATTAC	68	21	1.11×10^{-24}	1.70
TTAATTTT	73	24	1.49×10^{-23}	1.60
TTACTAAA	90	24	2.28×10^{-41}	1.91
TTACTAAC	74	13	3.29×10^{-64}	2.51
TTACTAAT	110	27	1.96×10^{-57}	2.03
TTACTGAC	70	18	1.55×10^{-34}	1.96
TTACTGAT	89	27	8.06×10^{-33}	1.72
TTATAACA	80	24	2.93×10^{-30}	1.74
TTATTAAC	89	24	3.54×10^{-40}	1.89
TTCACTAA	74	21	6.16×10^{-31}	1.82
TTCACTGA	89	28	9.53×10^{-31}	1.67
TTCCTAAC	98	19	2.06×10^{-73}	2.37
TTCCTAAT	107	28	2.11×10^{-50}	1.93
TTCCTGAC	117	30	8.17×10^{-57}	1.96
TTCTAAC	314	74	2.68×10^{-171}	2.09
TTCTAACA	107	24	2.19×10^{-64}	2.16
TTCTAACC	70	15	9.03×10^{-46}	2.22
TTCTAACT	133	26	9.08×10^{-98}	2.35
TTCTAAT	481	136	2.37×10^{-192}	1.82
TTCTAATA	118	34	4.74×10^{-47}	1.80

Continued on next page...

Table 1 – continued from previous page

Element	Count next to SS	Background count	Chi-square test	LOD
TTCTAATC	83	16	5.66×10^{-63}	2.38
TTCTAATG	118	27	1.14×10^{-68}	2.13
TTCTAATT	165	50	1.79×10^{-59}	1.72
TTCTCACT	120	35	8.25×10^{-47}	1.78
TTCTGAC	380	111	8.49×10^{-144}	1.78
TTCTGACC	125	27	2.42×10^{-79}	2.21
TTCTGACT	135	41	8.60×10^{-49}	1.72
TTCTGATC	75	20	9.24×10^{-35}	1.91
TTCTTAAC	88	28	8.41×10^{-30}	1.65
TTCTTACC	68	18	4.66×10^{-32}	1.92
TTGACTGA	85	23	3.13×10^{-38}	1.89
TTGATTAA	78	23	1.90×10^{-30}	1.76
TTGCTAA	305	100	2.13×10^{-93}	1.61
TTGCTAAC	69	14	6.50×10^{-49}	2.30
TTGCTAAT	131	31	3.96×10^{-72}	2.08
TTGCTCAC	93	20	6.73×10^{-60}	2.22
TTGCTGAC	94	17	7.86×10^{-78}	2.47
TTGCTGAT	103	32	3.91×10^{-36}	1.69
TTGTAAC	194	61	4.99×10^{-65}	1.67
TTGTATT	127	37	1.55×10^{-49}	1.78
TTGTATTT	66	16	7.46×10^{-36}	2.04
TTGTCTAA	66	20	8.15×10^{-25}	1.72
TTGTTAAC	75	20	9.24×10^{-35}	1.91
TTTAACAA	91	28	1.10×10^{-32}	1.70
TTTAACG	64	15	1.09×10^{-36}	2.09
TTTAACTA	77	25	2.48×10^{-25}	1.62
TTTAATAT	69	21	1.13×10^{-25}	1.72
TTTAATTT	135	44	7.82×10^{-43}	1.62
TTTACTAA	130	33	5.74×10^{-64}	1.98
TTTATTAA	75	21	4.73×10^{-32}	1.84
TTTCCTAA	139	45	1.30×10^{-44}	1.63
TTTCTAAC	158	31	3.65×10^{-115}	2.35
TTTCTAAT	214	54	4.14×10^{-105}	1.99
TTTCTGAC	131	31	3.96×10^{-72}	2.08
TTTCTGAT	168	55	2.01×10^{-52}	1.61
TTTGCTAA	104	31	2.84×10^{-39}	1.75
TTTGTAAC	100	30	2.11×10^{-37}	1.74
TTTTAACC	113	33	4.38×10^{-44}	1.78
TTTTAACT	192	58	2.68×10^{-69}	1.73
TTTTAATA	81	27	2.69×10^{-25}	1.58
TTTTCTAA	251	81	1.40×10^{-79}	1.63
TTTTGACC	64	20	7.67×10^{-23}	1.68

Continued on next page...

Table 1 – continued from previous page

Element	Count next to SS	Background count	Chi-square test	LOD
TTTTTAAC	196	63	5.06×10^{-63}	1.64
**3′SS ISS**				
AAAAAAAG	56	188	6.12×10^{-22}	-1.75
AAAAAAGA	43	156	1.46×10^{-19}	-1.86
AAAAAAGG	19	73	2.61×10^{-10}	-1.94
AAAAAGAG	14	70	2.18×10^{-11}	-2.32
AAAAAGGA	21	74	7.22×10^{-10}	-1.82
AAAAGAAG	18	75	4.64×10^{-11}	-2.06
AAAAGAG	54	171	3.63×10^{-19}	-1.66
AAAAGAGA	19	76	6.22×10^{-11}	-2.00
AAAAGGG	33	129	2.85×10^{-17}	-1.97
AAAATGGA	17	67	1.01×10^{-09}	-1.98
AAACAGG	32	109	1.64×10^{-13}	-1.77
AAAGAAAG	21	118	4.27×10^{-19}	-2.49
AAAGAAG	57	197	1.96×10^{-23}	-1.79
AAAGAGG	47	143	9.89×10^{-16}	-1.61
AAAGGAAG	12	68	1.11×10^{-11}	-2.50
AAAGGAG	34	167	7.64×10^{-25}	-2.30
AAAGGG	140	428	4.64×10^{-44}	-1.61
AAAGGGA	36	157	4.58×10^{-22}	-2.12
AAAGGGAA	13	65	1.12×10^{-10}	-2.32
AAAGGGG	18	105	2.06×10^{-17}	-2.54
AAAGTGG	33	117	8.10×10^{-15}	-1.83
AACCAGG	44	134	7.54×10^{-15}	-1.61
AACCCAGG	22	71	6.05×10^{-09}	-1.69
AAGAAAAG	23	91	1.01×10^{-12}	-1.98
AAGAAAG	72	279	2.83×10^{-35}	-1.95
AAGAAAGA	18	111	1.07×10^{-18}	-2.62
AAGAAAGG	9	68	8.37×10^{-13}	-2.92
AAGAAGA	51	188	1.65×10^{-23}	-1.88
AAGAAGAA	16	76	5.88×10^{-12}	-2.25
AAGAAGC	41	127	2.32×10^{-14}	-1.63
AAGAAGG	31	159	3.27×10^{-24}	-2.36
AAGAAGGA	14	65	2.52×10^{-10}	-2.22
AAGACAG	41	130	5.90×10^{-15}	-1.66
AAGAGAG	38	160	5.15×10^{-22}	-2.07
AAGAGAGA	16	69	1.76×10^{-10}	-2.11
AAGAGCA	42	128	2.92×10^{-14}	-1.61
AAGAGGA	44	180	3.78×10^{-24}	-2.03
AAGAGGAA	15	65	5.58×10^{-10}	-2.12
AAGAGGG	26	133	1.72×10^{-20}	-2.35
AAGATAG	29	95	1.27×10^{-11}	-1.71

Continued on next page...

Table 1 – continued from previous page

Element	Count next to SS	Background count	Chi-square test	LOD
AAGATCC	26	82	6.24×10^{-10}	-1.66
AAGATGG	45	139	1.55×10^{-15}	-1.63
AAGCAAG	34	121	2.59×10^{-15}	-1.83
AAGCAGA	57	178	1.19×10^{-19}	-1.64
AAGCAGG	44	169	6.86×10^{-22}	-1.94
AAGGAAAG	11	65	2.11×10^{-11}	-2.56
AAGGAAG	47	216	1.33×10^{-30}	-2.20
AAGGAAGA	10	69	1.22×10^{-12}	-2.79
AAGGAAGG	10	69	1.22×10^{-12}	-2.79
AAGGAG	184	600	1.05×10^{-64}	-1.71
AAGGAGA	42	185	7.45×10^{-26}	-2.14
AAGGAGAA	16	63	3.19×10^{-09}	-1.98
AAGGAGAG	11	66	1.29×10^{-11}	-2.58
AAGGAGC	36	121	1.10×10^{-14}	-1.75
AAGGAGG	37	183	3.71×10^{-27}	-2.31
AAGGCAG	48	177	3.12×10^{-22}	-1.88
AAGGGAA	55	172	4.60×10^{-19}	-1.64
AAGGGAAA	19	69	1.75×10^{-09}	-1.86
AAGGGAG	34	149	4.45×10^{-21}	-2.13
AAGGGCA	28	113	1.28×10^{-15}	-2.01
AAGGGG	121	400	3.09×10^{-44}	-1.72
AAGGGGA	29	121	6.07×10^{-17}	-2.06
AAGGGGG	12	100	1.37×10^{-18}	-3.06
AAGGTAG	14	88	3.06×10^{-15}	-2.65
AAGTAGA	40	122	1.14×10^{-13}	-1.61
AAGTAGG	18	77	1.77×10^{-11}	-2.10
AAGTGCTG	24	73	9.75×10^{-09}	-1.60
AAGTGGG	33	111	1.33×10^{-13}	-1.75
AATAGGA	25	88	1.87×10^{-11}	-1.82
AATAGGG	14	65	2.52×10^{-10}	-2.22
AATTAGG	21	75	4.50×10^{-10}	-1.84
ACAAGGA	31	104	8.16×10^{-13}	-1.75
ACAAGGG	20	76	1.33×10^{-10}	-1.93
ACACACAC	89	321	2.35×10^{-38}	-1.85
ACAGAGA	52	205	1.18×10^{-26}	-1.98
ACAGAGAA	19	70	1.09×10^{-09}	-1.88
ACAGAGAG	11	67	7.83×10^{-12}	-2.61
ACAGAGG	52	162	5.49×10^{-18}	-1.64
ACAGCCC	20	67	9.35×10^{-09}	-1.74
ACAGGGA	38	125	7.15×10^{-15}	-1.72
ACATAGG	23	70	1.94×10^{-08}	-1.61
ACCAAGG	26	89	2.42×10^{-11}	-1.78

Continued on next page...

Table 1 – continued from previous page

Element	Count next to SS	Background count	Chi-square test	LOD
ACCAGGA	35	118	2.16×10^{-14}	-1.75
ACTAGGA	19	64	1.85×10^{-08}	-1.75
ACTGGGA	38	124	1.13×10^{-14}	-1.71
AGAAAAAG	18	95	2.78×10^{-15}	-2.40
AGAAAAG	77	235	6.53×10^{-25}	-1.61
AGAAAAGA	21	82	1.62×10^{-11}	-1.97
AGAAAAGG	12	64	8.03×10^{-11}	-2.42
AGAAAGA	54	288	2.95×10^{-43}	-2.42
AGAAAGAA	20	121	4.23×10^{-20}	-2.60
AGAAAGAG	4	77	8.85×10^{-17}	-4.27
AGAAAGG	42	199	8.97×10^{-29}	-2.24
AGAAAGGA	10	70	7.42×10^{-13}	-2.81
AGAAAGGG	7	64	1.04×10^{-12}	-3.19
AGAAATGG	22	73	2.38×10^{-09}	-1.73
AGAAGA	208	669	4.45×10^{-71}	-1.69
AGAAGAA	70	232	2.02×10^{-26}	-1.73
AGAAGAAA	21	84	6.24×10^{-12}	-2.00
AGAAGAAG	12	69	6.79×10^{-12}	-2.52
AGAAGAG	46	214	1.57×10^{-30}	-2.22
AGAAGAGA	9	67	1.38×10^{-12}	-2.90
AGAAGAGG	11	69	2.90×10^{-12}	-2.65
AGAAGCAG	10	63	2.43×10^{-11}	-2.66
AGAAGG	188	632	7.97×10^{-70}	-1.75
AGAAGGA	43	223	1.84×10^{-33}	-2.37
AGAAGGAA	14	73	5.00×10^{-12}	-2.38
AGAAGGAG	3	73	2.55×10^{-16}	-4.60
AGAAGGG	37	186	8.69×10^{-28}	-2.33
AGAAGGGA	9	70	3.08×10^{-13}	-2.96
AGAATAG	29	105	1.20×10^{-13}	-1.86
AGAATGG	44	148	1.24×10^{-17}	-1.75
AGACAGA	57	185	4.91×10^{-21}	-1.70
AGACAGG	32	146	3.91×10^{-21}	-2.19
AGACCAG	39	118	3.52×10^{-13}	-1.60
AGACGG	36	110	1.72×10^{-12}	-1.61
AGACTAG	22	67	3.85×10^{-08}	-1.61
AGAGAAAA	27	88	7.89×10^{-11}	-1.70
AGAGAAAG	17	87	6.15×10^{-14}	-2.36
AGAGAAG	48	267	5.78×10^{-41}	-2.48
AGAGAAGA	10	73	1.66×10^{-13}	-2.87
AGAGAAGG	7	76	2.47×10^{-15}	-3.44
AGAGAAGT	20	68	5.85×10^{-09}	-1.77
AGAGACA	42	172	3.66×10^{-23}	-2.03
				Continued on next page...

Table 1 – continued from previous page

Element	Count next to SS	Background count	Chi-square test	LOD
AGAGACAG	10	75	6.11×10^{-14}	-2.91
AGAGAG	189	885	4.25×10^{-121}	-2.23
AGAGAGA	55	409	1.30×10^{-68}	-2.89
AGAGAGAA	16	95	5.26×10^{-16}	-2.57
AGAGAGAG	13	234	2.58×10^{-47}	-4.17
AGAGAGG	30	208	5.34×10^{-35}	-2.79
AGAGAGGA	8	63	4.23×10^{-12}	-2.98
AGAGAGGG	4	64	6.38×10^{-14}	-4.00
AGAGCAA	42	133	3.00×10^{-15}	-1.66
AGAGCAG	64	200	6.77×10^{-22}	-1.64
AGAGCCAG	14	71	1.34×10^{-11}	-2.34
AGAGCTGG	21	75	4.50×10^{-10}	-1.84
AGAGGA	192	614	4.69×10^{-65}	-1.68
AGAGGAA	63	205	3.47×10^{-23}	-1.70
AGAGGAAA	25	80	7.78×10^{-10}	-1.68
AGAGGAAG	10	75	6.11×10^{-14}	-2.91
AGAGGAC	27	95	3.02×10^{-12}	-1.81
AGAGGAG	41	204	3.61×10^{-30}	-2.31
AGAGGAGA	7	63	1.72×10^{-12}	-3.17
AGAGGAGG	11	74	2.41×10^{-13}	-2.75
AGAGGCAG	11	70	1.76×10^{-12}	-2.67
AGAGGG	184	560	7.34×10^{-57}	-1.61
AGAGGGA	46	175	1.81×10^{-22}	-1.93
AGAGGGAG	5	72	2.88×10^{-15}	-3.85
AGAGGGC	38	118	1.77×10^{-13}	-1.63
AGAGGGG	37	134	5.30×10^{-17}	-1.86
AGAGTAG	18	96	1.71×10^{-15}	-2.42
AGAGTGG	35	127	3.24×10^{-16}	-1.86
AGATAG	93	312	2.64×10^{-35}	-1.75
AGATAGA	34	114	6.74×10^{-14}	-1.75
AGATAGG	14	67	9.48×10^{-11}	-2.26
AGATCG	21	80	4.21×10^{-11}	-1.93
AGATGAG	51	171	4.43×10^{-20}	-1.75
AGATGAGG	8	67	5.67×10^{-13}	-3.07
AGATGGA	45	162	3.83×10^{-20}	-1.85
AGATGGG	42	138	3.03×10^{-16}	-1.72
AGATTAG	21	82	1.62×10^{-11}	-1.97
AGCAAAG	39	129	2.29×10^{-15}	-1.73
AGCAAGA	37	134	5.30×10^{-17}	-1.86
AGCAAGG	34	105	4.24×10^{-12}	-1.63
AGCAGAG	58	193	2.53×10^{-22}	-1.73
AGCAGAGA	14	77	6.99×10^{-13}	-2.46

Continued on next page...

Table 1 – continued from previous page

Element	Count next to SS	Background count	Chi-square test	LOD
AGCAGAGG	14	63	6.68×10^{-10}	-2.17
AGCAGAT	37	113	8.70×10^{-13}	-1.61
AGCAGCAG	21	76	2.81×10^{-10}	-1.86
AGCAGGA	50	154	5.26×10^{-17}	-1.62
AGCAGGG	50	179	5.30×10^{-22}	-1.84
AGCATAG	19	67	4.51×10^{-09}	-1.82
AGCCAGGA	9	70	3.08×10^{-13}	-2.96
AGCCTAG	23	75	1.92×10^{-09}	-1.71
AGCTAGA	25	81	4.90×10^{-10}	-1.70
AGCTAGG	15	95	2.25×10^{-16}	-2.66
AGCTCTGG	19	64	1.85×10^{-08}	-1.75
AGCTGGAG	23	69	3.06×10^{-08}	-1.58
AGCTGGGA	11	79	2.00×10^{-14}	-2.84
AGCTGGGG	19	65	1.16×10^{-08}	-1.77
AGGAAAAG	17	67	1.01×10^{-09}	-1.98
AGGAAAG	49	218	2.44×10^{-30}	-2.15
AGGAAAGA	12	69	6.79×10^{-12}	-2.52
AGGAAAGG	9	66	2.28×10^{-12}	-2.87
AGGAAG	242	783	2.62×10^{-83}	-1.69
AGGAAGA	52	243	1.61×10^{-34}	-2.22
AGGAAGAA	14	81	9.73×10^{-14}	-2.53
AGGAAGAG	3	71	7.02×10^{-16}	-4.56
AGGAAGG	47	251	6.06×10^{-38}	-2.42
AGGAAGGA	11	91	5.01×10^{-17}	-3.05
AGGAAGGG	4	69	5.07×10^{-15}	-4.11
AGGACAG	52	159	2.14×10^{-17}	-1.61
AGGAGA	213	670	8.83×10^{-70}	-1.65
AGGAGAA	60	218	1.00×10^{-26}	-1.86
AGGAGAAG	8	66	9.37×10^{-13}	-3.04
AGGAGAG	60	218	1.00×10^{-26}	-1.86
AGGAGAGA	11	74	2.41×10^{-13}	-2.75
AGGAGAGG	19	77	3.85×10^{-11}	-2.02
AGGAGAT	42	126	7.24×10^{-14}	-1.58
AGGAGCA	22	140	2.00×10^{-23}	-2.67
AGGAGG	207	773	3.71×10^{-92}	-1.90
AGGAGGA	29	223	1.36×10^{-38}	-2.94
AGGAGGAA	7	66	3.80×10^{-13}	-3.24
AGGAGGAG	7	86	1.61×10^{-17}	-3.62
AGGAGGG	44	224	2.55×10^{-33}	-2.35
AGGAGGGA	10	73	1.66×10^{-13}	-2.87
AGGAGGGG	7	79	5.46×10^{-16}	-3.50
AGGAGGT	46	141	1.24×10^{-15}	-1.62
				Continued on next page...

Table 1 – continued from previous page

Element	Count next to SS	Background count	Chi-square test	LOD
AGGAGGTG	18	64	8.92×10^{-09}	-1.83
AGGATAG	17	66	1.62×10^{-09}	-1.96
AGGATGG	43	140	2.44×10^{-16}	-1.70
AGGCAAA	41	128	1.47×10^{-14}	-1.64
AGGCAAG	35	129	1.27×10^{-16}	-1.88
AGGCAGA	44	223	4.14×10^{-33}	-2.34
AGGCAGAG	13	103	7.44×10^{-19}	-2.99
AGGCAGG	64	248	1.53×10^{-31}	-1.95
AGGCAGGA	9	87	6.13×10^{-17}	-3.27
AGGCTGGG	23	86	1.09×10^{-11}	-1.90
AGGGAAA	68	206	6.89×10^{-22}	-1.60
AGGGAAAA	18	66	3.45×10^{-09}	-1.87
AGGGAAG	30	214	2.77×10^{-36}	-2.83
AGGGAAGA	17	66	1.62×10^{-09}	-1.96
AGGGAAGG	7	66	3.80×10^{-13}	-3.24
AGGGAG	215	725	4.96×10^{-80}	-1.75
AGGGAGA	54	220	4.45×10^{-29}	-2.03
AGGGAGAA	11	69	2.90×10^{-12}	-2.65
AGGGAGAG	10	79	8.28×10^{-15}	-2.98
AGGGAGG	48	222	1.64×10^{-31}	-2.21
AGGGAGGA	10	63	2.43×10^{-11}	-2.66
AGGGAGGG	15	101	1.15×10^{-17}	-2.75
AGGGCAGG	23	86	1.09×10^{-11}	-1.90
AGGGCTGG	22	66	6.09×10^{-08}	-1.58
AGGGGAA	32	150	5.69×10^{-22}	-2.23
AGGGGAAG	6	73	4.44×10^{-15}	-3.60
AGGGGAG	38	185	3.15×10^{-27}	-2.28
AGGGGAGG	10	76	3.71×10^{-14}	-2.93
AGGGGAT	26	83	3.93×10^{-10}	-1.67
AGGGGCAG	16	70	1.09×10^{-10}	-2.13
AGGGGG	143	450	1.78×10^{-47}	-1.65
AGGGGGA	32	151	3.51×10^{-22}	-2.24
AGGGGGAG	6	64	4.16×10^{-13}	-3.42
AGGGGGG	12	94	2.72×10^{-17}	-2.97
AGGGTAG	20	80	1.97×10^{-11}	-2.00
AGGGTGGG	19	65	1.16×10^{-08}	-1.77
AGGTAAG	28	108	1.38×10^{-14}	-1.95
AGGTAG	92	309	5.15×10^{-35}	-1.75
AGGTAGA	26	96	9.03×10^{-13}	-1.88
AGGTAGC	18	66	3.45×10^{-09}	-1.87
AGGTAGG	16	111	1.93×10^{-19}	-2.79
AGGTATG	28	85	6.30×10^{-10}	-1.60
Continued on next page...				

Table 1 – continued from previous page

Element	Count next to SS	Background count	Chi-square test	LOD
AGGTGAG	60	185	3.91×10^{-20}	-1.62
AGGTGAGG	12	71	2.52×10^{-12}	-2.56
AGGTGGA	38	149	9.57×10^{-20}	-1.97
AGGTGGAG	9	65	3.76×10^{-12}	-2.85
AGGTGGG	56	202	9.33×10^{-25}	-1.85
AGGTGGGA	8	64	2.56×10^{-12}	-3.00
AGGTGGGG	12	67	1.82×10^{-11}	-2.48
AGGTTAG	10	66	5.45×10^{-12}	-2.72
AGGTTGC	31	103	1.30×10^{-12}	-1.73
AGGTTGG	24	107	1.02×10^{-15}	-2.16
AGTACAG	28	104	9.15×10^{-14}	-1.89
AGTAGAA	40	127	1.16×10^{-14}	-1.67
AGTAGAG	19	123	6.75×10^{-21}	-2.69
AGTAGCA	26	79	2.48×10^{-09}	-1.60
AGTAGG	91	284	2.27×10^{-30}	-1.64
AGTAGGA	20	69	3.66×10^{-09}	-1.79
AGTAGGG	9	69	5.08×10^{-13}	-2.94
AGTATAG	15	65	5.58×10^{-10}	-2.12
AGTCAGA	41	136	3.75×10^{-16}	-1.73
AGTCCAG	34	119	6.59×10^{-15}	-1.81
AGTCCCAG	19	63	2.96×10^{-08}	-1.73
AGTCTAG	14	65	2.52×10^{-10}	-2.22
AGTGAGG	44	132	1.86×10^{-14}	-1.58
AGTGCAG	40	122	1.14×10^{-13}	-1.61
AGTGCTGG	12	86	1.47×10^{-15}	-2.84
AGTGGAG	32	139	1.13×10^{-19}	-2.12
AGTGGGA	36	133	4.06×10^{-17}	-1.89
AGTGGGG	32	151	3.51×10^{-22}	-2.24
AGTGTAG	22	72	3.80×10^{-09}	-1.71
AGTTCAG	41	141	3.71×10^{-17}	-1.78
AGTTGGA	32	104	1.66×10^{-12}	-1.70
AGTTGGG	27	118	5.41×10^{-17}	-2.13
ATACAGG	25	79	1.24×10^{-09}	-1.66
ATAGAGG	15	72	1.85×10^{-11}	-2.26
ATAGGAA	36	128	4.22×10^{-16}	-1.83
ATAGGAG	20	66	1.49×10^{-08}	-1.72
ATAGGCA	19	69	1.75×10^{-09}	-1.86
ATAGGG	66	243	6.99×10^{-30}	-1.88
ATAGGGA	19	77	3.85×10^{-11}	-2.02
ATAGGGT	19	70	1.09×10^{-09}	-1.88
ATATGGG	26	83	3.93×10^{-10}	-1.67
ATCAAGG	18	69	8.26×10^{-10}	-1.94
				Continued on next page...

Table 1 – continued from previous page

Element	Count next to SS	Background count	Chi-square test	LOD
ATCAGAG	35	108	2.15×10^{-12}	-1.63
ATCCACC	28	85	6.30×10^{-10}	-1.60
ATCCAGG	30	104	3.97×10^{-13}	-1.79
ATCGAG	23	78	4.74×10^{-10}	-1.76
ATCGGG	20	77	8.26×10^{-11}	-1.94
ATGAAGAA	22	68	2.43×10^{-08}	-1.63
ATGAGAG	32	96	6.49×10^{-11}	-1.58
ATGGAAG	47	142	1.56×10^{-15}	-1.60
ATGGAGG	39	133	3.61×10^{-16}	-1.77
ATGGGAG	33	125	1.89×10^{-16}	-1.92
ATGGGGA	42	173	2.28×10^{-23}	-2.04
ATGGGGG	24	103	7.01×10^{-15}	-2.10
ATGTAGG	18	66	3.45×10^{-09}	-1.87
ATTACAGG	9	71	1.86×10^{-13}	-2.98
ATTAGCA	28	92	2.51×10^{-11}	-1.72
ATTGCAG	36	116	1.10×10^{-13}	-1.69
ATTGGAG	27	81	1.97×10^{-09}	-1.58
ATTGGCA	28	91	3.99×10^{-11}	-1.70
ATTGGGA	28	105	5.71×10^{-14}	-1.91
ATTGGGG	19	75	1.00×10^{-10}	-1.98
ATTTTAGA	23	70	1.94×10^{-08}	-1.61
CAAAAAAA	37	146	1.86×10^{-19}	-1.98
CAAAAGG	26	100	1.36×10^{-13}	-1.94
CAAAGGG	31	104	8.16×10^{-13}	-1.75
CAACAAG	23	73	4.85×10^{-09}	-1.67
CAACAGA	29	103	3.06×10^{-13}	-1.83
CAAGAAG	25	144	3.51×10^{-23}	-2.53
CAAGAGA	37	126	2.21×10^{-15}	-1.77
CAAGAGG	26	110	1.15×10^{-15}	-2.08
CAAGGA	149	448	2.56×10^{-45}	-1.59
CAAGGAA	35	117	3.43×10^{-14}	-1.74
CAAGGAG	27	135	1.47×10^{-20}	-2.32
CAAGGGA	31	104	8.16×10^{-13}	-1.75
CAAGTAG	21	71	2.96×10^{-09}	-1.76
CACACACA	92	359	4.21×10^{-45}	-1.96
CACAGAGA	20	64	3.80×10^{-08}	-1.68
CACTCCAG	23	72	7.70×10^{-09}	-1.65
CACTGCAC	14	65	2.52×10^{-10}	-2.22
CAGAAAAA	27	81	1.97×10^{-09}	-1.58
CAGAAAG	54	164	8.71×10^{-18}	-1.60
CAGAAGA	48	172	3.22×10^{-21}	-1.84
CAGAAGG	42	191	4.20×10^{-27}	-2.19

Continued on next page...

Table 1 – continued from previous page

Element	Count next to SS	Background count	Chi-square test	LOD
CAGAAGGA	4	70	3.06×10^{-15}	-4.13
CAGAGAA	69	230	2.50×10^{-26}	-1.74
CAGAGAAA	22	80	8.89×10^{-11}	-1.86
CAGAGAAG	12	74	5.70×10^{-13}	-2.62
CAGAGAG	49	216	6.36×10^{-30}	-2.14
CAGAGAGA	11	73	3.97×10^{-13}	-2.73
CAGAGAGG	6	67	9.16×10^{-14}	-3.48
CAGAGCAG	19	71	6.77×10^{-10}	-1.90
CAGAGGA	59	205	2.03×10^{-24}	-1.80
CAGAGGAA	16	66	7.52×10^{-10}	-2.04
CAGAGGAG	14	65	2.52×10^{-10}	-2.22
CAGAGGG	62	198	4.23×10^{-22}	-1.68
CAGAGGGA	14	73	5.00×10^{-12}	-2.38
CAGATAG	21	74	7.22×10^{-10}	-1.82
CAGATGAG	8	67	5.67×10^{-13}	-3.07
CAGCAGA	51	168	1.76×10^{-19}	-1.72
CAGCAGCA	22	67	3.85×10^{-08}	-1.61
CAGCTAC	30	97	1.02×10^{-11}	-1.69
CAGGAAG	69	219	3.80×10^{-24}	-1.67
CAGGAAGG	11	73	3.97×10^{-13}	-2.73
CAGGAGA	64	206	4.42×10^{-23}	-1.69
CAGGAGAA	15	71	3.01×10^{-11}	-2.24
CAGGAGAG	17	63	6.81×10^{-09}	-1.89
CAGGAGG	71	251	6.46×10^{-30}	-1.82
CAGGAGGA	10	64	1.48×10^{-11}	-2.68
CAGGAGGC	22	84	1.33×10^{-11}	-1.93
CAGGCTGG	29	105	1.20×10^{-13}	-1.86
CAGGGAA	52	172	5.68×10^{-20}	-1.73
CAGGGAAG	11	65	2.11×10^{-11}	-2.56
CAGGGAGA	19	66	7.23×10^{-09}	-1.80
CAGGGAGG	20	66	1.49×10^{-08}	-1.72
CAGGGGA	39	157	4.61×10^{-21}	-2.01
CAGGGTA	22	84	1.33×10^{-11}	-1.93
CAGGTGGG	18	74	7.52×10^{-11}	-2.04
CATAGAG	26	78	3.91×10^{-09}	-1.58
CATAGGA	19	75	1.00×10^{-10}	-1.98
CATAGGG	20	66	1.49×10^{-08}	-1.72
CATTAGG	20	65	2.38×10^{-08}	-1.70
CCAAAAAA	15	71	3.01×10^{-11}	-2.24
CCAAGAA	35	118	2.16×10^{-14}	-1.75
CCAAGAG	34	111	2.70×10^{-13}	-1.71
CCAAGGA	30	122	8.12×10^{-17}	-2.02
				Continued on next page...

Table 1 – continued from previous page

Element	Count next to SS	Background count	Chi-square test	LOD
CCACTGCA	17	77	8.04×10^{-12}	-2.18
CCAGGAG	66	266	1.42×10^{-34}	-2.01
CCAGGAGA	18	63	1.43×10^{-08}	-1.81
CCAGGAGG	11	74	2.41×10^{-13}	-2.75
CCAGGAGT	13	67	4.19×10^{-11}	-2.37
CCAGGGAG	20	65	2.38×10^{-08}	-1.70
CCATAGA	17	65	2.62×10^{-09}	-1.93
CCATAGG	15	64	9.06×10^{-10}	-2.09
CCCAAAG	51	155	6.61×10^{-17}	-1.60
CCCAGCTA	13	65	1.12×10^{-10}	-2.32
CCCAGGAG	22	77	3.66×10^{-10}	-1.81
CCCAGGGA	22	72	3.80×10^{-09}	-1.71
CCCCGGC	14	68	5.81×10^{-11}	-2.28
CCCGGCC	26	81	9.89×10^{-10}	-1.64
CCCTAGA	22	71	6.05×10^{-09}	-1.69
CCGGGAG	20	78	5.12×10^{-11}	-1.96
CCTAGGA	22	85	8.29×10^{-12}	-1.95
CCTAGGG	20	65	2.38×10^{-08}	-1.70
CCTCCCAA	24	74	6.16×10^{-09}	-1.62
CCTGCAGA	17	63	6.81×10^{-09}	-1.89
CCTGCAGG	17	91	8.66×10^{-15}	-2.42
CCTGGAGA	19	63	2.96×10^{-08}	-1.73
CCTGGAGG	20	79	3.18×10^{-11}	-1.98
CCTGGGAG	19	82	3.47×10^{-12}	-2.11
CCTGGGCA	29	97	5.04×10^{-12}	-1.74
CCTGGGGG	15	81	2.24×10^{-13}	-2.43
CGAACT	19	67	4.51×10^{-09}	-1.82
CGAGAA	21	90	3.51×10^{-13}	-2.10
CGAGGA	45	135	9.47×10^{-15}	-1.58
CGGGAGG	16	67	4.64×10^{-10}	-2.07
CGGGGAG	24	74	6.16×10^{-09}	-1.62
CTACAGG	28	95	6.23×10^{-12}	-1.76
CTACGA	18	65	5.55×10^{-09}	-1.85
CTAGAGA	26	98	3.51×10^{-13}	-1.91
CTAGAGG	17	78	4.95×10^{-12}	-2.20
CTAGGAA	28	106	3.56×10^{-14}	-1.92
CTAGGAG	22	78	2.28×10^{-10}	-1.83
CTAGGAT	23	69	3.06×10^{-08}	-1.58
CTAGGCA	23	74	3.05×10^{-09}	-1.69
CTAGGG	75	228	3.94×10^{-24}	-1.60
CTAGGGA	24	76	2.45×10^{-09}	-1.66
CTATAGA	19	63	2.96×10^{-08}	-1.73
				Continued on next page...

Table 1 – continued from previous page

Element	Count next to SS	Background count	Chi-square test	LOD
CTATGGG	20	69	3.66×10^{-09}	-1.79
CTCCCAAA	13	63	2.99×10^{-10}	-2.28
CTCCCAGG	24	85	3.68×10^{-11}	-1.82
CTGAGGCA	26	85	1.56×10^{-10}	-1.71
CTGCACTC	19	73	2.61×10^{-10}	-1.94
CTGCAGAG	23	78	4.74×10^{-10}	-1.76
CTGCAGG	83	252	1.81×10^{-26}	-1.60
CTGCAGGA	13	65	1.12×10^{-10}	-2.32
CTGCAGGG	19	63	2.96×10^{-08}	-1.73
CTGCAGGT	21	66	3.04×10^{-08}	-1.65
CTGGAAG	57	177	1.88×10^{-19}	-1.63
CTGGAGA	50	192	1.20×10^{-24}	-1.94
CTGGAGAA	13	65	1.12×10^{-10}	-2.32
CTGGAGG	71	234	1.63×10^{-26}	-1.72
CTGGAGGA	8	71	7.61×10^{-14}	-3.15
CTGGGAA	61	197	3.33×10^{-22}	-1.69
CTGGGAAG	21	65	4.83×10^{-08}	-1.63
CTGGGAG	70	241	3.21×10^{-28}	-1.78
CTGGGAGG	13	75	8.11×10^{-13}	-2.53
CTGGGATT	30	97	1.02×10^{-11}	-1.69
CTGGGCAG	23	73	4.85×10^{-09}	-1.67
CTGGGGAG	18	80	4.15×10^{-12}	-2.15
CTGGGTGG	18	65	5.55×10^{-09}	-1.85
CTGTAGA	40	124	4.57×10^{-14}	-1.63
CTGTAGG	27	93	7.70×10^{-12}	-1.78
CTGTGGGA	21	63	1.21×10^{-07}	-1.58
CTGTGTGG	21	66	3.04×10^{-08}	-1.65
CTTCCAG	21	69	7.53×10^{-09}	-1.72
CTTCCCAG	24	73	9.75×10^{-09}	-1.60
CTTGCAG	36	121	1.10×10^{-14}	-1.75
CTTGGAG	44	137	1.93×10^{-15}	-1.64
CTTGGGA	43	157	9.16×10^{-20}	-1.87
CTTGGGAG	8	65	1.55×10^{-12}	-3.02
CTTGGGG	55	168	2.82×10^{-18}	-1.61
CTTTAGA	40	121	1.79×10^{-13}	-1.60
CTTTAGG	30	92	1.02×10^{-10}	-1.62
GAAAAAAG	21	64	7.66×10^{-08}	-1.61
GAAAAAG	55	193	2.97×10^{-23}	-1.81
GAAAAAGA	16	72	4.12×10^{-11}	-2.17
GAAAAGAA	29	90	1.28×10^{-10}	-1.63
GAAAAGG	42	165	1.01×10^{-21}	-1.97
GAAAAGGA	13	64	1.83×10^{-10}	-2.30

Continued on next page...

Table 1 – continued from previous page

Element	Count next to SS	Background count	Chi-square test	LOD
GAAAGAA	77	247	2.85×10^{-27}	-1.68
GAAAGAAA	32	112	4.05×10^{-14}	-1.81
GAAAGAG	43	179	2.83×10^{-24}	-2.06
GAAAGAGA	16	65	1.22×10^{-09}	-2.02
GAAAGG	168	547	4.52×10^{-59}	-1.70
GAAAGGA	41	176	2.53×10^{-24}	-2.10
GAAAGGAA	22	75	9.36×10^{-10}	-1.77
GAAAGGG	19	147	4.69×10^{-26}	-2.95
GAAATGGA	18	69	8.26×10^{-10}	-1.94
GAACAGG	24	98	7.70×10^{-14}	-2.03
GAACTAG	17	64	4.23×10^{-09}	-1.91
GAACTGG	30	103	6.34×10^{-13}	-1.78
GAAGAAG	39	184	1.13×10^{-26}	-2.24
GAAGAAGA	9	68	8.37×10^{-13}	-2.92
GAAGAG	189	567	9.23×10^{-57}	-1.58
GAAGAGA	41	189	4.99×10^{-27}	-2.20
GAAGAGAA	10	66	5.45×10^{-12}	-2.72
GAAGAGG	43	166	1.34×10^{-21}	-1.95
GAAGAGGA	11	63	5.70×10^{-11}	-2.52
GAAGCAG	54	190	5.79×10^{-23}	-1.81
GAAGCAGG	9	63	1.02×10^{-11}	-2.81
GAAGGA	183	593	1.27×10^{-63}	-1.70
GAAGGAA	59	224	2.89×10^{-28}	-1.92
GAAGGAAA	16	85	7.20×10^{-14}	-2.41
GAAGGAAG	13	81	4.17×10^{-14}	-2.64
GAAGGAG	37	206	5.23×10^{-32}	-2.48
GAAGGAGA	6	65	2.51×10^{-13}	-3.44
GAAGGAGG	9	63	1.02×10^{-11}	-2.81
GAAGGCA	40	131	1.85×10^{-15}	-1.71
GAAGGG	151	489	9.41×10^{-53}	-1.70
GAAGGGA	36	185	6.28×10^{-28}	-2.36
GAAGGGG	30	148	3.02×10^{-22}	-2.30
GAAGTAG	31	101	3.27×10^{-12}	-1.70
GAAGTGG	35	128	2.03×10^{-16}	-1.87
GAATGGG	31	107	2.02×10^{-13}	-1.79
GAATTGG	28	86	3.99×10^{-10}	-1.62
GACAGAG	65	199	2.11×10^{-21}	-1.61
GACAGAGA	13	70	9.56×10^{-12}	-2.43
GAGAAAG	56	229	2.87×10^{-30}	-2.03
GAGAAAGA	8	81	5.01×10^{-16}	-3.34
GAGAAAGG	14	65	2.52×10^{-10}	-2.22
GAGAAG	176	652	1.41×10^{-77}	-1.89

Continued on next page...

Table 1 – continued from previous page

Element	Count next to SS	Background count	Chi-square test	LOD
GAGAAGA	34	171	1.10×10^{-25}	-2.33
GAGAAGAA	16	63	3.19×10^{-09}	-1.98
GAGAAGC	31	135	3.52×10^{-19}	-2.12
GAGAAGG	41	219	2.51×10^{-33}	-2.42
GAGAAGGA	9	77	9.23×10^{-15}	-3.10
GAGAAGGG	7	72	1.85×10^{-14}	-3.36
GAGACAG	36	179	1.15×10^{-26}	-2.31
GAGAGA	214	836	1.10×10^{-102}	-1.97
GAGAGAA	58	206	6.21×10^{-25}	-1.83
GAGAGAAA	20	76	1.33×10^{-10}	-1.93
GAGAGAAG	9	74	4.15×10^{-14}	-3.04
GAGAGAG	53	390	2.65×10^{-65}	-2.88
GAGAGAGA	14	223	1.64×10^{-44}	-3.99
GAGAGAGG	6	69	3.34×10^{-14}	-3.52
GAGAGCA	47	143	9.89×10^{-16}	-1.61
GAGAGG	182	547	6.41×10^{-55}	-1.59
GAGAGGA	41	171	2.74×10^{-23}	-2.06
GAGAGGAG	12	67	1.82×10^{-11}	-2.48
GAGAGGG	37	176	1.09×10^{-25}	-2.25
GAGAGGGA	7	63	1.72×10^{-12}	-3.17
GAGATAG	17	74	3.44×10^{-11}	-2.12
GAGATGG	51	158	1.70×10^{-17}	-1.63
GAGCAAG	30	113	5.80×10^{-15}	-1.91
GAGCACA	36	115	1.75×10^{-13}	-1.68
GAGCAGGG	14	63	6.68×10^{-10}	-2.17
GAGCCACC	27	88	7.89×10^{-11}	-1.70
GAGCCAGG	23	78	4.74×10^{-10}	-1.76
GAGCTAG	15	69	7.98×10^{-11}	-2.20
GAGCTGG	78	236	8.20×10^{-25}	-1.60
GAGCTGGA	15	74	6.95×10^{-12}	-2.30
GAGCTGGG	22	81	5.54×10^{-11}	-1.88
GAGGAA	205	636	1.69×10^{-65}	-1.63
GAGGAAAA	20	66	1.49×10^{-08}	-1.72
GAGGAAG	47	220	1.94×10^{-31}	-2.23
GAGGAAGA	10	73	1.66×10^{-13}	-2.87
GAGGAAGG	12	75	3.47×10^{-13}	-2.64
GAGGAG	174	700	5.61×10^{-88}	-2.01
GAGGAGA	46	190	1.51×10^{-25}	-2.05
GAGGAGAA	6	66	1.52×10^{-13}	-3.46
GAGGAGC	38	145	6.33×10^{-19}	-1.93
GAGGAGG	41	272	1.41×10^{-44}	-2.73
GAGGAGGA	9	107	2.69×10^{-21}	-3.57

Continued on next page...

Table 1 – continued from previous page

Element	Count next to SS	Background count	Chi-square test	LOD
GAGGAGGG	8	73	2.79×10^{-14}	-3.19
GAGGATC	25	79	1.24×10^{-09}	-1.66
GAGGCAG	51	258	5.26×10^{-38}	-2.34
GAGGCAGA	10	85	4.12×10^{-16}	-3.09
GAGGCAGG	11	95	6.79×10^{-18}	-3.11
GAGGCGG	16	65	1.22×10^{-09}	-2.02
GAGGCTGG	21	73	1.16×10^{-09}	-1.80
GAGGGA	180	601	4.08×10^{-66}	-1.74
GAGGGAA	41	179	6.03×10^{-25}	-2.13
GAGGGAG	47	246	6.85×10^{-37}	-2.39
GAGGGAGA	10	78	1.36×10^{-14}	-2.96
GAGGGAGG	10	86	2.50×10^{-16}	-3.10
GAGGGCA	39	150	1.26×10^{-19}	-1.94
GAGGGCAG	15	63	1.47×10^{-09}	-2.07
GAGGGGA	38	181	2.17×10^{-26}	-2.25
GAGGGGAG	7	69	8.39×10^{-14}	-3.30
GAGGGGG	36	181	4.36×10^{-27}	-2.33
GAGGTAG	15	79	5.99×10^{-13}	-2.40
GAGGTGG	59	197	8.16×10^{-23}	-1.74
GAGGTGGG	12	73	9.36×10^{-13}	-2.60
GAGGTTG	42	132	4.74×10^{-15}	-1.65
GAGTACA	20	67	9.35×10^{-09}	-1.74
GAGTAG	81	258	3.06×10^{-28}	-1.67
GAGTAGA	21	84	6.24×10^{-12}	-2.00
GAGTAGG	16	65	1.22×10^{-09}	-2.02
GAGTGGG	45	156	6.26×10^{-19}	-1.79
GAGTTGG	31	113	1.22×10^{-14}	-1.87
GATAAGG	22	66	6.09×10^{-08}	-1.58
GATAGAG	17	74	3.44×10^{-11}	-2.12
GATAGGA	20	69	3.66×10^{-09}	-1.79
GATGAGG	36	125	1.71×10^{-15}	-1.80
GATGGAG	46	149	3.22×10^{-17}	-1.70
GATTACAG	14	65	2.52×10^{-10}	-2.22
GATTAGA	25	81	4.90×10^{-10}	-1.70
GATTCG	23	72	7.70×10^{-09}	-1.65
GATTGGG	24	73	9.75×10^{-09}	-1.60
GATTTGAA	21	64	7.66×10^{-08}	-1.61
GCAAAGA	36	109	2.70×10^{-12}	-1.60
GCAAAGG	26	94	2.32×10^{-12}	-1.85
GCAAGAA	21	107	9.24×10^{-17}	-2.35
GCAAGAG	29	107	4.67×10^{-14}	-1.88
GCAAGGA	23	110	1.08×10^{-16}	-2.26

Continued on next page...

Table 1 – continued from previous page

Element	Count next to SS	Background count	Chi-square test	LOD
GCAAGGG	25	83	1.93×10^{-10}	-1.73
GCACAGAG	12	64	8.03×10^{-11}	-2.42
GCAGAAG	51	155	6.61×10^{-17}	-1.60
GCAGAGA	47	179	5.81×10^{-23}	-1.93
GCAGAGAG	12	64	8.03×10^{-11}	-2.42
GCAGAGG	64	213	1.79×10^{-24}	-1.73
GCAGAGGG	18	65	5.55×10^{-09}	-1.85
GCAGCTGG	15	67	2.11×10^{-10}	-2.16
GCAGGA	175	539	2.06×10^{-55}	-1.62
GCAGGAA	38	148	1.54×10^{-19}	-1.96
GCAGGAG	43	217	3.37×10^{-32}	-2.34
GCAGGAGA	7	81	2.00×10^{-16}	-3.53
GCAGGAGG	11	73	3.97×10^{-13}	-2.73
GCAGGCAG	14	66	1.55×10^{-10}	-2.24
GCAGGGA	51	185	6.70×10^{-23}	-1.86
GCAGGGAG	14	74	3.06×10^{-12}	-2.40
GCAGGTGG	20	69	3.66×10^{-09}	-1.79
GCAGTAG	21	75	4.50×10^{-10}	-1.84
GCAGTGAG	22	71	6.05×10^{-09}	-1.69
GCATAGA	20	64	3.80×10^{-08}	-1.68
GCATTAG	22	66	6.09×10^{-08}	-1.58
GCCAAGA	27	105	2.69×10^{-14}	-1.96
GCCAGAA	31	103	1.30×10^{-12}	-1.73
GCCAGGA	47	167	1.60×10^{-20}	-1.83
GCCAGGAG	16	71	6.69×10^{-11}	-2.15
GCCCAGAG	18	64	8.92×10^{-09}	-1.83
GCCCAGGA	20	68	5.85×10^{-09}	-1.77
GCCTGGAG	17	78	4.95×10^{-12}	-2.20
GCCTGGGG	21	79	6.77×10^{-11}	-1.91
GCGAGA	24	80	3.82×10^{-10}	-1.74
GCGGGGG	24	83	9.41×10^{-11}	-1.79
GCTAGGA	17	74	3.44×10^{-11}	-2.12
GCTAGGG	19	64	1.85×10^{-08}	-1.75
GCTGCAGG	21	70	4.72×10^{-09}	-1.74
GCTGGAGG	18	71	3.17×10^{-10}	-1.98
GCTGGGA	54	255	2.46×10^{-36}	-2.24
GCTGGGAT	13	88	1.29×10^{-15}	-2.76
GCTGGGGA	13	65	1.12×10^{-10}	-2.32
GCTGGGGG	22	68	2.43×10^{-08}	-1.63
GCTTGGG	38	116	4.41×10^{-13}	-1.61
GGAAAAG	54	171	3.63×10^{-19}	-1.66
GGAAAGA	54	180	5.90×10^{-21}	-1.74

Continued on next page...

Table 1 – continued from previous page

Element	Count next to SS	Background count	Chi-square test	LOD
GGAAAGG	29	171	1.80×10^{-27}	-2.56
GGAACAG	31	110	4.98×10^{-14}	-1.83
GGAAGA	192	636	2.13×10^{-69}	-1.73
GGAAGAA	52	209	1.78×10^{-27}	-2.01
GGAAGAAA	14	78	4.27×10^{-13}	-2.48
GGAAGAG	39	194	9.08×10^{-29}	-2.31
GGAAGAGA	9	67	1.38×10^{-12}	-2.90
GGAAGAGG	11	72	6.53×10^{-13}	-2.71
GGAAGAT	41	124	9.07×10^{-14}	-1.60
GGAAGG	199	658	1.26×10^{-71}	-1.73
GGAAGGA	52	222	3.72×10^{-30}	-2.09
GGAAGGAA	10	83	1.12×10^{-15}	-3.05
GGAAGGAG	9	74	4.15×10^{-14}	-3.04
GGAAGGG	39	216	2.09×10^{-33}	-2.47
GGAAGGGA	8	65	1.55×10^{-12}	-3.02
GGAAGGGG	8	69	2.08×10^{-13}	-3.11
GGAATAG	16	79	1.36×10^{-12}	-2.30
GGACAAG	24	94	5.20×10^{-13}	-1.97
GGAGAA	216	656	3.65×10^{-66}	-1.60
GGAGAAAA	23	73	4.85×10^{-09}	-1.67
GGAGAAAG	12	63	1.31×10^{-10}	-2.39
GGAGAAG	43	219	1.28×10^{-32}	-2.35
GGAGAAGA	11	64	3.47×10^{-11}	-2.54
GGAGAAGG	12	78	7.83×10^{-14}	-2.70
GGAGAGA	51	220	4.45×10^{-30}	-2.11
GGAGAGAG	13	95	3.99×10^{-17}	-2.87
GGAGAGC	43	134	3.80×10^{-15}	-1.64
GGAGAGG	56	209	3.55×10^{-26}	-1.90
GGAGAGGA	15	70	4.90×10^{-11}	-2.22
GGAGAGGG	14	68	5.81×10^{-11}	-2.28
GGAGATA	27	92	1.23×10^{-11}	-1.77
GGAGATG	51	157	2.67×10^{-17}	-1.62
GGAGCAA	23	84	2.82×10^{-11}	-1.87
GGAGCAG	56	205	2.30×10^{-25}	-1.87
GGAGCAGG	15	64	9.06×10^{-10}	-2.09
GGAGCTGG	12	77	1.29×10^{-13}	-2.68
GGAGGA	150	682	2.83×10^{-92}	-2.18
GGAGGAA	41	181	2.32×10^{-25}	-2.14
GGAGGAAG	8	70	1.26×10^{-13}	-3.13
GGAGGAC	29	96	8.01×10^{-12}	-1.73
GGAGGAG	43	280	1.52×10^{-45}	-2.70
GGAGGAGA	11	71	1.07×10^{-12}	-2.69

Continued on next page...

Table 1 – continued from previous page

Element	Count next to SS	Background count	Chi-square test	LOD
GGAGGAGG	13	109	3.74×10^{-20}	-3.07
GGAGGAT	32	115	9.94×10^{-15}	-1.85
GGAGGCA	39	163	2.66×10^{-22}	-2.06
GGAGGCAG	11	79	2.00×10^{-14}	-2.84
GGAGGCG	14	66	1.55×10^{-10}	-2.24
GGAGGG	215	765	5.11×10^{-88}	-1.83
GGAGGGA	38	213	3.94×10^{-33}	-2.49
GGAGGGAA	10	70	7.42×10^{-13}	-2.81
GGAGGGAG	8	95	4.41×10^{-19}	-3.57
GGAGGGC	47	158	1.04×10^{-18}	-1.75
GGAGGGG	56	224	3.06×10^{-29}	-2.00
GGAGGGGA	15	75	4.26×10^{-12}	-2.32
GGAGGGGG	13	73	2.18×10^{-12}	-2.49
GGAGGTGG	15	79	5.99×10^{-13}	-2.40
GGAGTAG	20	66	1.49×10^{-08}	-1.72
GGAGTGG	30	153	2.67×10^{-23}	-2.35
GGATAGA	16	71	6.69×10^{-11}	-2.15
GGATCAG	23	77	7.56×10^{-10}	-1.74
GGATCG	20	74	3.44×10^{-10}	-1.89
GGATGAG	34	116	2.66×10^{-14}	-1.77
GGATGGA	42	134	1.90×10^{-15}	-1.67
GGATGGG	42	158	2.74×10^{-20}	-1.91
GGATTAC	19	85	8.14×10^{-13}	-2.16
GGATTAG	11	64	3.47×10^{-11}	-2.54
GGATTGG	20	75	2.14×10^{-10}	-1.91
GGCAAG	122	373	1.27×10^{-38}	-1.61
GGCAAGA	23	118	2.22×10^{-18}	-2.36
GGCAAGG	27	101	1.79×10^{-13}	-1.90
GGCAGA	177	585	7.37×10^{-64}	-1.72
GGCAGAA	40	140	2.87×10^{-17}	-1.81
GGCAGAC	31	95	5.15×10^{-11}	-1.62
GGCAGAG	65	245	1.31×10^{-30}	-1.91
GGCAGAGA	6	70	2.02×10^{-14}	-3.54
GGCAGAGG	18	94	4.54×10^{-15}	-2.38
GGCAGGA	53	211	1.47×10^{-27}	-1.99
GGCAGGAG	14	98	2.15×10^{-17}	-2.81
GGCAGGGA	14	70	2.18×10^{-11}	-2.32
GGCATGG	40	130	2.93×10^{-15}	-1.70
GGCGAGG	16	63	3.19×10^{-09}	-1.98
GGCTGGGA	19	63	2.96×10^{-08}	-1.73
GGGAAAG	38	161	3.19×10^{-22}	-2.08
GGGAAG	170	657	1.63×10^{-80}	-1.95
				Continued on next page...

Table 1 – continued from previous page

Element	Count next to SS	Background count	Chi-square test	LOD
GGGAAGA	43	191	9.20×10^{-27}	-2.15
GGGAAGAG	12	64	8.03×10^{-11}	-2.42
GGGAAGG	43	225	6.98×10^{-34}	-2.39
GGGAAGGA	9	66	2.28×10^{-12}	-2.87
GGGAAGGG	9	73	6.85×10^{-14}	-3.02
GGGAAGT	37	118	8.86×10^{-14}	-1.67
GGGAGAA	52	200	1.24×10^{-25}	-1.94
GGGAGAAG	8	82	3.03×10^{-16}	-3.36
GGGAGAG	49	241	3.87×10^{-35}	-2.30
GGGAGAGA	8	67	5.67×10^{-13}	-3.07
GGGAGAGG	12	88	5.42×10^{-16}	-2.87
GGGAGCA	40	129	4.64×10^{-15}	-1.69
GGGAGG	229	823	2.86×10^{-95}	-1.85
GGGAGGA	39	185	7.01×10^{-27}	-2.25
GGGAGGAG	4	78	5.34×10^{-17}	-4.29
GGGAGGG	66	312	4.29×10^{-44}	-2.24
GGGAGGGA	7	88	5.88×10^{-18}	-3.65
GGGAGGGG	12	108	2.52×10^{-20}	-3.17
GGGAGGT	46	139	3.06×10^{-15}	-1.60
GGGAGGTG	17	75	2.12×10^{-11}	-2.14
GGGATGG	47	150	4.09×10^{-17}	-1.67
GGGATGGG	18	63	1.43×10^{-08}	-1.81
GGGATTA	40	121	1.79×10^{-13}	-1.60
GGGATTG	32	98	2.61×10^{-11}	-1.61
GGGCAAG	29	129	1.31×10^{-18}	-2.15
GGGCAGAG	24	75	3.88×10^{-09}	-1.64
GGGCAGGG	31	107	2.02×10^{-13}	-1.79
GGGCGGGG	21	64	7.66×10^{-08}	-1.61
GGGGAA	163	537	1.31×10^{-58}	-1.72
GGGGAAA	49	165	1.70×10^{-19}	-1.75
GGGGAAAA	16	70	1.09×10^{-10}	-2.13
GGGGAAC	26	83	3.93×10^{-10}	-1.67
GGGGAAG	37	199	1.58×10^{-30}	-2.43
GGGGAAGG	6	67	9.16×10^{-14}	-3.48
GGGGAG	199	677	2.13×10^{-75}	-1.77
GGGGAGA	43	193	3.53×10^{-27}	-2.17
GGGGAGAG	11	84	1.65×10^{-15}	-2.93
GGGGAGG	54	279	2.31×10^{-41}	-2.37
GGGGAGGA	5	64	1.64×10^{-13}	-3.68
GGGGAGGG	11	111	2.27×10^{-21}	-3.33
GGGGATG	43	132	9.43×10^{-15}	-1.62
GGGGCAA	22	69	1.53×10^{-08}	-1.65

Continued on next page...

Table 1 – continued from previous page

Element	Count next to SS	Background count	Chi-square test	LOD
GGGGCAGG	28	90	6.34×10^{-11}	-1.68
GGGGGA	139	480	1.24×10^{-54}	-1.79
GGGGGAA	31	140	3.19×10^{-20}	-2.18
GGGGGAG	27	171	3.33×10^{-28}	-2.66
GGGGGAGG	7	73	1.12×10^{-14}	-3.38
GGGGGCA	44	139	7.75×10^{-16}	-1.66
GGGGGCAG	19	67	4.51×10^{-09}	-1.82
GGGGGG	176	533	5.96×10^{-54}	-1.60
GGGGGGA	25	116	2.93×10^{-17}	-2.21
GGGGGGG	47	205	2.57×10^{-28}	-2.12
GGGGGGGG	18	102	8.99×10^{-17}	-2.50
GGGGGTGG	20	82	7.55×10^{-12}	-2.04
GGGGTGGG	25	112	2.02×10^{-16}	-2.16
GGGTAGG	17	82	7.06×10^{-13}	-2.27
GGGTGGA	36	126	1.07×10^{-15}	-1.81
GGGTGGG	96	289	7.11×10^{-30}	-1.59
GGGTGGGG	33	135	1.65×10^{-18}	-2.03
GGGTTAG	14	64	4.10×10^{-10}	-2.19
GGGTTGG	36	125	1.71×10^{-15}	-1.80
GGTAAGG	16	78	2.21×10^{-12}	-2.29
GGTACAG	20	77	8.26×10^{-11}	-1.94
GGTAGAG	23	97	5.75×10^{-14}	-2.08
GGTAGG	72	277	7.25×10^{-35}	-1.94
GGTAGGA	18	70	5.12×10^{-10}	-1.96
GGTAGGG	15	83	8.39×10^{-14}	-2.47
GGTAGGT	19	74	1.62×10^{-10}	-1.96
GGTATGG	18	63	1.43×10^{-08}	-1.81
GGTCAGG	39	126	9.13×10^{-15}	-1.69
GGTGAAG	34	108	1.07×10^{-12}	-1.67
GGTGAGGG	16	64	1.97×10^{-09}	-2.00
GGTGCTGG	22	69	1.53×10^{-08}	-1.65
GGTGGAG	47	171	2.47×10^{-21}	-1.86
GGTGGAGG	10	66	5.45×10^{-12}	-2.72
GGTGGGA	52	187	5.47×10^{-23}	-1.85
GGTGGGAG	15	79	5.99×10^{-13}	-2.40
GGTGGGG	70	264	7.27×10^{-33}	-1.92
GGTGGGGA	8	73	2.79×10^{-14}	-3.19
GGTGGGGG	17	90	1.41×10^{-14}	-2.40
GGTGGTGG	14	66	1.55×10^{-10}	-2.24
GGTGTGG	44	148	1.24×10^{-17}	-1.75
GGTTAAG	25	78	1.96×10^{-09}	-1.64
GGTTAGG	18	65	5.55×10^{-09}	-1.85

Continued on next page...

Table 1 – continued from previous page

Element	Count next to SS	Background count	Chi-square test	LOD
GGTTGCA	23	81	1.16×10^{-10}	-1.82
GGTTGGG	29	121	6.07×10^{-17}	-2.06
GGTTTAG	21	77	1.75×10^{-10}	-1.87
GTAAGGG	17	64	4.23×10^{-09}	-1.91
GTACAGA	28	91	3.99×10^{-11}	-1.70
GTAGAAG	21	89	5.67×10^{-13}	-2.08
GTAGAG	88	316	1.16×10^{-37}	-1.84
GTAGAGA	29	133	1.91×10^{-19}	-2.20
GTAGAGC	21	65	4.83×10^{-08}	-1.63
GTAGAGG	20	78	5.12×10^{-11}	-1.96
GTAGCAG	16	83	1.92×10^{-13}	-2.38
GTAGGA	74	277	3.19×10^{-34}	-1.90
GTAGGAA	20	83	4.67×10^{-12}	-2.05
GTAGGAG	12	73	9.36×10^{-13}	-2.60
GTAGGCA	20	71	1.42×10^{-09}	-1.83
GTAGGG	59	226	1.13×10^{-28}	-1.94
GTAGGGA	16	69	1.76×10^{-10}	-2.11
GTAGGGG	19	67	4.51×10^{-09}	-1.82
GTCAGGA	29	111	7.07×10^{-15}	-1.94
GTCAGGG	38	115	6.95×10^{-13}	-1.60
GTCCAGA	26	88	3.86×10^{-11}	-1.76
GTGAGCCA	17	67	1.01×10^{-09}	-1.98
GTGAGGA	45	147	3.99×10^{-17}	-1.71
GTGAGGG	34	149	4.45×10^{-21}	-2.13
GTGCAGG	51	154	1.04×10^{-16}	-1.59
GTGCTGGG	23	98	3.56×10^{-14}	-2.09
GTGGAAG	38	126	4.51×10^{-15}	-1.73
GTGGAGA	40	150	2.66×10^{-19}	-1.91
GTGGAGG	36	163	2.58×10^{-23}	-2.18
GTGGGA	180	556	2.95×10^{-57}	-1.63
GTGGGAG	41	198	6.55×10^{-29}	-2.27
GTGGGAGG	8	75	1.02×10^{-14}	-3.23
GTGGGGA	47	186	2.14×10^{-24}	-1.98
GTGGGGAG	10	69	1.22×10^{-12}	-2.79
GTGGGGG	51	202	2.29×10^{-26}	-1.99
GTGGGGGG	12	73	9.36×10^{-13}	-2.60
GTGGGTGG	19	71	6.77×10^{-10}	-1.90
GTGGTAG	18	86	2.25×10^{-13}	-2.26
GTGGTGG	62	204	2.72×10^{-23}	-1.72
GTGTAGA	18	71	3.17×10^{-10}	-1.98
GTGTAGG	13	65	1.12×10^{-10}	-2.32
GTGTGGGG	17	64	4.23×10^{-09}	-1.91

Continued on next page...

Table 1 – continued from previous page

Element	Count next to SS	Background count	Chi-square test	LOD
GTTAGG	52	208	2.86×10^{-27}	-2.00
GTTAGGA	15	76	2.61×10^{-12}	-2.34
GTTGCAG	19	94	1.03×10^{-14}	-2.31
GTTGGAG	21	90	3.51×10^{-13}	-2.10
GTTGGGA	26	128	1.95×10^{-19}	-2.30
GTTGGGG	29	109	1.82×10^{-14}	-1.91
GTTGTAG	23	86	1.09×10^{-11}	-1.90
GTTTAGG	19	69	1.75×10^{-09}	-1.86
TAAGGGA	25	77	3.10×10^{-09}	-1.62
TACAGG	118	369	5.05×10^{-39}	-1.64
TACAGGC	17	100	1.04×10^{-16}	-2.56
TACAGGG	24	85	3.68×10^{-11}	-1.82
TACCAGG	24	72	1.54×10^{-08}	-1.58
TAGAACA	31	107	2.02×10^{-13}	-1.79
TAGAAGA	36	117	6.96×10^{-14}	-1.70
TAGAAGC	28	86	3.99×10^{-10}	-1.62
TAGAAGG	27	98	7.38×10^{-13}	-1.86
TAGACAG	23	72	7.70×10^{-09}	-1.65
TAGAGAAA	21	63	1.21×10^{-07}	-1.58
TAGAGAG	30	115	2.25×10^{-15}	-1.94
TAGAGG	102	317	1.41×10^{-33}	-1.64
TAGAGGA	18	91	1.97×10^{-14}	-2.34
TAGAGGC	22	70	9.63×10^{-09}	-1.67
TAGAGGG	18	70	5.12×10^{-10}	-1.96
TAGATAG	16	73	2.53×10^{-11}	-2.19
TAGATGG	27	84	4.99×10^{-10}	-1.64
TAGCAAG	23	84	2.82×10^{-11}	-1.87
TAGCAGA	24	89	5.57×10^{-12}	-1.89
TAGCAGC	24	83	9.41×10^{-11}	-1.79
TAGCTAG	14	64	4.10×10^{-10}	-2.19
TAGCTGG	28	97	2.45×10^{-12}	-1.79
TAGGAAG	27	122	7.89×10^{-18}	-2.18
TAGGACA	16	70	1.09×10^{-10}	-2.13
TAGGAG	91	323	3.97×10^{-38}	-1.83
TAGGAGA	19	108	1.09×10^{-17}	-2.51
TAGGAGG	15	79	5.99×10^{-13}	-2.40
TAGGCAG	12	88	5.42×10^{-16}	-2.87
TAGGCCA	20	63	6.04×10^{-08}	-1.66
TAGGGA	81	312	4.36×10^{-39}	-1.95
TAGGGAA	28	104	9.15×10^{-14}	-1.89
TAGGGAG	14	97	3.53×10^{-17}	-2.79
TAGGGAT	22	75	9.36×10^{-10}	-1.77

Continued on next page...

Table 1 – continued from previous page

Element	Count next to SS	Background count	Chi-square test	LOD
TAGGGCA	19	72	4.21×10^{-10}	-1.92
TAGGGG	77	256	4.66×10^{-29}	-1.73
TAGGGGA	15	83	8.39×10^{-14}	-2.47
TAGGTGC	21	64	7.66×10^{-08}	-1.61
TAGGTGG	19	64	1.85×10^{-08}	-1.75
TAGTACA	22	69	1.53×10^{-08}	-1.65
TAGTAGA	20	91	9.84×10^{-14}	-2.19
TAGTGGG	17	73	5.59×10^{-11}	-2.10
TAGTTGG	22	73	2.38×10^{-09}	-1.73
TATAGG	74	230	8.08×10^{-25}	-1.64
TATAGGA	22	78	2.28×10^{-10}	-1.83
TATGAGG	22	75	9.36×10^{-10}	-1.77
TATGCAG	23	79	2.96×10^{-10}	-1.78
TATGGGG	21	82	1.62×10^{-11}	-1.97
TATGTAG	29	93	3.21×10^{-11}	-1.68
TATTAGC	19	65	1.16×10^{-08}	-1.77
TCAAAAAA	24	94	5.20×10^{-13}	-1.97
TCAAGGG	21	73	1.16×10^{-09}	-1.80
TCAGGGG	35	109	1.36×10^{-12}	-1.64
TCCAAGA	28	104	9.15×10^{-14}	-1.89
TCCAAGG	36	111	1.09×10^{-12}	-1.62
TCCAGAG	49	149	2.56×10^{-16}	-1.60
TCCAGGA	48	171	5.13×10^{-21}	-1.83
TCCCAAAG	11	72	6.53×10^{-13}	-2.71
TCCCAGAG	13	67	4.19×10^{-11}	-2.37
TCCCAGGG	22	73	2.38×10^{-09}	-1.73
TCCCCAGG	23	69	3.06×10^{-08}	-1.58
TCCTAGG	22	92	2.92×10^{-13}	-2.06
TCCTGCAG	21	65	4.83×10^{-08}	-1.63
TCCTGGAG	20	67	9.35×10^{-09}	-1.74
TCCTGGGA	18	64	8.92×10^{-09}	-1.83
TCCTGGGG	16	65	1.22×10^{-09}	-2.02
TCGAAC	27	82	1.25×10^{-09}	-1.60
TCGAGA	24	84	5.88×10^{-11}	-1.81
TCGGGA	35	116	5.44×10^{-14}	-1.73
TCGTAG	21	64	7.66×10^{-08}	-1.61
TCTAGAG	25	95	6.87×10^{-13}	-1.93
TCTAGGG	9	67	1.38×10^{-12}	-2.90
TCTCCAGG	18	64	8.92×10^{-09}	-1.83
TCTGGAG	49	148	4.02×10^{-16}	-1.59
TCTGTAG	36	120	1.74×10^{-14}	-1.74
TGAGCCAC	27	95	3.02×10^{-12}	-1.81

Continued on next page...

Table 1 – continued from previous page

Element	Count next to SS	Background count	Chi-square test	LOD
TGAGGAA	60	197	1.65×10^{-22}	-1.72
TGAGGAAA	23	77	7.56×10^{-10}	-1.74
TGAGGAG	47	162	1.63×10^{-19}	-1.79
TGAGGAGG	6	66	1.52×10^{-13}	-3.46
TGAGGCAG	15	79	5.99×10^{-13}	-2.40
TGCAAGG	27	95	3.02×10^{-12}	-1.81
TGCAGAG	65	204	2.19×10^{-22}	-1.65
TGCAGGA	35	160	4.96×10^{-23}	-2.19
TGCAGGG	48	165	8.33×10^{-20}	-1.78
TGCAGGTG	19	74	1.62×10^{-10}	-1.96
TGCTAGG	16	79	1.36×10^{-12}	-2.30
TGCTGGGA	12	101	8.29×10^{-19}	-3.07
TGGAAGA	43	173	4.88×10^{-23}	-2.01
TGGAAGG	53	162	1.09×10^{-17}	-1.61
TGGAGAA	60	209	6.55×10^{-25}	-1.80
TGGAGAAG	12	81	1.76×10^{-14}	-2.75
TGGAGAG	57	188	1.24×10^{-21}	-1.72
TGGAGCA	42	129	1.86×10^{-14}	-1.62
TGGAGCAG	19	67	4.51×10^{-09}	-1.82
TGGAGG	204	650	1.54×10^{-68}	-1.67
TGGAGGA	30	186	2.67×10^{-30}	-2.63
TGGAGGAG	8	83	1.83×10^{-16}	-3.38
TGGAGGG	43	182	6.78×10^{-25}	-2.08
TGGAGGTG	20	65	2.38×10^{-08}	-1.70
TGGATAG	22	71	6.05×10^{-09}	-1.69
TGGCAAG	27	100	2.87×10^{-13}	-1.89
TGGCAGA	44	151	3.10×10^{-18}	-1.78
TGGCAGAG	11	64	3.47×10^{-11}	-2.54
TGGCCAGG	18	69	8.26×10^{-10}	-1.94
TGGGAAG	45	214	7.12×10^{-31}	-2.25
TGGGAAGA	14	67	9.48×10^{-11}	-2.26
TGGGAAGG	9	64	6.19×10^{-12}	-2.83
TGGGACA	42	130	1.18×10^{-14}	-1.63
TGGGAG	239	720	7.06×10^{-72}	-1.59
TGGGAGA	50	184	5.13×10^{-23}	-1.88
TGGGAGAG	6	66	1.52×10^{-13}	-3.46
TGGGAGG	48	254	3.20×10^{-38}	-2.40
TGGGAGGA	13	69	1.57×10^{-11}	-2.41
TGGGAGGC	12	63	1.31×10^{-10}	-2.39
TGGGAGGG	10	64	1.48×10^{-11}	-2.68
TGGGATTA	18	84	5.96×10^{-13}	-2.22
TGGGCAA	41	133	1.49×10^{-15}	-1.70

Continued on next page...

Table 1 – continued from previous page

Element	Count next to SS	Background count	Chi-square test	LOD
TGGGCAGG	18	86	2.25×10^{-13}	-2.26
TGGGCTGG	19	63	2.96×10^{-08}	-1.73
TGGGGAA	57	187	1.96×10^{-21}	-1.71
TGGGGAAG	11	71	1.07×10^{-12}	-2.69
TGGGGAG	58	262	2.01×10^{-36}	-2.18
TGGGGAGA	14	74	3.06×10^{-12}	-2.40
TGGGGAGG	15	91	1.62×10^{-15}	-2.60
TGGGGCAG	18	65	5.55×10^{-09}	-1.85
TGGGGGA	40	180	1.71×10^{-25}	-2.17
TGGGGGAG	4	64	6.38×10^{-14}	-4.00
TGGGGGG	60	180	3.73×10^{-19}	-1.58
TGGGTGGG	27	94	4.82×10^{-12}	-1.80
TGGTAGA	31	106	3.22×10^{-13}	-1.77
TGGTAGG	19	74	1.62×10^{-10}	-1.96
TGGTGCA	33	103	5.29×10^{-12}	-1.64
TGTAGAG	22	96	4.26×10^{-14}	-2.13
TGTAGG	83	326	2.71×10^{-41}	-1.97
TGTAGGA	23	94	2.42×10^{-13}	-2.03
TGTAGGC	12	64	8.03×10^{-11}	-2.42
TGTAGGG	12	77	1.29×10^{-13}	-2.68
TGTAGGT	29	88	3.18×10^{-10}	-1.60
TGTCAGG	37	120	3.54×10^{-14}	-1.70
TGTGGGA	42	175	8.80×10^{-24}	-2.06
TGTGTAG	39	122	5.71×10^{-14}	-1.65
TGTGTGGG	20	69	3.66×10^{-09}	-1.79
TGTTAGG	13	81	4.17×10^{-14}	-2.64
TTAAGGG	20	67	9.35×10^{-09}	-1.74
TTACAGG	46	139	3.06×10^{-15}	-1.60
TTAGAGG	23	80	1.85×10^{-10}	-1.80
TTAGGAG	23	92	6.30×10^{-13}	-2.00
TTAGGCA	23	69	3.06×10^{-08}	-1.58
TTAGGG	88	292	7.42×10^{-33}	-1.73
TTAGGGA	20	95	1.42×10^{-14}	-2.25
TTAGGGG	16	78	2.21×10^{-12}	-2.29
TTAGTAG	24	90	3.47×10^{-12}	-1.91
TTAGTGG	21	71	2.96×10^{-09}	-1.76
TTCCAGA	47	160	4.12×10^{-19}	-1.77
TTCCAGG	45	168	2.31×10^{-21}	-1.90
TTCCTAG	34	111	2.70×10^{-13}	-1.71
TTCGGA	20	68	5.85×10^{-09}	-1.77
TTCGGG	33	99	3.28×10^{-11}	-1.58
TTCTAGG	26	95	1.45×10^{-12}	-1.87

Continued on next page...

Table 1 – continued from previous page

Element	Count next to SS	Background count	Chi-square test	LOD
TTGCAGG	28	122	1.73×10^{-17}	-2.12
TTGCCAG	40	125	2.90×10^{-14}	-1.64
TTGGAAG	45	153	2.51×10^{-18}	-1.77
TTGGAGG	24	150	7.97×10^{-25}	-2.64
TTGGCAG	36	133	4.06×10^{-17}	-1.89
TTGGGA	183	563	9.72×10^{-58}	-1.62
TTGGGAAG	9	66	2.28×10^{-12}	-2.87
TTGGGAG	30	187	1.64×10^{-30}	-2.64
TTGGGAGG	3	66	8.84×10^{-15}	-4.46
TTGGGCA	35	135	7.51×10^{-18}	-1.95
TTGGGGA	48	163	2.10×10^{-19}	-1.76
TTGGGGAG	10	63	2.43×10^{-11}	-2.66
TTGGGGG	50	154	5.26×10^{-17}	-1.62
TTGGTAG	22	81	5.54×10^{-11}	-1.88
TTGTAGA	43	129	3.67×10^{-14}	-1.58
TTGTAGG	17	81	1.15×10^{-12}	-2.25
TTTAGAG	45	137	3.83×10^{-15}	-1.61
TTTAGGG	21	113	4.94×10^{-18}	-2.43
TTTCCCAG	20	65	2.38×10^{-08}	-1.70
TTTCCTGG	21	67	1.91×10^{-08}	-1.67
TTTGGAG	45	172	3.53×10^{-22}	-1.93
TTTGGAGA	15	63	1.47×10^{-09}	-2.07
TTTGGGA	54	182	2.35×10^{-21}	-1.75
TTTGGGAA	20	65	2.38×10^{-08}	-1.70
TTTTAGG	42	144	1.89×10^{-17}	-1.78
TTTTCCAG	12	72	1.54×10^{-12}	-2.58
